# *IRX3* depletion promotes early cardiac commitment of hiPSC-Derived Cardiomyocytes

**DOI:** 10.1371/journal.pone.0351704

**Published:** 2026-06-16

**Authors:** Agatha Ribeiro Kalthof, Nikolas Dresch Ferreira, Caio Mateus Silva, Iuri Cordeiro Valadão, Iguaracy Pinheiro de Sousa, Ester Riserio Matos Bertoldi, Vanessa Morais Lima, Lauro Thiago Turaca, Ana Beatriz Ruiz Afonso Barbosa, Miriam Helena Fonseca-Alaniz, Jean-Paul Concordet, Elida Adalgisa Neri, Jose E. Krieger

**Affiliations:** 1 Laboratorio de Genetica e Cardiologia Molecular, Instituto do Coracao (InCor), Hospital das Clinicas HCFMUSP, Faculdade de Medicina, Universidade de Sao Paulo, Sao Paulo, Brazil; 2 European Molecular Biology Laboratory, European Bioinformatics Institute, Hinxton, United Kingdom; 3 Laboratoire Structure et Instabilité des Génomes, Inserm U1154, CNRS UMR7196, Muséum National d’Histoire Naturelle, Paris, France; Laboratoire de Biologie du Développement de Villefranche-sur-Mer, FRANCE

## Abstract

Generating mature human induced pluripotent stem cell-derived cardiomyocytes (hiPSC-CMs) remains a major obstacle to accurate disease modeling and cardiac repair. As the transcription factor Irx3 is a key determinant of ventricular conduction system fate in mice, we hypothesized that suppressing *IRX3* expression accelerates human working cardiomyocyte differentiation. Here, we demonstrate that depleting *IRX3* enhances hiPSC-CM differentiation. *IRX3*-knockout (KO) hiPSCs generated a greater number of cardiomyocytes with elevated expression of TNNI1 and CX43. Notably, *IRX3*-KO cardiomyocytes exhibited improved electrophysiological properties, more uniform mitochondrial distribution, better sarcomere organization, and enhanced intercellular connectivity. We observed that *IRX3* expression peaks during the early stages of cardiomyocyte differentiation, whereas *IRX3*-KO cardiac progenitors have increased expression of GATA4, NKX2–5, and *TBX5*, as well as enhanced cell proliferation. These integrative analyses indicate that IRX3 influences cardiomyocyte differentiation by modulating the gene regulatory networks driven by GATA4, NKX2–5, and TBX5, providing functional evidence linking gene regulatory networks to the structural and electrophysiological development of cardiomyocytes. Collectively, these findings identify IRX3 as a key regulator of early cardiac commitment and highlight the potential of IRX3 suppression to enhance the molecular and functional phenotype of hiPSC-derived cardiomyocytes.

## Introduction

Human induced pluripotent stem cell-derived cardiomyocytes (hiPSC-CMs) offer a renewable source of cells for disease modeling, drug testing, and regenerative therapies. Despite significant advances in differentiation protocols [1–6] and the development of 3D models such as cardioids [7], post-transplantation studies have reported myocardial arrhythmias [5,8,9]. These events suggest that hiPSC-CMs often fail to fully mature and integrate into the host myocardium as functionally synchronized units.

The pro-arrhythmic effects associated with stem-cell therapy are likely linked to incomplete CM maturation, impaired electrical coupling, and cellular heterogeneity. In particular, the ectopic emergence of pacemaker- or conduction-like phenotypes from the transplanted cells may lead to abnormal excitability and arrhythmogenesis [5,10]. These arrhythmias have been associated with altered expression of ion channels involved in depolarization and repolarization [10], which differ between working and conduction CMs [11] and are controlled by cardiac transcription factors (TFs) [12,13]. Modulating the activity of these TFs may therefore represent a targeted approach to improve CM subtype specification and reduce arrhythmogenic risks.

At the molecular level, cardiogenic TFs coordinate the formation of specialized cardiac lineages [14–19]. Among them, the Iroquois homeobox (IRX) gene family comprises five TFs expressed in the heart [20], which play pivotal roles in CM fate determination and differentiation [21–26]. In particular, Irx3 and Irx5 act synergistically during murine heart development, but exert distinct functions in shaping the cardiac conduction system [22]. Irx3 physically interacts with Nkx2–5 and Tbx5, and its loss-of-function in the mouse results in conduction abnormalities [24,26]. In human cells, IRX3 has similarly been shown to regulate the expression of NKX2–5, TBX5, and GATA4 [27], TFs that are critical not only for conduction system development but also for cardiogenesis, and which are implicated in congenital heart disease [28,29].

Given the established role of Irx3 in promoting conduction system identity in mouse hearts, and the antagonistic relationship between conduction and working myocardial fates [24], we hypothesized that its suppression might promote the differentiation of working hiPSC-CMs. In this study, we show that *IRX3* depletion improves CM differentiation and enhances early cardiac commitment. Structural and functional assessments indicate that *IRX3*-knockout (KO) hiPSC-CMs exhibit enhanced sarcomere organization, improved mitochondrial distribution, stronger cell-cell coupling, and more robust electrophysiological properties. Furthermore, by integrating transcriptomic profiling of *IRX3*-KO hiPSC-CMs with scATAC and ChIP sequencing data, we provide evidence that *IRX3* modulates the core transcriptional network of NKX2–5, TBX5, and GATA4 to repress key cardiogenic programs. Our findings identify IRX3 as a critical regulator of cardiomyocyte differentiation and suggest that its suppression may be a promising strategy to generate mature, functionally enhanced cardiomyocytes for disease modeling and therapeutic applications.

## Results

### *IRX3* depletion enhances hiPSC-derived cardiomyocyte differentiation

**Fig 1:**
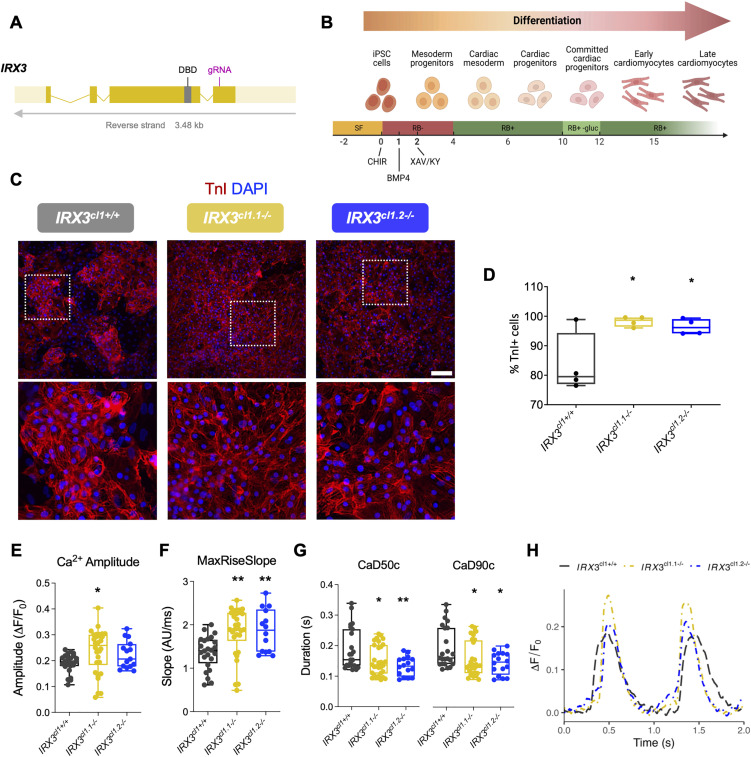
*IRX3* depletion increases hiPSC-derived cardiomyocyte differentiation. **(A-B)** Schematic of CRISPR–Cas9-mediated generation of loss-of-function mutant hiPSC clones *IRX3*^*cl1.1-/-*^, *IRX3*^*cl1.2-/-*^, and *IRX3*^*cl2-/-*^ with respective protocol used for hiPSC-CM differentiation. **(C)** Representative immunofluorescence of cTnI (red) for *IRX3*^*cl1+/+*^, *IRX3*^*cl1.1-/-*^, and *IRX3*^*cl1.2-/-*^ hiPSC-CMs at day 30 of differentiation. **(D)** Percentage of TnI-positive cells (n = 2,000-4,000 cells analyzed per group of 4 independent replicates). Cell nuclei were counterstained with DAPI (blue). (E-H) Calcium transient analysis of *IRX3*^*cl1+/+*^, *IRX3*^*cl1.1-/-*^*,* and *IRX3*^*cl1.2-/-*^ hiPSC-CMs at day 30 of differentiation. **(E)** Calcium transient amplitude. **(F)** Calcium transient maximum slope. **(G)** Corrected calcium transient duration at 50% (CaD_50c_) and 90% (CaD_90c_) of decay, n = 19-22 fields of view of 3 independent replicates. **(H)** Representative curves of calcium transients. Data are presented as box plots with individual replicates. One-way ANOVA. *P < 0.05; **P < 0.01 vs *IRX3*^*cl1+/+*^. Scale bar = 50 µm.

Given the established role of Irx3 in mouse ventricular conduction system specification and maturation *in vivo* [22,24], we investigated whether *IRX3* depletion similarly influences the differentiation of hiPSC-CMs. Using CRISPR–Cas9, we generated from the same mutagenesis experiment two *IRX3* loss-of-function mutant hiPSC lines, hereafter referred to as *IRX3*-KO: *IRX3*^*cl1.1-/-*^ and *IRX3*^*cl1.2-/-*^ (S1 Fig). We targeted the end of *IRX3* exon 1, upstream of the *IRX3* DNA binding domain (Fig 1A). *IRX3*^*cl1.1-/-*^ harbors deletions of 2 and 59 bp in each allele, while *IRX3*^*cl1.2-/-*^ carries deletions of 2 and 227 bp (S1 Fig). All the resulting mutations are in line with previous reports on CRISPR–Cas9 genome editing [30]. Both 2 and 59 bp mutations introduce premature termination codons (PTCs) before the *IRX3* DNA-binding domain (S1 and S2 Figs). The 227 bp deletion encompasses the transcription start site (TSS) and should not be transcribed (S1 Fig); however, an alternative start codon 483 bp downstream may give rise to a truncated protein (S2 Fig). The two *IRX3*-KO hiPSC lines display markedly reduced *IRX3* gene expression levels (S1 Fig). Clone identity and homogeneity were confirmed via subcloning and sequencing (S1 Fig), and pluripotency features remained unaffected across all lines, as demonstrated by karyotyping, expression of pluripotency markers, teratoma formation, and SNP array (S1 and S3 Figs; and S1 Table).

To examine the effects of *IRX3* depletion on CM development, we differentiated hiPSCs using a Wnt-modulation protocol (Fig 1B). At day 30, *IRX3*-KO lines showed ~15% higher CM yield, assessed by immunostaining for the sarcomeric marker cTnI (encoded by *TNNI3*) (Fig 1C-D). Gene expression profiling revealed upregulation of *TNNI1* and *CX43* (*GJA1*), a gap junction protein characteristic of working CMs, in *IRX3*-KO hiPSC-CMs (S4A-B Fig) [31,32]. In contrast, no change was observed in *CX40* (*GJA5*) expression (S4 Fig), while SCN5A, a marker of the ventricular conduction system, was reduced (S4 Fig) [33,34].

As hiPSC-CMs mature, they undergo physiological and metabolic changes that enable a faster and more robust response to stimuli. Calcium handling is central to these changes, governing both contraction and relaxation cycles [35]. In line with a more mature phenotype after *IRX3* depletion, we observed that both *IRX3*^*cl1.1-/-*^ and *IRX3*^*cl1.2-/-*^ clones showed a significant increase in peak amplitude (Fig 1E), faster transient upstroke slope (Fig 1F), and shorter calcium transient duration at 50% and 90% decay (CaD_50c_ and CaD_90c_) compared with *IRX3*^*cl1+/+*^ (Fig 1G), suggesting enhanced calcium handling (Fig 1H).

Together, these findings suggest that *IRX3* depletion improves CM differentiation and may bias subtype specification toward working CMs, consistent with *in vivo* mouse data showing that Irx3 promotes conduction system identity instead of working CM fate [24].

### *IRX3* depletion improves cardiomyocyte function and structure

**Fig 2:**
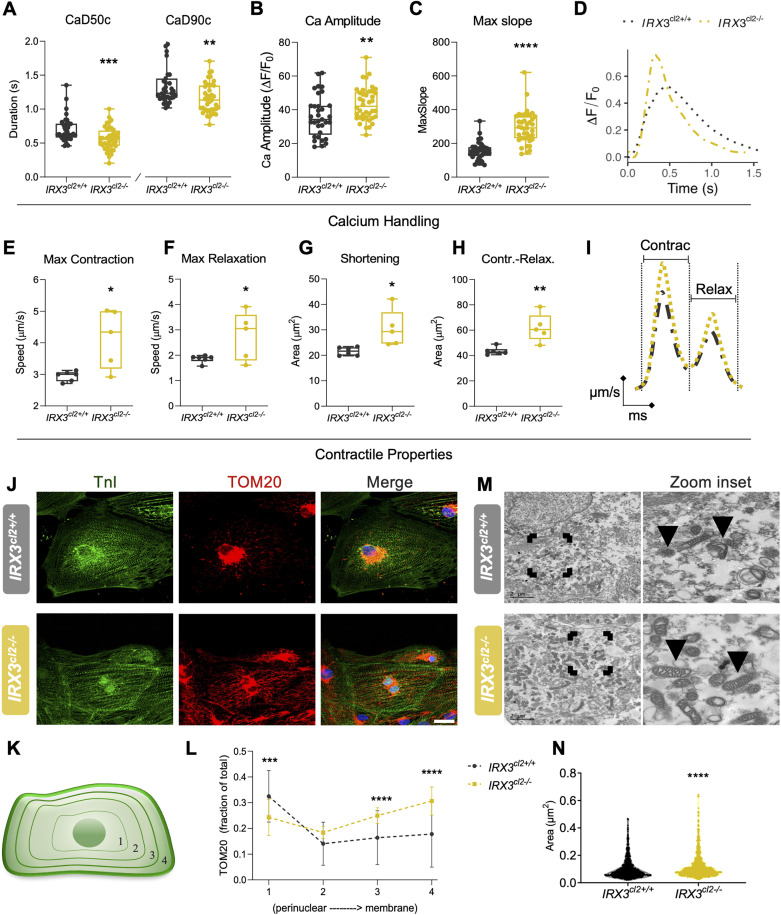
*IRX3*-KO cardiomyocytes display robust excitation-contraction coupling and mitochondrial organization. **(A)** Calcium transient at 50% (CaD_50c_) and 90% (CaD_90c_) of decay. **(B)** Calcium transient amplitude. **(C)** Calcium transient maximum slope, n = 37-40 regions of 2 independent replicates. **(D)** Representative curves of calcium transient. **(E)** Maximum contraction speed. **(F)** Maximum relaxation speed. **(G)** Total shortening area. **(H)** Total contraction-relaxation area, n = 5-6 independent regions. **(I)** Representative curves of contraction and relaxation from *IRX3*^*cl2+/+*^ (black dash line) and *IRX3*^*cl2-/-*^ (gold dash line) hiPSC-CMs at day 30 of differentiation. **(J)** Representative immunofluorescence of TOM20 (red) and endogenous TnI-GFP (green). **(K)** Schematic figure and TOM20 fluorescence distribution analyzed in four regions, ranging from the perinuclear area to membrane. **(L)** TOM20 fluorescence distribution per region (n = 103-127 individually analyzed cells of 2 independent replicates) from *IRX3*^*cl2+/+*^ and *IRX3*^*cl2-/-*^ hiPSC-CMs at day 30 of differentiation. **(M)** Ultrastructure of *IRX3*^*cl2+/+*^ and *IRX3*^*cl2-/-*^ hiPSC-CMs detected by transmission electron microscopy. Black arrows indicate mitochondria further highlighted by zoom inset. **(N)** Quantification of mitochondria area (n = 744-1206 individually analyzed mitochondria of 1 independent experiment) from *IRX3*^*cl2+/+*^ and *IRX3*^*cl2-/-*^ hiPSC-CMs at day 30 of differentiation. Data are presented as box plots (A-C, E-H), mean ± SD **(L)**, and violin plot **(N)**. Student’s t-test was applied to all plots except L, which was analyzed using two-way ANOVA. *P < 0.05; **P < 0.01; ***P < 0.001; and ****P < 0.0001 vs *IRX3*^*cl2+/+*^. Scale bars = 20 µm.

To enable higher-resolution structural and functional analyses, we generated an *IRX3* KO line using a commercial TROPO-GFP hiPSC reporter line (designated *IRX3*^*cl2-/-*^), which carries a homozygous 1 bp insertion (S1-S3 Figs). First, we evaluated whether the enhanced calcium handling observed in previous clones was conserved in this reporter line. We confirmed shortening of CaD_50c_ and CaD_90c_ (Fig 2A), alongside a 19% increase in peak amplitude (Fig 2B), and an 88% faster transient upstroke velocity (Fig 2C), reflecting improved calcium kinetics in the absence of *IRX3* (Fig 2D). These functional improvements were accompanied by the upregulation of *SERCA2* and *RYR2*, the primary regulators of intracellular calcium reuptake and release, respectively [36]. Consistent with this maturation profile, the ventricular isoform *MYH7* was significantly upregulated in *IRX3*^*cl2-/-*^ cardiomyocytes. *MYH6* expression was also increased, while *TNNI3* levels remained comparable to controls (S5 Fig).

Consistent with these calcium handling improvements, action potential durations were significantly reduced in *IRX3*^*cl2-/-*^ hiPSC-CMs by 25% (APD50) and 17% (APD90) compared to controls, with no significant differences observed in action potential amplitude (S5 Fig). Furthermore, contraction and relaxation velocities were increased by 40% and 48%, respectively (Fig 2E-F), while contraction amplitudes were similarly improved (Fig 2G-I). These functional enhancements were accompanied by a more extensive mitochondrial network and increased mitochondrial size (Fig 2J-N), indicative of a shift toward more mature metabolic function.

**Fig 3:**
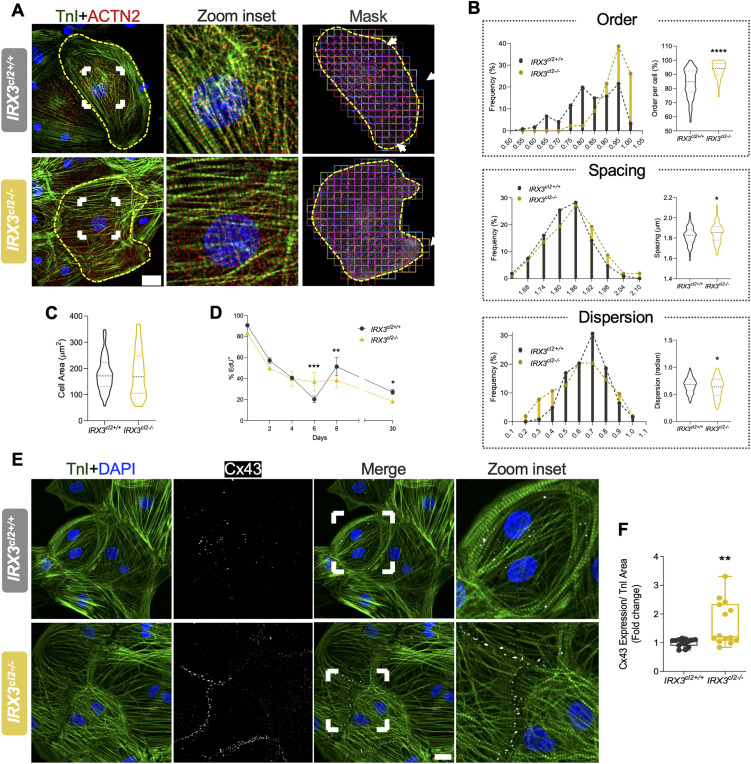
*IRX3* depletion enhances structural maturation of cardiomyocytes. **(A)** Representative immunofluorescence of alpha-Actinin2 (red) and endogenous cTnI-GFP (green). Zoom inset shows the organization of filaments, and the mask of analyzed windows generated with “MorphoScript” software. **(B)** Order, spacing and dispersion of alpha-Actinin2 filaments, calculated using MorphoScript, presented as frequency distribution and violin plots (n = 103-127 individually analyzed cells of 2 independent replicates). **(C)** Total area in µm^2^ presented in violin plots (n = 103-127 individually analyzed cells of 2 independent replicates). **(D)** Proliferation rate from day 0 to day 30 of differentiation (n = 6 independent replicates). **(E)** Representative immunofluorescence of CX43 (white) and endogenous cTnI-GFP (green), and **(F)** Quantification of CX43 expression (n = 14 fields of view obtained from 4 independent replicates) from *IRX3*^*cl2+/+*^ and *IRX3*^*cl2-/-*^ hiPSC-CMs at day 30 of differentiation. Data are presented as violin plots **(B-C)**, mean ± SD **(D)**, and box plots **(F)**. Cell nuclei were counterstained with DAPI (blue). Student’s t-test was applied to all plots, and Mann-Whitney U test for ‘Order’ parameter in Fig 3B. *P < 0.05; **P < 0.01; ***P < 0.001; and ****P < 0.0001 vs *IRX3*^*cl2+/+*^. Scale bars = 20 µm.

Immunostaining for the sarcomeric markers cTnI and alpha-actinin-2 at day 30 revealed enhanced myofibrillar alignment in *IRX3*^*cl2-/-*^ hiPSC-CMs (Fig 3A). Quantitative analysis confirmed a 15% increase in organized areas and a 0.03 μm increase in sarcomere length, alongside reduced sarcomeric dispersion (0.67 ± 0.14 vs. 0.62 ± 0.18; p = 0.035), with no changes in cell size (Fig 3B-C). Proliferation analysis indicated an increase at early stages (day 6) in *IRX3*^*cl2-/-*^ cells that attenuated by later stages, suggesting an initial expansion of the progenitor pool (Fig 3D).

Additionally, CX43 protein levels were upregulated in *IRX3*^*cl2-/-*^ hiPSC-CMs (Fig 3E-F), corroborating the gene expression trends observed in *IRX3*^*cl1.1-/-*^ (S3 Fig). Functional dye transfer assays confirmed enhanced gap junction communication in *IRX3*-KO hiPSC-CMs (S5 Fig). Collectively*,* these data show that *IRX3* depletion improves calcium handling, electrophysiological response, contractility, mitochondrial abundance, sarcomere organization, and intercellular communication, which is consistent with a robust enhancement of CM differentiation.

### *IRX3* depletion activates early cardiogenic gene programs

**Fig 4:**
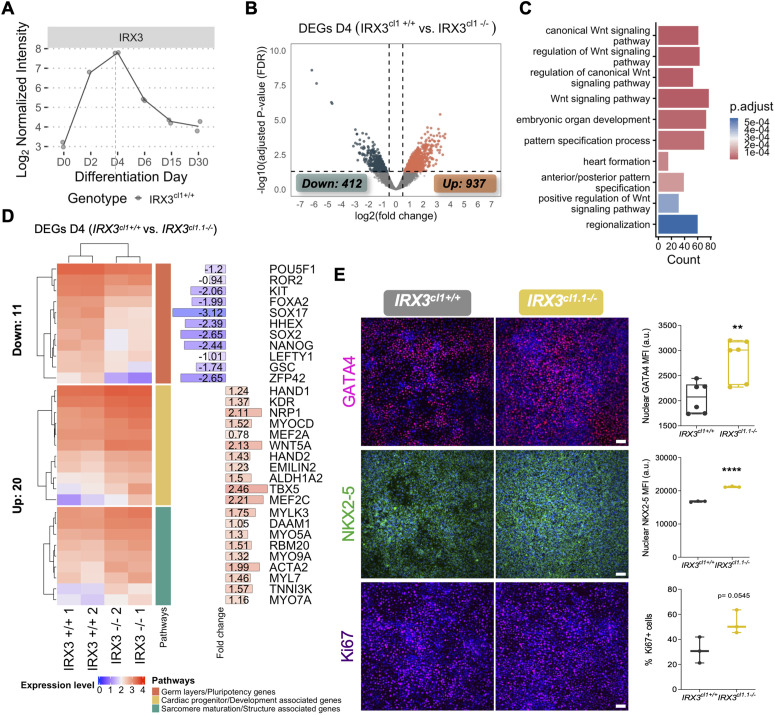
*IRX3* depletion in hiPSCs enhances early cardiac gene expression. **(A)** Gene expression profile of IRX3 in *IRX3*^*cl1+/+*^ clone from days 0 to 15 of differentiation analyzed by microarray. **(B)** Volcano plot showing downregulated (green) and upregulated (orange) differentially expressed genes between *IRX3*^*cl1+/+*^ and *IRX3*^*cl1.1-/-*^ at day 4 of differentiation analyzed by microarray (log2fold change, p.adj < 0.05). **(C)** Gene set enrichment analysis of upregulated genes in *IRX3*^*cl1.1-/-*^ compared with *IRX3*^*cl1+/+*^. **(D)** Heatmap showing 12 downregulated and 19 upregulated selected genes when comparing *IRX3*^*cl1+/+*^ and *IRX3*^*cl1.1-/-*^ at day 4 of differentiation (p.adj < 0.05). **(E)** Representative immunofluorescence of GATA4 (red), NKX2-5 (green), and KI67 (magenta) in *IRX3*^*cl1+/+*^ and *IRX3*^*cl1.1-/-*^ cells at day 6 of differentiation. Cell nuclei were counterstained with DAPI (blue). Quantification of GATA4 and NKX2-5 mean fluorescence intensity (MFI) within the nuclei and percentage of KI67-positive cells (p = 0.0545) (n = 3-6 independent experiments, with data averaged from 5 fields of view per sample and 35,000-36,000 cells analyzed per group). Data are presented as box plots. Student’s t-test. **P < 0.01, ****P < 0.0001 vs. *IRX3*^*cl1+/+*^. Scale bars = 50 µm.

We next assessed the temporal expression profile of *IRX3* in wild-type differentiating hiPSCs. *IRX3* mRNA levels peaked at day 4 of differentiation and declined thereafter (Fig 4A), indicating a potential role in early lineage specification.

Microarray analysis of day 4 *IRX3*^*cl1.1-/-*^ cells revealed 937 upregulated and 412 downregulated genes relative to controls (Fig 4B), consistent with the loss of IRX3’s repressive function [37]. Pathway enrichment analysis revealed significant activation of Wnt-related signaling pathways (Fig 4C), which play key roles in early cardiac mesoderm specification [38,39].

Among the differentially expressed genes, we identified 11 cardiac progenitor markers and 9 genes involved in sarcomere maturation (Fig 4D). Upregulated transcription factors included *TBX5*, *HAND1/2*, *MEF2A/C*, *MYOCD*, *KDR*, and *DAAM1*, all associated with cardiogenesis [40–46]. Conversely, 11 pluripotency and germ-layer–associated genes were downregulated, indicating commitment to the cardiac lineage.

To validate these transcriptomic signatures, we assessed the expression of core cardiac transcription factors at Day 4 (S6 Fig). While *TBX5* and *GATA4* transcripts were consistently upregulated across all *IRX3*-KO clones, *NKX2–5* expression was heterogeneous among clones. However, at the protein level (Day 6), quantitative immunofluorescence revealed a significant increase in nuclear intensity for both GATA4 (+37%) and NKX2–5 (+26%) in *IRX3*^*cl1.1-/-*^ cells relative to controls (Fig 4E). This upregulation of cardiac TFs coincided with a trend toward increased cell proliferation (KI67 + , p = 0.0545), recapitulating phenotypes previously observed in *IRX3*^*cl2-/-*^ progenitors (Fig 3D). Collectively, these data suggest that *IRX3* depletion reinforces the expression of early cardiac lineage determinants, potentially priming the population for robust commitment.

### In silico analyses suggest IRX3 interacts with cardiac transcription factor targets in CPCs

IRX3 is known to physically interact with master cardiac regulators such as TBX5, GATA4, and NKX2–5 during heart development [24,27]. Consistent with these physical associations, large scale network analyses have mapped the broader regulatory landscape connecting these TFs in human cardiogenesis [27]. To investigate whether IRX3 functionally cooperates with these factors to drive gene expression in progenitors, we performed an integrative analysis using scATAC-seq data (day 5) and ChIP-seq data for TBX5, GATA4, and NKX2–5 (day 6).

**Fig 5:**
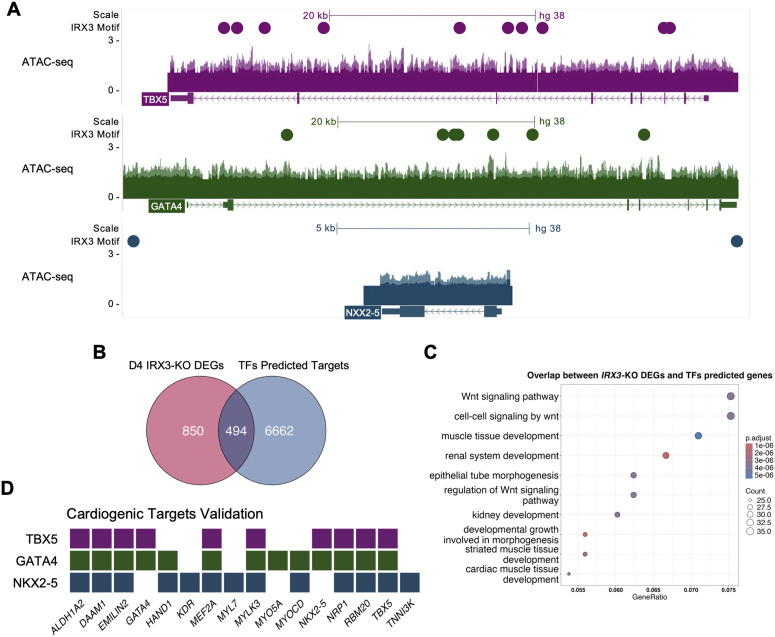
IRX3 modulates cardiac differentiation by interacting with the transcriptional network of TBX5, GATA4, and NKX2-5. **(A)** Genomic alignment of scATAC-seq open chromatin profiles at the *TBX5*, *GATA4*, and *NKX2-5* loci. Predicted IRX3 binding motifs are highlighted with enlarged, color-coded markers to indicate their specific genomic context: markers denote motifs located within open chromatin regions (ATAC-seq peaks) that also physically overlap with ChIP-seq binding peaks for the respective transcription factor. **(B)** Venn diagram intersecting the target genes of TBX5, GATA4, and NKX2-5 (identified by ChIP-seq and containing IRX3 motifs) with the 1,344 differentially expressed genes (DEGs) identified in *IRX3*^*cl1.1-/-*^ at Day 4. **(C)** Gene Ontology (GO) enrichment analysis for biological processes associated with the 494 overlapping genes. **(D)** Specific cardiogenic targets modulated by *IRX3* depletion that are direct downstream targets of at least one of the three core cardiac TFs.

Motif scanning identified putative IRX3 binding sequences within open chromatin regions that were also occupied by these TFs (Fig 5A; see S7 Fig for consensus sequence logos). Of the 26,135 genes in the total IRX3 binding universe, we found that 27% (7,156 genes) were co-bound by at least one cardiac factor, while the majority (18,979 genes) constituted ‘IRX3-Solo’ targets (S8 Fig).

Notably, this distinction was not driven by gene biotype, as both the ‘Solo’ and ‘Co-occupied’ populations contained a similar proportion of protein-coding genes (~60%; S8 Fig). However, functional analysis revealed a clear divergence. While the ‘IRX3-Solo’ fraction was associated with non-cardiac lineages (e.g., sensory perception, immune effector process), the co-occupied subset was exclusively enriched for cardiac-specific processes including muscle development, cell-cell junction assembly, and actin-cytoskeletal organization (S8 Fig). Further classification by molecular function suggested a central role in actin binding and structural integrity (S9 Fig).

Within the co-occupied fraction, NKX2–5 showed the highest overlap (5,067 genes), followed by GATA4 (3,227) and TBX5 (1,997) (S8 Fig). Genomic distribution analysis indicated that the majority of these co-occupied peaks were located within promoter regions (S10 Fig). The subset of 655 genes commonly targeted by all three TFs and IRX3 was significantly enriched for terms related to cardiac morphogenesis (S8 Fig), supporting a model where IRX3 modulates cardiac differentiation by regulating the structural machinery, potentially via TF complexes [27].

To validate these genomic predictions, we intersected the 1,344 DEGs identified in our Day 4 *IRX3*^*cl1.1-/-*^ microarray with the TF binding profile. We found that approximately 37% (494 genes) of the *IRX3*-dependent DEGs correspond to putative *IRX3*/TF co-targets (Fig 5B), a set that includes central regulators of Wnt signaling and muscle development (Fig 5C). Specifically, among the 22 cardiogenesis-associated genes identified earlier (Fig 4D and S6 Fig), 16 are direct targets of at least one TF, and 8 are targeted by all three (Fig 5D). Notably, this shared regulatory network includes the TFs themselves – *TBX5, GATA4* and *NKX2–5* (see S2 Table for the complete gene set).

Altogether, these findings support a model in which IRX3 shapes early cardiac commitment not only by regulating the expression of *TBX5*, *GATA4*, and *NKX2–5*, but by directly modulating their downstream transcriptional programs.

## Discussion

The role of IRX3 in cardiogenesis has been primarily characterized in animal models [24,26]. In this study, we generated *IRX3*-KO hiPSC lines to investigate its function during human CM differentiation. Our results demonstrate that *IRX3* is not essential for CM differentiation; rather, its depletion accelerates early cardiac commitment, reinforces the expression of cardiac transcription factors, and improves the structural and functional development of hiPSC-CMs. While recent network analyses have predicted regulatory interactions between IRX3 and core cardiac transcription factors [27], our study provides the first direct functional demonstration that IRX3 acts as a physiological brake during early cardiogenesis, and that its inhibition acts as a lever to enhance the early differentiation and phenotypic development of hiPSC-CMs.

Cardiac development is orchestrated by a tightly regulated network of TFs [13]. Early in development, these TFs direct stem cells toward a cardiac fate, giving rise to cardiac progenitor cells [47]. We observed that *IRX3* is transiently expressed during the early stages of hiPSC-CM differentiation, and its depletion results in the upregulation of key cardiogenic TFs, including *TBX5*, GATA4, NKX2–5, *MEF2A/C*, and *HAND1/2*, alongside the downregulation of pluripotency-associated genes and activation of Wnt signaling targets. These changes suggest a shift toward robust cardiac lineage commitment and an expansion of the CPC pool, consistent with the increased proliferation observed at day 6. The modulation of Wnt signaling is particularly notable, given its dual role in promoting early mesodermal specification and subsequent CM differentiation [38,39,48].

Our transcriptomic and *in silico* analyses provide a mechanistic basis for this phenotype, identifying *IRX3* as a repressor of the core cardiac regulatory circuitry. Integrative motif and ChIP-seq analyses revealed that nearly 40% of differentially expressed genes in *IRX3*-KO CPCs are potential co-targets of IRX3 and at least one of cardiac TF. Notably, this shared network drives key biological processes such as Wnt signaling, sarcomere assembly, and cell junction organization. Genes such as *ACTN2, TNNI*, and *GJA1* (CX43), markers of structural and functional CM maturation, were upregulated and identified as targets of IRX3-TF co-regulation. These data experimentally validate the role of IRX3 within gene regulatory networks previously predicted to govern hiPSC-CM differentiation [27], supporting a model where IRX3 modulates the phenotype by directly interacting with the core cardiac transcription factor complex [27].

The upregulation of CX43 protein and enhanced intercellular dye transfer in *IRX3*-KO hiPSC-CMs reflect improved electrical coupling, a hallmark of working ventricular myocardium. Interestingly, we did not observe changes in CX40 (*GJA5*) expression, contrasting with findings in *IRX5*-deficient models where CX40 is downregulated, impairing depolarization [22,49]. These divergences highlight the distinct regulatory roles of *IRX3* and *IRX5* in modulating ion channel expression, mirroring functional distinctions observed in murine models [22,26].

*IRX3* depletion also significantly advanced structural and metabolic maturation. Sarcomere organization was enhanced in *IRX3*-KO hiPSC-CMs, characterized by increased alignment and spacing of alpha-actinin-2 filaments. These structural improvements were accompanied by larger and more interconnected mitochondria, suggesting a metabolic shift essential for supporting the increased contractile demands of mature cardiomyocytes [50–53].

Functionally, *IRX3*-KO hiPSC-CMs exhibited a phenotype consistent with the transition from immature to working myocardium. The combination of enhanced calcium transient amplitude and shortened decay times indicates a refinement of excitation-contraction coupling, likely reflecting improved sarcoplasmic reticulum calcium handling [35,51]. This functional maturation was further evidenced by shortened action potential durations and improved mechanical performance, hallmarks of a more efficient contractile machinery. Furthermore, the reduced proliferation rate observed at later differentiation stages aligns with the canonical maturation trajectory, where cardiomyocytes withdraw from the cell cycle to acquire advanced functional competence [54].

Overall, our study demonstrates that *IRX3* acts as a brake on early cardiac differentiation. Its suppression in hiPSCs facilitates the robust activation of cardiac transcriptional programs, translating into enhanced sarcomere structure, mitochondrial organization, calcium handling, electrical connectivity, and mechanical function. Despite these compelling findings, our study has limitations. First, while we demonstrate an improved functional phenotype *in vitro*, the *in vivo* behavior of *IRX3*-KO hiPSC-CMs, including engraftment efficiency and long-term arrhythmogenic risk, remains to be defined in transplantations models.

Second, regarding genomic integrity, although CRISPR–Cas9 editing was designed to minimize off-target effects, we cannot completely exclude the possibility of unintended alterations. However, we observed a consistent phenotype across three independent *IRX3*-KO clones, which also showed ≥ 98.9% SNV concordance vs. their parental clones. Thus, these data strongly suggest that the observed effects are driven by the depletion of *IRX3* rather than clone-specific off-target artifacts. Future clinical translation would nonetheless require comprehensive whole-genome sequencing.

Third, *IRX3* is known to interact with other cardiac transcription factors such as *IRX5* and *TBX5*, raising the possibility of compensatory mechanisms. Moreover, given the broad expression of *IRX3* across diverse lineages (neurogenesis, adipogenesis, and osteogenesis), its depletion may also influence early stem cell fate decisions more generally. Because our study relied on constitutive KO lines, we cannot exclude lineage-nonspecific effects. While our *in silico* integration infers rather than proves physical co-occupancy, these predictions align with established interactions between IRX3 and core cardiac factors [27]. Finally, our bioinformatic integration utilized public datasets from slightly different developmental time points (Day 5 ATAC-seq and Day 6 ChIP-seq) than our transcriptomic profiling (Day 4). While the robust enrichment of cardiac ontologies suggests conservation of the regulatory logic, this temporal offset limit our resolution of rapid dynamic changes during the mesoderm-to-cardiac transition.

Looking forward, identifying *IRX3* as a molecular barrier to early cardiac commitment and subsequent development opens opportunities for combinatorial strategies. Integrating *IRX3* suppression with mechanical conditioning, electrical pacing, or metabolic optimization could synergistically enhance electrophysiological fidelity. Furthermore, developing temporally controlled or inducible *IRX3* modulation strategies would allow for the fine-tuning of differentiation kinetics while minimizing the risks associated with permanent gene disruption.

In summary, our findings position *IRX3* suppression as a potent tool to improve the generation of robustly differentiated hiPSC-derived cardiomyocytes, providing a new strategy to overcome the maturation bottleneck in cardiovascular research and therapy.

## Materials and methods

A complete list of guide RNA sequences, qPCR primers, and reagents used in this study, along with their sources and catalog numbers, is provided in S3–S5 Tables.

### Ethics Statement

This study involved two human induced pluripotent stem cells (hiPSCs) lines and animal research, all conducted under approved ethical guidelines.

Donor-derived hiPSC line (SS109 clone): hiPSCs were generated from adult skin fibroblasts after obtaining written informed consent from the donor. The consent process and study were reviewed and approved by the Research Projects Ethics Committee (CAPPesq) of the Clinical Directorate of Hospital das Clínicas, Faculty of Medicine, University of São Paulo, under protocol number 0055/11, approved on March 21, 2012. Project title: Use of iPS (induced pluripotent stem) cells to understand changes in cardiomyocytes of patients with genetically based cardiomyopathies [55].

Commercial hiPSC line (AICS-0037–172): We used the AICS-0037–172 line developed at the Allen Institute for Cell Science (allencell.org/cell-catalog) and available through the Coriell Institute for Medical Research. This line is derived from the WTC parental line (Coriell Cat#GM25256), originally developed by the Conklin Laboratory at the J. David Gladstone Institute. The cell line was generated with IRB approval and written informed consent from the donor, and all identifying information was removed prior to distribution. The use of this line in our study complies with the terms outlined by the Allen Cell Collection (https://catalog.coriell.org/1/AllenCellCollection/MTA-and-Additional-Terms). Use of this line was reviewed and approved by the Committee for Ethics in Animal Use (CEUA) of the University of São Paulo Medical School under protocol number 1656/2021, approved on November 24, 2021. Project title: Cardiomyocytes Derived from Human iPSC (hiPSC-CMs) for Post-infarction Cardiac Repair and Regeneration in Swine*.*

Animal research: All animal procedures were conducted in accordance with institutional guidelines and the Animal Research: Reporting of *In Vivo* Experiments (ARRIVE) guidelines. Animal research was approved by the Committee for Ethics in Animal Use (CEUA) of the University of São Paulo Medical School under license number 1089/2018, approved on September 3, 2018. All efforts were made to minimize animal suffering.

### Cell lines

Three *IRX3-*KO cell lines derived from two human iPSC (hiPSC) lines were utilized in this study. The SS109 lineage, developed in our laboratory [55], was first assessed in January 2020 to generate two mutant lines (*IRX3*^*cl1.1-/-*^ and *IRX3*^*cl1.2-/-*^). The AICS-0037–172 (TROPO-GFP) hiPSC line was obtained from the Coriell Institute (Camden, NJ, USA) in July 2019, first assessed for experimental work at our lab in November 2019 and used to generate the *IRX3*^*cl2-/-*^ line in January 2022. The use of this line follows the citation and distribution requirements outlined by the Allen Cell Collection [56].

All hiPSC lines were maintained in StemFlex medium (Gibco) at 37ºC and 5% CO_2_. Cells were passaged using Versene (1x) (Gibco) upon reaching 80−90% confluency and were regularly monitored for mycoplasma contamination. Following CM differentiation, cells were maintained in RPMI 1640 medium supplemented with B-27 with insulin and refreshed every 4 days. When necessary, cells were dissociated with Trypsin-EDTA 0.25%.

### hiPSC generation

To exclude protocol and cell line-specific responses, we evaluated the role of *IRX3* depletion on CM differentiation using two distinct differentiation protocols and two hiPSCs lines: SS109 and TROPO-GFP (monoallelic mEGFP Tagged TNNI1 WTC. SS109 clone was generated with lentivirus from skin fibroblasts of a healthy donor following the protocol of Somers et.al. and previously published in Muñoz JJAM, 2022 [55,57]. Both cell lines were characterized for the expression of pluripotent markers by immunofluorescence and flow cytometry, *in vivo* teratoma formation assay and no karyotypic abnormalities were observed (S1and S3 Figs).

### Animal research

All animal procedures were conducted in accordance with the Animal Research: Reporting of *In Vivo* Experiments (ARRIVE) guidelines and approved by the Committee for Ethics in Animal Use (CEUA) of the University of São Paulo Medical School under license number 1089/2018, approved on September 3, 2018. All efforts were made to minimize animal suffering.

Adult male BALB/c mice (2–3 months old) were used for the *in vivo* teratoma formation assay. Animals were maintained under standard temperature and lighting conditions with ad libitum access to food and water. Mice were anesthetized using 2% isoflurane, and upon adequate sedation, received subcutaneous injections on the back near the hind limb. Each injection contained 5 × 10⁵ hiPSCs in 50 μL, mixed in a 1:1 ratio with Geltrex. Mice were monitored weekly for teratoma formation. Four weeks after injection, animals were euthanized by isoflurane inhalation (4–5%) in a closed chamber until respiratory arrest, followed by intracardiac perfusion to ensure irreversible death. Teratomas were collected, fixed in 10% formalin, and processed for histological evaluation using hematoxylin and eosin staining.

## Generation of hiPSC *IRX3*-KO lines

### Plasmid transfection (*IRX3*^*cl1-/-*^)

gRNAs were designed to target exon 1 of *IRX3*, upstream of the DNA binding domain, using the CRISPOR website (https://crispor.gi.ucsc.edu/). To generate *IRX3*^*cl1.1-/-*^ and *IRX3*^*cl1.2-/-*^ (SS109 clones), 2.5 x 10^4^ hiPSCs were mixed with 4 µg sgRNA (MLM3636, Addgene) and 4 µg plasmid Cas9 (hCas9 Church, Addgene). The cell-plasmid mixtures were electroporated using the Neon transfection system (ThermoFisher Scientific) under the conditions of 1,400 V, 20 ms, and 1 pulse. Subsequently, the electroporated cells were seeded in a 6-well plate containing mTeSR (StemCell Technologies) supplemented with 10 μM ROCK Inhibitor Y-27632 (Sigma-Aldrich) for 24 hours. The next day, the medium was changed to remove Y-27632. On day 2, the hiPSC SS109 clone was dissociated into single cells and replated into individual 96-well plates to generate single-cell clones. After 10–14 days, individual colonies were passaged and screened by PCR and the selected clones had their DNA sequence confirmed by sequencing.

### sgRNA design, synthesis and in vitro assembly of Cas9-gRNAs *(IRX3*^*cl2-/-*^)

The target gene sequences were identified using the CRISPRscan algorithm (http://www.crisprscan.org). gRNAs were designed to target exon 1 of *IRX3*, upstream of the DNA binding domain, using the CRISPRScan website [58] (scores ranging from 15 to 25, with an average score of 18), with the human genome in the Homo sapiens version as the reference (see the description of gRNAs in the S3 Table). The designed gRNAs obtained an efficiency score predicted by the CRISPRScan website [58]. From CRISPRScan, we also predicted the potential off-target sites for the gRNAs, defined by the CFD score [59]. For the *IRX3* gene, zero off-targets and zero mismatches were predicted. For the DNA oligo, a target-specific CRISPR RNA (crRNA) and a trans-activating crRNA (tracrRNA) were designed, combined into a single transcript, with a PAM sequence (NGG) allowing Cas9 to initiate binding. The assembled oligos were synthesized and purified by HPLC by ThermoFisher Scientific company.

The gRNAs were *in vitro* synthesized using the GeneArt Precision gRNA Synthesis Kit (ThermoFisher Scientific) according to the provided protocol. In brief, the DNA template was first assembled with 25 μL of polymerase chain reaction (PCR) components, including 12.5 μL of Phusion High-Fidelity PCR Master Mix (2x), 1.0 μL of Tracr Fragment + T7 Primer Mix, 1.0 μL of forward and reverse primers (0.3 μM for each primer, S4 Table), and 10.5 μL of nuclease-free water. The PCR was performed at 98 °C for 10 s, followed by 32 cycles of denaturation at 98 °C for 5 s, annealing at 55 °C for 15 s, and a final extension at 72 °C for 1 minute.

*In vitro* transcription of the sgRNA was conducted by incubating with another 20 μL reaction component, including 8 μL of DNTP mix (100 mM of ATP, GTP, CTP, and UTP), 6 μL of the above-mentioned DNA template, 4 μL of TranscriptAid Reaction Buffer (5×), and 2 μL of TranscriptAid Enzyme Mix for 3 hours at 37 °C. Subsequently, the residual DNA template was digested with 1 μL of DNase I. The sgRNA was purified using the GeneArt gRNA Clean-up Kit, verified by gel electrophoresis and finally stored at −80 °C.

In brief, 8 x 10^4^ cells per well were seeded in 12-well plates. Three hours later, the RNP complexes were formed by combining 0.5 μg equimolar mixture of tracer and gRNAs with 2.5 μg Cas9 nuclease (gRNA targeting *IRX3* and Cas9 protein) using Lipofectamine™ CRISPRMAX™ Cas9 Transfection Reagent (Invitrogen, Thermo Fisher Scientific) with TrueCut™ Cas9 v2 (Invitrogen, Thermo Fisher Scientific) protein following the manufacturer’s recommendations of Lipofectamine CRISPRMAX and protocol of Xin Yu et.al. [60]. At 4 hours post-transfection, the media containing the transfection reagent was removed and replaced with fresh mTeSR (STEMCELL Technologies) supplemented with Y-27632 (Sigma-Aldrich) for 1 day. After additional 2 days, the cells were dissociated and plated at single-cell density into 96-well plates. Clones were screened by sequencing of amplicons spanning the target site of *IRX3* gene exon 1. The positive hiPSC clones were expanded and characterized (S1-2 and S4 Figs). See S3-4 Tables for a complete list of guides and primers.

### Generation of hiPSC subclones by manual colony picking

Clonal sublines were generated by manual colony picking following established protocols for pluripotent stem cell clonal expansion [61,62]. To facilitate clonal isolation, cells were seeded at low density to allow the formation of spatially distinct colonies derived from single cells. Emerging colonies were identified under an inverted microscope based on morphology and size and were manually picked using sterile pipette tips under a stereomicroscope. Each colony was transferred into an individual well of a Geltrex-coated 24-well plate containing pre-warmed hiPSC mTeSR medium (StemCell Technologies). To enhance survival after manipulation, the medium was supplemented with 10 µM ROCK inhibitor (Y-27632) during the initial 24-hour recovery period. Subclones were subsequently expanded stepwise into larger vessels until sufficient cell numbers were obtained for cryopreservation, genomic DNA extraction, and downstream analyses. Only subclones exhibiting typical hiPSC morphology and stable proliferation were selected.

### Genomic DNA extraction, PCR amplification, and Sanger sequencing

Genomic DNA was extracted from hiPSC subclones using the DNeasy Blood & Tissue Kit (Qiagen, Hilden, Germany) according to the manufacturer’s instructions. The genomic region encompassing the CRISPR–Cas9 target site at the *IRX3* locus was amplified by PCR using primers flanking the sgRNA recognition sequence (S4 Table). Reactions were carried out using a GoTaq® Flexi DNA Polymerase (Promega, USA). PCR products were verified by agarose gel electrophoresis and purified using either the QIAquick Gel Extration kit (Qiagen, Hilden, Germany) or ExoSAP-IT (Affymetrix, USA). Purified amplicons were subjected to bidirectional Sanger sequencing using the same primers employed for amplification. Sequencing reactions were performed using the BigDye® Terminator v3.1 Cycle Sequencing Kit (Applied Biosystems, USA) and analyzed on a 3500xL Genetic Analyzer (Applied Biosystems, USA). Sequencing chromatograms were inspected and analyzed using Geneious Prime (Biomatters, New Zealand) to confirm genotype.

### Genome Integrity Assessment by SNP array

To assess genomic stability following CRISPR–Cas9 editing, genome-wide SNP array analysis was performed using the Axiom™ Human Genotyping SARS-CoV-2 Research Array (~870k markers, Thermo Fisher Scientific) on the GeneTitan™ MC Instrument. Genomic DNA was extracted from parental wild-type cells (*IRX3*^*cl1+/+*^ and *IRX3*^*cl2+/+-*^) and their respective edited clones (*IRX3*^*cl1.1-/-*^, IRX*3*^*cl1.2-/-*^, and *IRX3*^*cl2-/-*^). DNA (200 ng per sample) was processed using the Axiom™ 2.0 Reagent Kit following the standard workflow, which includes amplification, fragmentation, precipitation, and hybridization to array plates, followed by automated washing, staining, and scanning. Genotype calling and quality control were performed using Axiom™ Analysis Suite v5.1 (Thermo Fisher Scientific). Single Nucleotide Variant (SNV) calling was executed using the standard Genotyping workflow, while Copy Number Variation (CNV) and Loss of Heterozygosity (LOH) were assessed using the CNV Discovery workflow, utilizing GRCh38/hg38 as the reference genome. To verify genomic integrity, SNV concordance analysis was performed by comparing genotype calls between parental and edited cell pairs. Concordance rates >98% between isogenic pairs were used as the threshold for genomic stability, while comparisons between genetically distinct backgrounds served as specificity controls.

### Flow cytometry

The hiPSCs were fixed with 4% paraformaldehyde (PFA, Sigma-Aldrich) at room temperature for 20 min, permeabilized with 0.1% Triton X-100 (Sigma-Aldrich) for 15 min at 4 ºC and incubated with blocking solution (3% BSA/PBS, Sigma-Aldrich) for 30 min at 4 ºC. Next, the cells were incubated with the primary antibodies (1:100) diluted in blocking solution for 1 hour at 4 ºC. The fluorescently conjugated antibodies used to characterize undifferentiated cells were Oct4 (Millipore), Sox2 (Millipore), TRA-1–60 (Millipore) and TRA-1–81 (Millipore). Samples were read on BD Accuri™ C6 Personal Flow Cytometry (BD Biosciences) and analyzed with FloJow™ v10.8.1 Software (BD Biosciences).

### Differentiation into hiPSC-CMs

The differentiation processes for *IRX3*^*cl1+/+*^*, IRX3*^*cl1.1-/-*^*, IRX3*^*cl1.2-/-*^*, IRX3*^*cl2+/+*^*, IRX3*^*cl2-/-*^ hiPSCs into CMs were conducted according to Wnt pathway stimulation and inhibition protocols adapted from previous studies. Two well-established CM differentiation protocols were utilized to reinforce the reproducibility of our findings using *IRX3*^*cl1*^ [63] and *IRX3*^*cl2*^ [64] clones. For both, a couple of days before the start of the protocol, cells were subcultured in the proportion of 1:3 and seeded into Geltrex (Gibco)-coated 12-well plates. Upon reaching 80–90% confluence, they were treated with CHIR99021 (6–12 μM, Millipore) and cultured in RPMI 1640 medium supplemented with B-27 minus insulin (Gibco). For *IRX3*^*cl1*^ clones, following a 24-hour 8 µM CHIR99021 treatment, the medium was replaced with RPMI 1640 supplemented with B-27 minus insulin and 10 ng/ml BMP4 (R&D Systems). On day 2, medium was changed to RPMI 1640 supplemented with B-27 minus insulin, 2.5 µM KY2111 (Cayman Chemical) and XAV939 (Cayman Chemical). Starting from day 4, the medium was refreshed every 48 hours with RPMI 1640 supplemented with B-27 with insulin (Gibco). For *IRX3*^*cl2*^ clones, 48 hours after CHIR00921 treatment, the medium was replaced with RPMI 1640 supplemented with B-27 minus insulin. On day 3, the medium was changed to RPMI 1640 supplemented with B-27 minus insulin and 2 µM Wnt-C59 (Tocris Bioscience) and incubated for 48 hours. On day 5, the medium was replaced again with RPMI 1640 supplemented with B-27 minus insulin. From day 7 onwards, cells were cultured in RPMI 1640 medium supplemented with B-27 with insulin until the end of protocol. Metabolic selection of hiPSC-CMs was conducted upon the observation of beating cells, between days 10 and 12. During this period, cells were cultured with glucose-free RPMI 1640 medium supplemented with B-27 with insulin (Gibco) for 48 hours. When necessary, the medium was renewed for an additional 2 days to ensure optimal purification of the CMs.

### RNA isolation, Microarray preparation and analysis

Total RNA was isolated using the RNeasy Micro Kit (Qiagen). The RNA samples for whole transcriptome expression analysis were enzymatically fragmented and biotinylated using GeneChip^TM^ Whole Transcript Expression Arrays (Applied Biosystems). The samples were then hybridized with the GeneChip^TM^ WT PLUS Reagent Kit (Applied Biosystems) and scanned using the GeneTitan™ Microarray System (Applied Biosystems). The entire procedure for sample processing, running, and scanning followed the manufacturer’s protocol. Raw expression data for the D4-15 *IRX3*^*cl1+/+*^sample was subjected to reanalysis from previously published data by our group (GSE188749) [55]. Expression levels of the *IRX3*^*cl1.1-/-*^ were assessed in the same microarray chip (GSE262776). Raw intensity values were background-corrected, normalized, and log2-transformed using the Robust Multi-array Average (RMA) algorithm. Differentially expressed genes (DEGs) were detected using limma R package [65,66]. Genes with logFC >= 0.5 or <= −0.5 and FDR < 0.01 were considered differentially expressed.

### RT-qPCR and relative gene expression analysis

Total cellular RNA was extracted with TRIzol^TM^ Reagent (Invitrogen, Thermo Fisher Scientific) followed by column purification with the RNeasy Micro kit (Qiagen), according to the manufacturer’s instructions. Reverse transcription was performed using 500 ng of total RNA with the SuperScript™ IV First-Strand Synthesis System (Invitrogen, Thermo Fisher Scientific). Quantitative real-time PCR was performed using TaqMan™ Universal PCR Master Mix (Invitrogen, Thermo Fisher Scientific) or QuantiTect SYBR Green PCR Kit (Qiagen). Relative gene expression was determined using the 2^−ΔΔCt^ method, with *GAPDH* or *ACTB* serving as endogenous controls (see S4 Table for primer sequences).

### Fluorescence microscopy

hiPSC-CMs were plated at 1 x 10^4^ cells/cm^2^ in 96-well fluorescence plates (Wells Greiner Microplate, Black, Clear Bottom), previously treated with Geltrex. Cells were fixed in 4% PFA, permeabilized with 0.5% Triton X-100/PBS and blocked with 5% BSA for 1 hour. Immunostaining was performed using anti-Troponin I cardiac (1:400, Hytest), anti-KI67 (1:200, Abcam), OCT4 (1:200, Cell Signaling), NANOG (1:200, Cell Signaling) and SOX2 (1:200, Cell Signaling), followed by appropriate incubation with secondary antibodies conjugated with Alexa Fluor 555/647 fluorophores (1:500, ThermoFisher Scientific). DAPI (1 mg/ml, ThermoFisher Scientific) was used to counterstain nuclei. For the high-content screening analysis, 16 up to 64 fields of view/well were collected using EVOS M7000 automated Imaging System (Invitrogen, Thermo Fisher Scientific) or IN Cell Analyzer 2000 (GE Healthcare) at either 20× or 40 × magnification.

After the acquisition, the raw images were analyzed using CellProfiler (4.2.0) [67]. The high-throughput analysis retrieved phenotypic information of more than 2000 single-cells per sample, for cell-based analysis, or 150–500 images for image-based analysis. Nuclear mean fluorescence intensity (MFI) of NKX2.5 and GATA4 were extracted from each individual nucleus within the images after background subtraction correction. For detecting replicating cells, 5 µM 5-ethynyl-20 deoxyuridine (EdU, ThermoFisher Scientific) was added to the cell medium 48 hours. Before incubation with fluorescent secondary antibodies, the cells were processed using the Click-iT™ EdU Alexa Fluor™ 647 HCS Assay (Invitrogen, Thermo Fisher Scientific) according to the manufacturer’s instructions. The EdU proliferative rate was calculated by counting the number of positive-staining cells divided by the total number of DAPI-positive cells multiplied by 100.

### Confocal microscopy

On day 27, hiPSC-CMs were plated to confluence at 3 x 10^4^ cells/cm^2^ in CellView Culture Slide (Greiner BioOne), previously treated with Geltrex. Cells were fixed with 4% PFA, permeabilized with 0.5% Triton X-100/PBS and blocked with 5% BSA for 1 hour. Immunostaining was performed using anti-TOM20 (1:200, Santa Cruz Biotechnology), Alpha-Actinin (Sarcomeric) (1:200, Sigma), GATA4 (1:200, Santa Cruz Biotechnology), NKX2–5 (1:200, Cell Signaling), Connexin 43 (1:200, Abcam), followed by incubation with appropriate secondary antibodies conjugated with Alexa Fluor 555/647 fluorophore (1:500, ThermoFisher Scientific). DAPI (1 mg/ml) was used to counterstain nuclei. Images were acquired on an Axio Observer.Z1 (Carl Zeiss) equipped with a Confocal Spinning Disk Unit (CSU-X1) (Yokogawa Life Science) and a Rolera EM-C2 EMCCD camera (Teledyne Photometrics). DAPI, TnI, and TOM20 were, respectively, detected by sequentially illuminating samples with 405 nm, 488 nm, and 561 nm laser lines through a C-Apochromat 40 × /1.20 W Korr objective (Carl Zeiss). Image analysis was performed in CellProfiler after preprocessing in Fiji Software (NIH) [68]. Briefly, “Subtract Background” filter plugin (rolling ball radius = 5) was applied to TOM20 images to reduce background and highlight mitochondria. Preprocessed TOM20 images were then processed in CellProfiler to extract mean TOM20 intensity and distribution in segmented mitochondria. For Connexin-43 quantification, CX43-positive and TnI-positive areas were segmented using the ‘Threshold’ module. The ratio of CX43 area to cardiomyocyte area was then calculated and multiplied by 100 (i.e., CX43 area / TnI-positive area × 100). The resulting values were converted to fold change to facilitate comparison across groups. Representative images were further processed by using the auto function of “Brightness/Contrast” plugin in Fiji, in order to highlight subcellular localization of proteins of interest. We measured the MFI of each protein of interest and, when applicable, cell area. Violin plots represent data from several hundreds of cells.

### Scrape loading dye transfer assay (SLDT)

We performed the dye transfer assay to assess intercellular communication through gap junctions. For this, the cells were seeded at 1 x 10^4^ density on a 96-well plate, previously treated with Geltrex. Cells were washed with Ca^2+^/Mg^2+^-PBS (Gibco) and incubated with a solution prepared in Ca^2+^/Mg^2+^-PBS containing Lucifer Yellow dye (1 mg/ml, Sigma-Aldrich), propidium iodide (10 μg/ml, Sigma-Aldrich) and Hoechst 33342 (50 μg/ml, Invitrogen). After addition, a cut was made with a stainless-steel blade of approximately 4 mm specially adapted in-house for this purpose. After 5–40 minutes of incubation, cells were washed again with Ca^2+^/Mg^2+^-PBS to remove background fluorescence, and subsequently fixed with 4% PFA solution in PBS. Then, the images centering the cut were acquired using the EVOS M7000 Imaging System and processed and analyzed using CellProfiler software.

### Morphology and contractile structures with MorphoScript

To characterize the hiPSC-CMs morphology as well as the organization of their contractile structures, hiPSC-CMs were dissociated with Trypsin-EDTA 0.25% (Gibco) for 5 min at 37 °C and replated on a 96-well plate with 10 μM ROCK Inhibitor Y-27632 added to the maintenance medium. Low confluence was used to obtain isolated cells (0.7 x 10^4^ cells/well). The cells were then fixed with 4% PFA for 15 min at room temperature, and permeabilized with 0.1% Triton X-100 for 5 min. The blocking was performed with 5% BSA for 60 min at room temperature. Alpha-Actinin (Sarcomeric) (1:200, Sigma-Aldrich) diluted in 2% BSA was used and incubated overnight at 4 °C. After incubation with primary antibody, cells were incubated with appropriate secondary antibody conjugated with Alexa Fluor 555 fluorophore (1:500, ThermoFisher Scientific) and DAPI (1 mg/ml) for 1 hour at room temperature.

The images were acquired using the EVOS M7000 Imaging System at × 40 magnification and analyzed using the MorphoScript Software in MATLAB, as previously described [69]. The isolated cells at the periphery were segmented manually. For the analysis, we set the window size to 15 so each window contains at least five contractile structures labeled with alpha-actinin. For the rest of the parameters, the default values were kept. This analysis allowed us to describe the morphology of hiPSC-CMs through the measurement of the sarcomeric organization (the order), spacing, alignment (the dispersion) and geometric parameters such as the cell surface area.

### Contractile properties measurement

For the measurement of contractile properties, hiPSC-CMs were plated in 96-well plates and allowed to beat spontaneously. Recordings were made using the EVOS M7000 Imaging System equipped with a thermostatic chamber (5% CO_2_, 37 °C) and 20 × objective. Before any recordings, the cells were allowed to stabilize in the chamber for at least 15 minutes. Spontaneously beating hiPSC-CMs were recorded for 15–20 seconds per field of view, with the imaging system set to a capture rate of 30 frames per second. Contractility analysis was conducted using ContractionWave Software, an open-source tool designed for large-scale analysis of CM contraction properties [70]. The recorded videos were imported into the Software, and parameters related to time, speed, area and frequency of contraction were extracted as previously described [70].

### Calcium transient measurement

We employed two different calcium-sensitive fluorescent dyes to analyze calcium transients. For the single-wavelength calcium indicator method, *IRX3*^*cl2+/+*^ and *IRX3*^*cl2-/-*^ hiPSC-CMs were seeded at a density of 3 x 10^4^ cells on a 96-well plate. Five days after plating, cells were loaded with the Fluo-4 NW Calcium Assay Kit (Molecular Probes) for 45 min at 37 °C. Following incubation, cells were washed and underwent a 5-minute de-esterification process with Tyrode’s solution (NaCl [135 mM], KCl [5.4 mM], MgCl [1.0 mM], HEPES [10 mM], CaCl_2_ [1.8 mM], glucose [5.0 mM] [pH 7.4]). Fluorescent signals were recorded from the center of the plates using the EVOS M7000 Imaging System. Excitation was performed at 488 nm and emission fluorescence signals were collected at 516 nm for a duration of 20 s.

The recorded frames were imported into Fiji Software for analysis. After applying filtering, calcium transients from fluorescence signals were obtained. Calcium transients for each sample were then blinded and imported into pClamp software (version 11.2, Molecular Devices) for further analysis. Following baseline correction, the period containing each individual calcium transient was identified in pClamp software. The software’s built-in algorithms were used to calculate the parameters. The amplitude of the calcium transient was measured as the difference between the peak fluorescence intensity and the baseline fluorescence intensity. The time of decay was calculated by fitting an exponential function to the decay phase of the signal for 50% and 90% decay. To account for variations in spontaneous beating rates, calcium transient durations (CaD_50_ and CaD_90_) were normalized using Fridericia’s formula (CaDc = CaD / RR^1/3^, where RR is the transient interval). The maximum rise slope was determined as the highest value of the first derivative during the rise phase. The average value of each parameter for each sample was then calculated, and the results were plotted and compared between the two groups.

For the dual-wavelength measurement, *IRX3*^*cl1+/+*^ and *IRX3*^*cl1-/-*^ hiPSC-CMs were plated on laminin-coated glass coverslips. Seven days after plating, the cells were loaded with Fura-2, AM (ThermoFisher Scientific) diluted in Tyrode’s free Ca^2+^ solution (final concentration of 2 μM) and incubated for 10 min at room temperature. Next, cells were incubated for an additional 5 min at 37 °C and washed once with Tyrode free Ca^2+^ to exclude the contribution of trans-sarcolemma Ca^2+^ influx via voltage gated Ca^2+^ channels. Prior to measurements, cells were incubated for 5 min to enable complete de-esterification of intracellular Fura-2. Intracellular Ca^2+^ events were recorded using a 40 × objective on an Olympus IX70 microscope fitted with an IonOptix system (Olympus; IonOptix) at 37 °C. Cells were alternately excited at 340 nm and 380 nm, and the emitted fluorescence was collected at 510 nm. The 340/380 nm fluorescence ratio was used to quantify intracellular calcium levels. The recorded fluorescence ratios were analyzed using IonWizard software to determine calcium transient parameters, including amplitude, calcium decay and max slope for each sample.

### Membrane potential

Electrical activity was recorded using the FluoVolt™ Membrane Potential Kit (Molecular Probes). Fluovolt dye was diluted in Tyrode’s solution pre-warmed to 37 °C containing PowerLoad (a solubilizing agent provided in the kit) and Blebbistatin (100 μM, Sigma-Aldrich) to prevent contraction artifacts. Cells were incubated with the solution for 10 min at room temperature, followed by an additional 5 min at 37 °C. After incubation, the cells were washed once with Tyrode’s solution to remove excess dye. Fluorescence intensities were measured using a multi-mode microplate reader (SpectraMax iD3, Molecular Devices) with excitation and emission wavelengths set at 480 nm and 540 nm, respectively. To capture electrical signals with maximal time resolution, a minimal sampling interval of 10 ms was used. All experiments were conducted at a controlled temperature of 37 °C to maintain physiological conditions. The acquired fluorescence signals were analyzed using pClamp software (version 11.2, Molecular Devices) to determine the average action potential duration for each sample.

### Single-cell ATAC-seq and ChIP-seq (*in silico*) analysis

scATAC-seq data (GEO ID: GSE181346) [71] and ChIP-seq data (GEO ID: GSE159411) [72] were obtained from the Gene Expression Omnibus (GEO) database. The scATAC-seq samples consisted of CMs derived from induced pluripotent stem cells (iPSCs) after 5 days of differentiation. ChIP-seq samples were collected after 6 days of induced differentiation from iPSCs to CMs, targeting GATA4, NKX2–5, and TBX5 transcription factors. scATAC-seq profiles were aggregated across cells and analyzed as pseudo-bulk data: using the preprocessed BED files to identify open chromatin genomic regions, which were further annotated using the ChIPseeker package in R [73]. Promoter regions were defined as ±1 kb around the transcription start site (TSS) of the nearest gene. This pseudo-bulk strategy for scATAC-seq peak calling and annotation is similar to approaches used previously to derive consensus peak sets from aggregated scATAC-seq datasets [74]. Next, IRX3 motifs within scATAC-seq data were identified using motifs obtained from the “encode_pwms” object in the chromVARmotifs package [75]. Specifically, we utilized motifs derived from [76] (core sequence: ACATGT) and [77] (core sequence: ACATGA) to capture both canonical and variant binding specificities (visualized in S7 Fig). Overlapping genomic regions between scATAC-seq peaks and predicted IRX3 motifs were calculated using the matchMotifs function from the motifmatchr package [75]. The output was processed with the “reduce” function from the IRanges package [78] to merge overlapping or adjacent genomic regions.

After identifying IRX3 motifs, ChIP-seq data analysis was performed. For each ChIP target (GATA4, NKX2–5, TBX5), overlapping peaks from downloaded BED or BB files were merged using the ChIPseeker package [73]. The data, initially in human genome version hg19, was lifted over to hg38 using the liftOver function from the rtracklayer package [73,78]. The resulting peaks were annotated. To identify genomic regions with open chromatin and IRX3 motifs for each ChIP target, we used the function findOverlapOfPeaks from the package ChIPpeakAnno [79]. The inputs included the ChiP targets positive regions and the scATAC-seq + IRX3 Motif genomic intervals. This analysis takes multiple sets of genomic intervals (peaks) as input and computes their overlaps, returning both pairwise and higher-order intersections. Using the default overlap criteria (minimum 1 bp overlap within the same genome build), findOverlapsOfPeaks extracted the subset of genomic regions where all four peak sets intersected, which we defined as co-localized regions of open chromatin, IRX3 motif presence, and TF binding. These co-localized regions were then carried forward for gene assignment and downstream ontology enrichment analyses.

We repeated this process for NKX2–5 and TBX5 ChIP data. The findOverlapOfPeaks function was then applied again, with the overlapping regions from the previous analysis as input. The resulting overlapping regions were then extracted and annotated. For data visualization, Venn diagrams were generated using the gplot package to illustrate the overlap between different sets of genomic regions. Lastly, Gene Ontology enrichment analysis was performed using the clusterProfiler package [80]. Genes annotated for each transcription factor, the intersection between them, and all targets were selected for Biological Process and Molecular Function enrichment analyses. The OrgDb parameter was set ‌‌for both analyses to “org.Hs.e.g.,db.”

### Transmission electron microscopy

Cells were grown to 90% confluence before being washed, trypsinized, pelleted, and fixed in 2.5% glutaraldehyde in 0.1 M sodium cacodylate buffer (pH 7.2) overnight. Samples were then post fixed in 1% osmium tetroxide for 2 h, stained in bloc with 0.5% uranyl acetate, rinsed, and dehydrated in graded ethanol. After immersion in propylene oxide, samples were embedded in epoxy resin (Spurr, Electron Microscopy Sciences, EMS, Hatfield PA, USA) and polymerized for 42 hours at 75 °C. Ultrathin sections were stained with lead citrate and uranyl acetate and TEM images were collected with a JEM-1400 Plus microscope (JEOL, Japan) applying tension of 120 kV and using camera OneView 4K x 4K (Gatan, USA). Mitochondria segmentation was manually performed in Fiji software by an observer who was blind to the experimental conditions.

### Statistical analysis

Data were derived from 3–6 independent replicates and are presented as means ± standard deviation (SD), box plot showing the median, interquartile range (25^th^–75^th^ percentiles), or violin plot ranging from the 1st through the 3rd quantile. One-way ANOVA followed by Tukey’s multiple comparisons test was used to analyze data from Fig 1D-E and Fig 4A-B; two-way repeated measures ANOVA with Sidak’s correction for multiple comparisons was used for Figs 2L and 3D; and the Mann-Whitney test was used for Fig 2B (“Order” plot). Student’s *t*-test was applied for the remaining figures (Figs 1-5 and S1-S5 Figs), unless otherwise stated. All *t*-tests were two-tailed and paired. Statistical significance was considered achieved when p < 0.05. Further statistical details are provided in the figure legends.

### Resource availability

Requests for further information and resources should be directed to and will be fulfilled by the lead contact, Jose Eduardo Krieger (j.krieger@hc.fm.usp.br).

### Materials availability

This study did not generate new unique reagents.

## Supporting information

S1 Fig *IRX3* mutant allele description and karyotype. **(A-C)** Allele description of (A) *IRX3*^*cl1.1-/-*^, (B) *IRX3*^*cl1.2-/-*^, and (C) *IRX3*^*cl2-/-*^ mutant hiPSC clones. (**D)** Sequencing alignment of *IRX3*-KO hiPSC subclones. gRNA + PAM sequences are shown in green. **(E)** Relative mRNA levels of *IRX3* in *IRX3*-KO hiPSC clones at day 4 of differentiation (n = 3 independent replicates). (**F-G**) Representative images of karyotype analysis of (F) *IRX3*^*cl1.1-/-*^, *IRX3*^*cl1.2-/-*^, and *IRX3*^*cl2-/-*^ hiPSCs and (G) of *IRX3*^*cl2+/+*^ and *IRX3*^*cl2-/*-^. Data are presented as box plots with individual replicates. Student’s t-test. *P < 0.05; ***P < 0.001 vs *IRX3*^*cl1+/+*^.
(PDF)

S2 Fig Protein alignment of encoded peptides from *IRX3* mutant alleles. Protein alignment for human IRX3 against predicted encoded peptides of *IRX3* mutant alleles. Transheterozygous alleles are indicated by allele number (1 and 2). *IRX3*^*cl1.2*^ allele 2 is a 227 bp deletion encompassing the *IRX3* transcription start site (TSS), indicated by the red *. A potential alternative TSS is indicated by the black arrowhead.
(PDF)

S3 Fig Pluripotency characterization for *IRX3*-KO hiPSC clones. **(A)** Representative immunofluorescence of the pluripotency markers OCT4, NANOG and SOX2 in *IRX3*-KO hiPSC clones. Images showing morphology, self-aggregation and embryoid body (EB) formation on phase-contrast microscopy. **(B)** Representative flow cytometry of pluripotency markers SOX2, OCT4, TRA1–81 and TRA1–60 in *IRX3*-KO hiPSC clones. **(C)** Teratoma formation assay. Representative images show gross morphology (left column) and low-magnification H&E staining of whole tumor sections (second column; scale bar: 2 mm). Colored boxes (scale bar: 100 µm) highlight organized tissues confirming differentiation into all three germ layers: Ectoderm (blue; neuroepithelium and epithelial cysts), Mesoderm (yellow; skeletal muscle and connective tissue), and Endoderm (red; gut-like epithelium and glandular epithelium).
(PDF)

S4 Fig *IRX3*-KO cardiomyocytes display increased expression of working cardiomyocyte genes. **(A-D)** Relative mRNA levels of (A) *TNNI1*, (B) *GJA1*, (C) *GJA5,* and (D) *SCN5A* genes (n = 3–4 independent replicates) from *IRX3*^*cl1+/+*^ and *IRX3*^*cl1.1-/-*^ hiPSC-CMs at day 15 of differentiation. Data are presented as box plots with individual replicates. Student’s t-test. *P < 0.05 vs *IRX3*^*cl1+/+*^.
(PDF)

S5 Fig *IRX3* depletion enhances electromechanical profile and functional coupling. **(A-E)** Relative mRNA levels of (A) *MYH6*, (B) *MYH7*, (C) *SERCA2*, (D) *RYR2*, and (E) *TNNI3* in *IRX3*^*cl2-/-*^ hiPSC-CMs at day 30 of differentiation (n = 3–4 independent replicates). **(F)** Schematic figure of analyzed field of view of hiPSC-CMs self-arranged in a monolayer syncytium-like form. **(G)** Action potential duration at 50% (APD50) and 90% (APD90) of repolarization and **(H)** Action potential amplitude (n = 4 independent replicates). **(I)** Representative curves of action potentials. **(J)** Representative immunofluorescence of scrape loading dye transfer assay (SLDT) – living cells were stained with Lucifer yellow dye (green), dead cells were stained with Propidium iodide (red), and cell nuclei were counterstained with Hoechst 33342 (blue). **(K)** Quantification of the dye transfer area (n = 3 independent replicates). Scale bars = 20 µm. Student’s t-test. Data are presented as box plots with individual replicates. *P < 0.05; **P < 0.01; ****P < 0.0001 vs *IRX3*^cl2+/+^.
(PDF)

S6 Fig Early expression profiles of cardiac transcription factor genes in *IRX3*-KO cells. (A-I) Relative mRNA levels of *TBX5*, *GATA4*, and *NKX2–5* in (A-C) *IRX3*^*cl1.1-/-*^, (D-F) *IRX3*^*cl1.2-/-*^, and (G-I) *IRX3*^*cl2-/-*^ cells, respectively (n = 4–10 independent replicates). Data are presented as box plots with individual replicates. Student’s t-test. *P < 0.05 vs *IRX3*^*cl1+/+*^ or *IRX3*^*cl2+/+*^.
(PDF)

S7 Fig Consensus DNA binding motifs of IRX3. (A) Sequence logos illustrating the binding specificity of IRX3 across independent datasets. (Top/Middle) Motifs identified by Berger et al. [76] characterized by a conserved ACATGT core sequence. (Bottom) The IRX3 motif from the Jolma et al. dataset [77], exhibiting a variant ACATGA core. The height of each letter (y-axis) represents the information content (bits) and the relative frequency of the nucleotide at that specific position (x-axis), indicating the strictness of the binding preference.
(PDF)

S8 Fig Genome-wide co-occupancy analysis of IRX3 and core cardiac transcription factors. (A) UpSet plot visualizing the intersection of IRX3 target genes with NKX2–5, GATA4, and TBX5 binding profiles. Vertical bars represent the size of each unique intersection (co-occupied gene sets), while horizontal bars (left) show the total number of IRX3 targets overlapping with each individual transcription factor. The matrix diagram indicates the specific combination of factors present at these loci. (B) Distribution of gene biotypes (Protein-Coding vs. Non-Coding) across the ‘Co-Occupied’ and ‘IRX3-Solo’ target gene populations. (C-F) GO enrichment analysis of biological processes. Analysis was performed on: (C) the ‘IRX3-solo’ gene population; (D) the aggregate union of genes co-occupied by IRX3 and at least one cardiac TF; (E) the core intersection of genes co-occupied by IRX3 and all three cardiac TFs simultaneously; and (F) specific target subsets defined by IRX3 co-binding with GATA4, NKX2–5, or TBX5 individually (see S2 Table for the complete data set).
(PDF)

S9 Fig Overlap of IRX3 motifs, scATAC-seq peaks, and Transcription Factor (TF) binding sites across genomic regions. The percentage of shared peaks is shown on the x-axis, representing the proportion of scATAC-seq peaks that contain an IRX3 motif and overlap with binding sites for the indicated TFs (y-axis). Colors indicate the specific genomic region where the overlap occurs. The data highlight that the majority of significant overlaps fall within promoter regions, suggesting a critical role for IRX3 and these TFs in gene regulation.
(PDF)

S10 Fig Molecular functions of enriched pathways of cTFs and IRX3 target genes. (A-C) GO terms associated with molecular functions for (A) all genes encountered, (B) common genes across ChIP-seq targets and IRX3 motifs, and (C) genes with IRX3 motifs and ChIP-seq peaks for either GATA4, NKX2–5, or TBX5.
(PDF)

S1 Table Genomic Integrity, Quality Control, and Isogenicity Analysis. (XLSX)

S2 Table List of ChIP-seq target genes. (XLSX)

S3 Table List of guides. (XLSX)

S4 Table List of primers for DNA sequencing of CRISPR–Cas9-edited hiPSCs and qPCR. (XLSX)

S5 Table List of Reagents or Resources. (XLSX)

## References

[1] Oikonomopoulos A, Kitani T, Wu JC. Pluripotent Stem Cell-Derived Cardiomyocytes as a Platform for Cell Therapy Applications: Progress and Hurdles for Clinical Translation. Mol Ther. 2018;26(7):1624–34. doi: 10.1016/j.ymthe.2018.02.026 29699941 PMC6035734

[2] Crestani T, Steichen C, Neri E, Rodrigues M, Fonseca-Alaniz MH, Ormrod B, et al. Electrical stimulation applied during differentiation drives the hiPSC-CMs towards a mature cardiac conduction-like cells. Biochem Biophys Res Commun. 2020;533(3):376–82. doi: 10.1016/j.bbrc.2020.09.021 32962862

[3] Karakikes I, Ameen M, Termglinchan V, Wu JC. Human induced pluripotent stem cell-derived cardiomyocytes: insights into molecular, cellular, and functional phenotypes. Circ Res. 2015;117(1):80–8. doi: 10.1161/CIRCRESAHA.117.305365 26089365 PMC4546707

[4] Karbassi E, Murry CE. Flexing Their Muscles: Maturation of Stem Cell-Derived Cardiomyocytes on Elastomeric Substrates to Enhance Cardiac Repair. Circulation. 2022;145(18):1427–30. doi: 10.1161/CIRCULATIONAHA.122.059079 35500046 PMC9069846

[5] Liu Y-W, Chen B, Yang X, Fugate JA, Kalucki FA, Futakuchi-Tsuchida A, et al. Human embryonic stem cell-derived cardiomyocytes restore function in infarcted hearts of non-human primates. Nat Biotechnol. 2018;36(7):597–605. doi: 10.1038/nbt.4162 29969440 PMC6329375

[6] Ottaviani D, Ter Huurne M, Elliott DA, Bellin M, Mummery CL. Maturing differentiated human pluripotent stem cells in vitro: methods and challenges. Development. 2023;150(11):dev201103. doi: 10.1242/dev.201103 37260361

[7] Hofbauer P, Jahnel SM, Papai N, Giesshammer M, Deyett A, Schmidt C, et al. Cardioids reveal self-organizing principles of human cardiogenesis. Cell. 2021;184(12):3299-3317.e22. doi: 10.1016/j.cell.2021.04.034 34019794

[8] Chong JJH, Yang X, Don CW, Minami E, Liu Y-W, Weyers JJ, et al. Human embryonic-stem-cell-derived cardiomyocytes regenerate non-human primate hearts. Nature. 2014;510(7504):273–7. doi: 10.1038/nature13233 24776797 PMC4154594

[9] Shiba Y, Gomibuchi T, Seto T, Wada Y, Ichimura H, Tanaka Y, et al. Allogeneic transplantation of iPS cell-derived cardiomyocytes regenerates primate hearts. Nature. 2016;538(7625):388–91. doi: 10.1038/nature19815 27723741

[10] Marchiano S, Nakamura K, Reinecke H, Neidig L, Lai M, Kadota S, et al. Gene editing to prevent ventricular arrhythmias associated with cardiomyocyte cell therapy. Cell Stem Cell. 2023;30(4):396-414.e9. doi: 10.1016/j.stem.2023.03.010 37028405 PMC10283080

[11] Ribeiro da Silva A, Neri EA, Turaça LT, Dariolli R, Fonseca-Alaniz MH, Santos-Miranda A, et al. NOTCH1 is critical for fibroblast-mediated induction of cardiomyocyte specialization into ventricular conduction system-like cells in vitro. Sci Rep. 2020;10(1):16163. doi: 10.1038/s41598-020-73159-0 32999360 PMC7527973

[12] Linhares VLF, Almeida NAS, Menezes DC, Elliott DA, Lai D, Beyer EC, et al. Transcriptional regulation of the murine Connexin40 promoter by cardiac factors Nkx2-5, GATA4 and Tbx5. Cardiovasc Res. 2004;64(3):402–11. doi: 10.1016/j.cardiores.2004.09.021 15537493 PMC3252638

[13] Olson EN. Gene regulatory networks in the evolution and development of the heart. Science. 2006;313(5795):1922–7. doi: 10.1126/science.1132292 17008524 PMC4459601

[14] Cui M, Wang Z, Bassel-Duby R, Olson EN. Genetic and epigenetic regulation of cardiomyocytes in development, regeneration and disease. Development. 2018;145(24):dev171983. doi: 10.1242/dev.171983 30573475 PMC6307883

[15] George RM, Firulli AB. Hand Factors in Cardiac Development. Anat Rec (Hoboken). 2019;302(1):101–7. doi: 10.1002/ar.23910 30288953 PMC6312500

[16] Lints TJ, Parsons LM, Hartley L, Lyons I, Harvey RP. Nkx-2.5: a novel murine homeobox gene expressed in early heart progenitor cells and their myogenic descendants. Development. 1993;119(2):419–31. doi: 10.1242/dev.119.2.419 7904557

[17] Luna-Zurita L, Stirnimann CU, Glatt S, Kaynak BL, Thomas S, Baudin F, et al. Complex interdependence regulates heterotypic transcription factor distribution and coordinates cardiogenesis. Cell. 2016;164. doi: 10.1016/j.cell.2016.01.004 PMC4769693 26875865

[18] McFadden DG, Barbosa AC, Richardson JA, Schneider MD, Srivastava D, Olson EN. The Hand1 and Hand2 transcription factors regulate expansion of the embryonic cardiac ventricles in a gene dosage-dependent manner. Development. 2005;132(1):189–201. doi: 10.1242/dev.01562 15576406

[19] Riley P, Anson-Cartwright L, Cross JC. The Hand1 bHLH transcription factor is essential for placentation and cardiac morphogenesis. Nat Genet. 1998;18(3):271–5. doi: 10.1038/ng0398-271 9500551

[20] Christoffels VM, Keijser AG, Houweling AC, Clout DE, Moorman AF. Patterning the embryonic heart: identification of five mouse Iroquois homeobox genes in the developing heart. Dev Biol. 2000;224(2):263–74. doi: 10.1006/dbio.2000.9801 10926765

[21] Costantini DL, Arruda EP, Agarwal P, Kim K-H, Zhu Y, Zhu W, et al. The homeodomain transcription factor Irx5 establishes the mouse cardiac ventricular repolarization gradient. Cell. 2005;123(2):347–58. doi: 10.1016/j.cell.2005.08.004 16239150 PMC1480411

[22] Gaborit N, Sakuma R, Wylie JN, Kim K-H, Zhang S-S, Hui C-C, et al. Cooperative and antagonistic roles for Irx3 and Irx5 in cardiac morphogenesis and postnatal physiology. Development. 2012;139(21):4007–19. doi: 10.1242/dev.081703 22992950 PMC3472592

[23] He W, Jia Y, Takimoto K. Interaction between transcription factors Iroquois proteins 4 and 5 controls cardiac potassium channel Kv4.2 gene transcription. Cardiovasc Res. 2009;81(1):64–71. doi: 10.1093/cvr/cvn259 18815185 PMC2721642

[24] Kim K-H, Rosen A, Hussein SMI, Puviindran V, Korogyi AS, Chiarello C, et al. Irx3 is required for postnatal maturation of the mouse ventricular conduction system. Sci Rep. 2016;6:19197. doi: 10.1038/srep19197 26786475 PMC4726432

[25] Koizumi A, Sasano T, Kimura W, Miyamoto Y, Aiba T, Ishikawa T, et al. Genetic defects in a His-Purkinje system transcription factor, IRX3, cause lethal cardiac arrhythmias. Eur Heart J. 2016;37(18):1469–75. doi: 10.1093/eurheartj/ehv449 26429810 PMC4914882

[26] Zhang S-S, Kim K-H, Rosen A, Smyth JW, Sakuma R, Delgado-Olguín P, et al. Iroquois homeobox gene 3 establishes fast conduction in the cardiac His-Purkinje network. Proc Natl Acad Sci U S A. 2011;108(33):13576–81. doi: 10.1073/pnas.1106911108 21825130 PMC3158173

[27] Canac R, Cimarosti B, Girardeau A, Forest V, Olchesqui P, Poschmann J. Deciphering transcriptional networks during human cardiac development. Cells. 2022;11. doi: 10.3390/cells11233915 PMC9739390 36497174

[28] Garg V, Kathiriya IS, Barnes R, Schluterman MK, King IN, Butler CA, et al. GATA4 mutations cause human congenital heart defects and reveal an interaction with TBX5. Nature. 2003;424(6947):443–7. doi: 10.1038/nature01827 12845333

[29] Stennard FA, Costa MW, Elliott DA, Rankin S, Haast SJP, Lai D, et al. Cardiac T-box factor Tbx20 directly interacts with Nkx2-5, GATA4, and GATA5 in regulation of gene expression in the developing heart. Dev Biol. 2003;262(2):206–24. doi: 10.1016/s0012-1606(03)00385-3 14550786

[30] Kosicki M, Tomberg K, Bradley A. Repair of double-strand breaks induced by CRISPR-Cas9 leads to large deletions and complex rearrangements. Nat Biotechnol. 2018;36(8):765–71. doi: 10.1038/nbt.4192 30010673 PMC6390938

[31] Gourdie RG, Severs NJ, Green CR, Rothery S, Germroth P, Thompson RP. The spatial distribution and relative abundance of gap-junctional connexin40 and connexin43 correlate to functional properties of components of the cardiac atrioventricular conduction system. J Cell Sci. 1993;105 (Pt 4):985–91. doi: 10.1242/jcs.105.4.985 8227219

[32] Severs NJ, Bruce AF, Dupont E, Rothery S. Remodelling of gap junctions and connexin expression in diseased myocardium. Cardiovasc Res. 2008;80(1):9–19. doi: 10.1093/cvr/cvn133 18519446 PMC2533424

[33] Campostrini G, Kosmidis G, Ward-van Oostwaard D, Davis RP, Yiangou L, Ottaviani D, et al. Maturation of hiPSC-derived cardiomyocytes promotes adult alternative splicing of SCN5A and reveals changes in sodium current associated with cardiac arrhythmia. Cardiovasc Res. 2023;119(1):167–82. doi: 10.1093/cvr/cvac059 35394010 PMC10022870

[34] Wilde AAM, Amin AS. Clinical Spectrum of SCN5A Mutations. JACC: Clinical Electrophysiology. 2018;4(5):569–79. doi: 10.1016/j.jacep.2018.03.006 29798782

[35] Gwathmey JK, Hajjar RJ. Intracellular calcium related to force development in twitch contraction of mammalian myocardium. Cell Calcium. 1990;11(8):531–8. doi: 10.1016/0143-4160(90)90029-t 2148283

[36] Bers DM. Cardiac excitation-contraction coupling. Nature. 2002;415(6868):198–205. doi: 10.1038/415198a 11805843

[37] Bilioni A, Craig G, Hill C, McNeill H. Iroquois transcription factors recognize a unique motif to mediate transcriptional repression in vivo. Proc Natl Acad Sci U S A. 2005;102(41):14671–6. doi: 10.1073/pnas.0502480102 16203991 PMC1239941

[38] Tzahor E. Wnt/beta-catenin signaling and cardiogenesis: timing does matter. Dev Cell. 2007;13(1):10–3. doi: 10.1016/j.devcel.2007.06.006 17609106

[39] Ueno S, Weidinger G, Osugi T, Kohn AD, Golob JL, Pabon L, et al. Biphasic role for Wnt/beta-catenin signaling in cardiac specification in zebrafish and embryonic stem cells. Proc Natl Acad Sci U S A. 2007;104(23):9685–90. doi: 10.1073/pnas.0702859104 17522258 PMC1876428

[40] Black BL, Cripps RM. Myocyte Enhancer Factor 2 Transcription Factors in Heart Development and Disease. Heart Development and Regeneration. Elsevier. 2010. p. 673–99. doi: 10.1016/b978-0-12-381332-9.00030-x

[41] Greulich F, Rudat C, Kispert A. Mechanisms of T-box gene function in the developing heart. Cardiovasc Res. 2011;91(2):212–22. doi: 10.1093/cvr/cvr112 21498422

[42] Okubo C, Narita M, Inagaki A, Nishikawa M, Hotta A, Yamanaka S, et al. Expression dynamics of HAND1/2 in in vitro human cardiomyocyte differentiation. Stem Cell Reports. 2021;16. doi: 10.1016/j.stemcr.2021.06.014 PMC8365100 34297940

[43] Yamagishi H, Yamagishi C, Nakagawa O, Harvey RP, Olson EN, Srivastava D. The combinatorial activities of Nkx2.5 and dHAND are essential for cardiac ventricle formation. Dev Biol. 2001;239(2):190–203. doi: 10.1006/dbio.2001.0417 11784028

[44] Gordon JW. Regulation of cardiac myocyte cell death and differentiation by myocardin. Mol Cell Biochem. 2018;437(1–2):119–31. doi: 10.1007/s11010-017-3100-3 28631251

[45] Kattman SJ, Huber TL, Keller GM. Multipotent flk-1+ cardiovascular progenitor cells give rise to the cardiomyocyte, endothelial, and vascular smooth muscle lineages. Dev Cell. 2006;11(5):723–32. doi: 10.1016/j.devcel.2006.10.002 17084363

[46] Ahmed RE, Tokuyama T, Anzai T, Chanthra N, Uosaki H. Sarcomere maturation: function acquisition, molecular mechanism, and interplay with other organelles. Philos Trans R Soc Lond B Biol Sci. 2022;377(1864):20210325. doi: 10.1098/rstb.2021.0325 36189811 PMC9527934

[47] Amini H, Rezaie J, Vosoughi A, Rahbarghazi R, Nouri M. Cardiac progenitor cells application in cardiovascular disease. J Cardiovasc Thorac Res. 2017;9(3):127–32. doi: 10.15171/jcvtr.2017.22 29118944 PMC5670333

[48] Kwon C, Arnold J, Hsiao EC, Taketo MM, Conklin BR, Srivastava D. Canonical Wnt signaling is a positive regulator of mammalian cardiac progenitors. Proc Natl Acad Sci U S A. 2007;104(26):10894–9. doi: 10.1073/pnas.0704044104 17576928 PMC1904134

[49] Al Sayed ZR, Canac R, Cimarosti B, Bonnard C, Gourraud J-B, Hamamy H, et al. Human model of IRX5 mutations reveals key role for this transcription factor in ventricular conduction. Cardiovasc Res. 2021;117(9):2092–107. doi: 10.1093/cvr/cvaa259 32898233

[50] Katti P, Hall AS, Parry HA, Ajayi PT, Kim Y, Willingham TB, et al. Mitochondrial network configuration influences sarcomere and myosin filament structure in striated muscles. Nat Commun. 2022;13(1):6058. doi: 10.1038/s41467-022-33678-y 36229433 PMC9561657

[51] Karbassi E, Fenix A, Marchiano S, Muraoka N, Nakamura K, Yang X, et al. Cardiomyocyte maturation: advances in knowledge and implications for regenerative medicine. Nat Rev Cardiol. 2020;17(6):341–59. doi: 10.1038/s41569-019-0331-x 32015528 PMC7239749

[52] Feyen DAM, McKeithan WL, Bruyneel AAN, Spiering S, Hörmann L, Ulmer B. Metabolic Maturation Media Improve Physiological Function of Human iPSC-Derived Cardiomyocytes. Cell Reports. 2020;32. doi: 10.1016/j.celrep.2020.107925 PMC7437654 32697997

[53] Dai D-F, Danoviz ME, Wiczer B, Laflamme MA, Tian R. Mitochondrial Maturation in Human Pluripotent Stem Cell Derived Cardiomyocytes. Stem Cells Int. 2017;2017:5153625. doi: 10.1155/2017/5153625 28421116 PMC5380852

[54] Guo Y, Pu WT. Cardiomyocyte Maturation: New Phase in Development. Circ Res. 2020;126(8):1086–106. doi: 10.1161/CIRCRESAHA.119.315862 32271675 PMC7199445

[55] Muñoz JJAM, Dariolli R, da Silva CM, Neri EA, Valadão IC, Turaça LT, et al. Time-regulated transcripts with the potential to modulate human pluripotent stem cell-derived cardiomyocyte differentiation. Stem Cell Res Ther. 2022;13(1):437. doi: 10.1186/s13287-022-03138-x 36056380 PMC9438174

[56] Roberts B, Hendershott MC, Arakaki J, Gerbin KA, Malik H, Nelson A, et al. Fluorescent Gene Tagging of Transcriptionally Silent Genes in hiPSCs. Stem Cell Reports. 2019. doi: 10.1016/j.stemcr.2019.03.001 PMC6522946 30956114

[57] Somers A, Jean J-C, Sommer CA, Omari A, Ford CC, Mills JA, et al. Generation of transgene-free lung disease-specific human induced pluripotent stem cells using a single excisable lentiviral stem cell cassette. Stem Cells. 2010;28(10):1728–40. doi: 10.1002/stem.495 20715179 PMC3422663

[58] Moreno-Mateos MA, Vejnar CE, Beaudoin J-D, Fernandez JP, Mis EK, Khokha MK, et al. CRISPRscan: designing highly efficient sgRNAs for CRISPR-Cas9 targeting in vivo. Nat Methods. 2015;12(10):982–8. doi: 10.1038/nmeth.3543 26322839 PMC4589495

[59] Vejnar CE, Moreno-Mateos MA, Cifuentes D, Bazzini AA, Giraldez AJ. Optimization Strategies for the CRISPR-Cas9 Genome-Editing System. Cold Spring Harb Protoc. 2016;2016(10):10.1101/pdb.top090894. doi: 10.1101/pdb.top090894 27698246

[60] Yu X, Liang X, Xie H, Kumar S, Ravinder N, Potter J, et al. Improved delivery of Cas9 protein/gRNA complexes using lipofectamine CRISPRMAX. Biotechnol Lett. 2016;38(6):919–29. doi: 10.1007/s10529-016-2064-9 26892225 PMC4853464

[61] Takahashi K, Tanabe K, Ohnuki M, Narita M, Ichisaka T, Tomoda K, et al. Induction of pluripotent stem cells from adult human fibroblasts by defined factors. Cell. 2007;131(5):861–72. doi: 10.1016/j.cell.2007.11.019 18035408

[62] Beers J, Gulbranson DR, George N, Siniscalchi LI, Jones J, Thomson JA, et al. Passaging and colony expansion of human pluripotent stem cells by enzyme-free dissociation in chemically defined culture conditions. Nat Protoc. 2012;7(11):2029–40. doi: 10.1038/nprot.2012.130 23099485 PMC3571618

[63] Lian X, Hsiao C, Wilson G, Zhu K, Hazeltine LB, Azarin SM, et al. Robust cardiomyocyte differentiation from human pluripotent stem cells via temporal modulation of canonical Wnt signaling. Proc Natl Acad Sci U S A. 2012;109(27):E1848-57. doi: 10.1073/pnas.1200250109 22645348 PMC3390875

[64] Lin Y, Zou J. Differentiation of Cardiomyocytes from Human Pluripotent Stem Cells in Fully Chemically Defined Conditions. STAR Protoc. 2020;1(1):100015. doi: 10.1016/j.xpro.2020.100015 32734277 PMC7392178

[65] Phipson B, Lee S, Majewski IJ, Alexander WS, Smyth GK. Robust Hyperparameter Estimation Protects Against Hypervariable Genes And Improves Power To Detect Differential Expression. Ann Appl Stat. 2016;10(2):946–63. doi: 10.1214/16-AOAS920 28367255 PMC5373812

[66] Ritchie ME, Phipson B, Wu D, Hu Y, Law CW, Shi W, et al. limma powers differential expression analyses for RNA-sequencing and microarray studies. Nucleic Acids Res. 2015;43(7):e47. doi: 10.1093/nar/gkv007 25605792 PMC4402510

[67] Carpenter AE, Jones TR, Lamprecht MR, Clarke C, Kang IH, Friman O, et al. CellProfiler: image analysis software for identifying and quantifying cell phenotypes. Genome Biol. 2006;7(10):R100. doi: 10.1186/gb-2006-7-10-r100 17076895 PMC1794559

[68] Schindelin J, Arganda-Carreras I, Frise E, Kaynig V, Longair M, Pietzsch T, et al. Fiji: an open-source platform for biological-image analysis. Nat Methods. 2012;9(7):676–82. doi: 10.1038/nmeth.2019 22743772 PMC3855844

[69] Homan T, Delanoë-Ayari H, Meli AC, Cazorla O, Gergely C, Mejat A, et al. MorphoScript: a dedicated analysis to assess the morphology and contractile structures of cardiomyocytes derived from stem cells. Bioinformatics. 2021;37(22):4209–15. doi: 10.1093/bioinformatics/btab400 34048539

[70] Scalzo S, Afonso MQL, da Fonseca NJ Jr, Jesus ICG, Alves AP, Mendonça CATF, et al. Dense optical flow software to quantify cellular contractility. Cell Rep Methods. 2021;1(4):100044. doi: 10.1016/j.crmeth.2021.100044 35475144 PMC9017166

[71] Ameen M, Sundaram L, Shen M, Banerjee A, Kundu S, Nair S, et al. Integrative single-cell analysis of cardiogenesis identifies developmental trajectories and non-coding mutations in congenital heart disease. Cell. 2022;185(26):4937-4953.e23. doi: 10.1016/j.cell.2022.11.028 36563664 PMC10122433

[72] Gonzalez-Teran B, Pittman M, Felix F, Thomas R, Richmond-Buccola D, Hüttenhain R, et al. Transcription factor protein interactomes reveal genetic determinants in heart disease. Cell. 2022;185(5):794-814.e30. doi: 10.1016/j.cell.2022.01.021 35182466 PMC8923057

[73] Wang Q, Li M, Wu T, Zhan L, Li L, Chen M, et al. Exploring Epigenomic Datasets by ChIPseeker. Curr Protoc. 2022;2(10):e585. doi: 10.1002/cpz1.585 36286622

[74] Buenrostro JD, Wu B, Litzenburger UM, Ruff D, Gonzales ML, Snyder MP, et al. Single-cell chromatin accessibility reveals principles of regulatory variation. Nature. 2015;523(7561):486–90. doi: 10.1038/nature14590 26083756 PMC4685948

[75] Schep AN, Wu B, Buenrostro JD, Greenleaf WJ. chromVAR: inferring transcription-factor-associated accessibility from single-cell epigenomic data. Nat Methods. 2017;14(10):975–8. doi: 10.1038/nmeth.4401 28825706 PMC5623146

[76] Berger MF, Badis G, Gehrke AR, Talukder S, Philippakis AA, Peña-Castillo L, et al. Variation in homeodomain DNA binding revealed by high-resolution analysis of sequence preferences. Cell. 2008;133(7):1266–76. doi: 10.1016/j.cell.2008.05.024 18585359 PMC2531161

[77] Jolma A, Yan J, Whitington T, Toivonen J, Nitta KR, Rastas P, et al. DNA-binding specificities of human transcription factors. Cell. 2013;152(1–2):327–39. doi: 10.1016/j.cell.2012.12.009 23332764

[78] Lawrence M, Gentleman R, Carey V. rtracklayer: an R package for interfacing with genome browsers. Bioinformatics. 2009;25(14):1841–2. doi: 10.1093/bioinformatics/btp328 19468054 PMC2705236

[79] Zhu LJ, Gazin C, Lawson ND, Pagès H, Lin SM, Lapointe DS, et al. ChIPpeakAnno: a Bioconductor package to annotate ChIP-seq and ChIP-chip data. BMC Bioinformatics. 2010;11:237. doi: 10.1186/1471-2105-11-237 20459804 PMC3098059

[80] Wu T, Hu E, Xu S, Chen M, Guo P, Dai Z. Clusterprofiler 4.0: A universal enrichment tool for interpreting omics data. Innovation. 2021;2. doi: 10.1016/j.xinn.2021.100141 PMC8454663 34557778

