# Supplementary material for: IRX3 depletion promotes early cardiac commitment of hiPSC-Derived Cardiomyocytes
Source: PLoS One. 2026 Jun 16;21(6):e0351704. doi: 10.1371/journal.pone.0351704 (PMC13271448; doi:10.1371/journal.pone.0351704)
Supplement: S2 Fig — Protein alignment for human IRX3 against predicted encoded peptides of IRX3 mutant alleles. Transheterozygous alleles are indicated by allele number (1 and 2). IRX3cl1.2 allele 2 is a 227 bp deletion encompassing the IRX3 transcription start site (TSS), indicated by the red *. A potential alternative TSS is indicated by the black arrowhead. (PDF) [file pone.0351704.s002.pdf]

```
IRX3cl+/+          MSFPQLGYQYIRPLYPSERPGAAGGSGGSAGARGGLGAGASELNASGSLSNVLSSVYGAPY   61
IRX3cl1.2 | 2      -------------------------------------------------------------   0
IRX3cl1.1 | 1      MSFPQLGYQYIRPLYPSERPGAAGGSGGSAGARGGLGAGASELNASGSLSNVV--------   53
IRX3cl1.1 | 2      MSFPQLGYQYIRPLYPSERPGAAGGSGGSAGARGGLGAGASELNASGSLSNVLSSVYGAPY   61
IRX3cl1.2 | 1      MSFPQLGYQYIRPLYPSERPGAAGGSGGSAGARGGLGAGASELNASGSLSNVLSSVYGAPY   61
IRX3cl2 | 1 and 2  MSFPQLGYQYIRPLYPSERPGAAGGSGGSAGARGGLGAGASELNASGSLSNVLSSV*----   57

IRX3cl+/+          AAAAAAAAAQGYGAFLPYAAELPIFPQLGAQYELKDSPGVQHPAAAAAFPHPHPAFYPYGQ   122
IRX3cl1.2 | 2      -------------------------------------------------------------   0
IRX3cl1.1 | 1      -------------------RRLPALRRGAAHL----------PAAGRAV*-----------   74
IRX3cl1.1 | 2      RGRCGRRRP--------RLRRLPALRRGAAHL----------PAAGRAV*-----------   93
IRX3cl1.2 | 1      RGRCGRRRP--------RLRRLPALRRGAAHL----------PAAGRAV*-----------   93
IRX3cl2 | 1 and 2  -------------------------------------------------------------   57

IRX3cl+/+          YQFGDPSRPKNATRESTSTLKAWLNEHRKNPYPTKGEKIMLAIITKMTLTQVSTWFANARR   183
IRX3cl1.2 | 2      ---------------------------------------MLAIITKMTLTQVSTWFANARR   22
IRX3cl1.1 | 1      -------------------------------------------------------------   74
IRX3cl1.1 | 2      -------------------------------------------------------------   93
IRX3cl1.2 | 1      -------------------------------------------------------------   93
IRX3cl2 | 1 and 2  -------------------------------------------------------------   57

IRX3cl+/+          RLKKENKMTWAPRSRTDEEGNAYGSEREEEDEEEDEEDGKRELELEEEELGGEEEDTGGEG   244
IRX3cl1.2 | 2      RLKKENKMTWAPRSRTDEEGNAYGSEREEEDEEEDEEDGKRELELEEEELGGEEEDTGGEG   83
IRX3cl1.1 | 1      -------------------------------------------------------------   74
IRX3cl1.1 | 2      -------------------------------------------------------------   93
IRX3cl1.2 | 1      -------------------------------------------------------------   93
IRX3cl2 | 1 and 2  -------------------------------------------------------------   57

IRX3cl+/+          LADDDEDEEIDLENLDGAATEPELSLAGAARRDGDLGLGPISDSKNSDSEDSSEGLEDRPL   305
IRX3cl1.2 | 2      LADDDEDEEIDLENLDGAATEPELSLAGAARRDGDLGLGPISDSKNSDSEDSSEGLEDRPL   144
IRX3cl1.1 | 1      -------------------------------------------------------------   74
IRX3cl1.1 | 2      -------------------------------------------------------------   93
IRX3cl1.2 | 1      -------------------------------------------------------------   93
IRX3cl2 | 1 and 2  -------------------------------------------------------------   57

IRX3cl+/+          PVLSLAPAPPPVAVASPSLPSPPVSLDPCAPAPAPASALQKPKIWSLAETATSPDNPRRSP   366
IRX3cl1.2 | 2      PVLSLAPAPPPVAVASPSLPSPPVSLDPCAPAPAPASALQKPKIWSLAETATSPDNPRRSP   205
IRX3cl1.1 | 1      -------------------------------------------------------------   74
IRX3cl1.1 | 2      -------------------------------------------------------------   93
IRX3cl1.2 | 1      -------------------------------------------------------------   93
IRX3cl2 | 1 and 2  -------------------------------------------------------------   57

IRX3cl+/+          PGAGGSPPGAAVAPSALQLSPAAAAAAAHRLVSAPLGKFPAWTNRPFPGPPPGPRLHPLSL   427
IRX3cl1.2 | 2      PGAGGSPPGAAVAPSALQLSPAAAAAAAHRLVSAPLGKFPAWTNRPFPGPPPGPRLHPLSL   266
IRX3cl1.1 | 1      -------------------------------------------------------------   74
IRX3cl1.1 | 2      -------------------------------------------------------------   93
IRX3cl1.2 | 1      -------------------------------------------------------------   93
IRX3cl2 | 1 and 2  -------------------------------------------------------------   57

IRX3cl+/+          LGSAPPHLLGLPGAAGHPAAAAAFARPAEPEGGTDRCSALEVEKKLLKTAFQPVPRRPQNH   488
IRX3cl1.2 | 2      LGSAPPHLLGLPGAAGHPAAAAAFARPAEPEGGTDRCSALEVEKKLLKTAFQPVPRRPQNH   327
IRX3cl1.1 | 1      -------------------------------------------------------------   74
IRX3cl1.1 | 2      -------------------------------------------------------------   93
IRX3cl1.2 | 1      -------------------------------------------------------------   93
IRX3cl2 | 1 and 2  -------------------------------------------------------------   57

IRX3cl+/+          LDAALVLSALSSS*                                                  502
IRX3cl1.2 | 2      LDAALVLSALSSS*                                                  341
IRX3cl1.1 | 1      --------------                                                  74
IRX3cl1.1 | 2      --------------                                                  93
IRX3cl1.2 | 1      --------------                                                  93
IRX3cl2 | 1 and 2  --------------                                                  57
```
