# Supplementary figures and images for: IRX3 depletion promotes early cardiac commitment of hiPSC-Derived Cardiomyocytes
Source: PLoS One. 2026 Jun 16;21(6):e0351704. doi: 10.1371/journal.pone.0351704 (PMC13271448; doi:10.1371/journal.pone.0351704)

**A**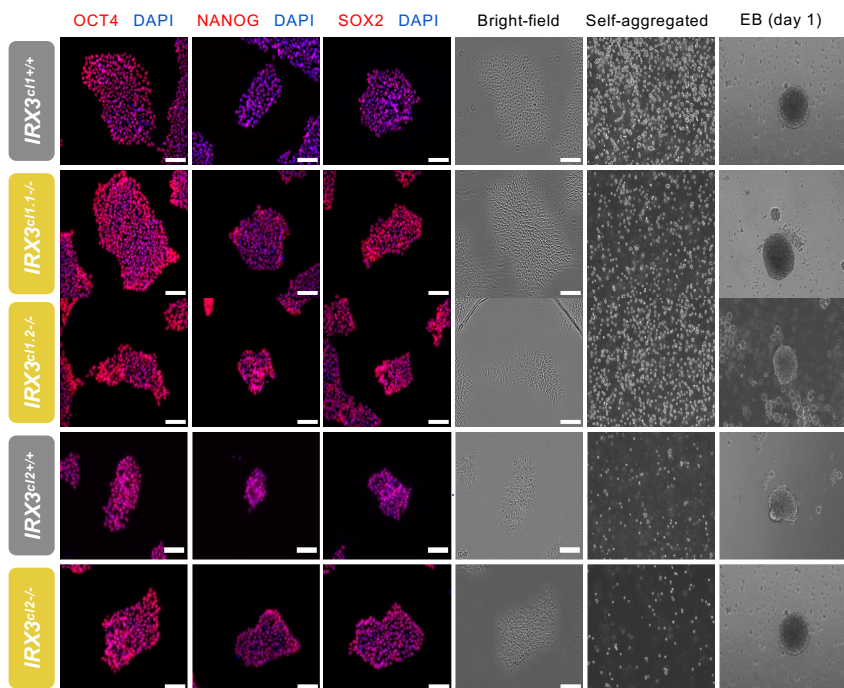**B**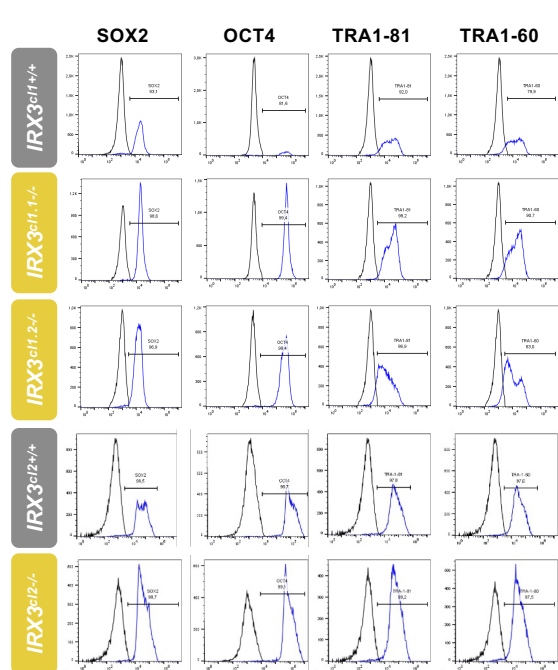**C**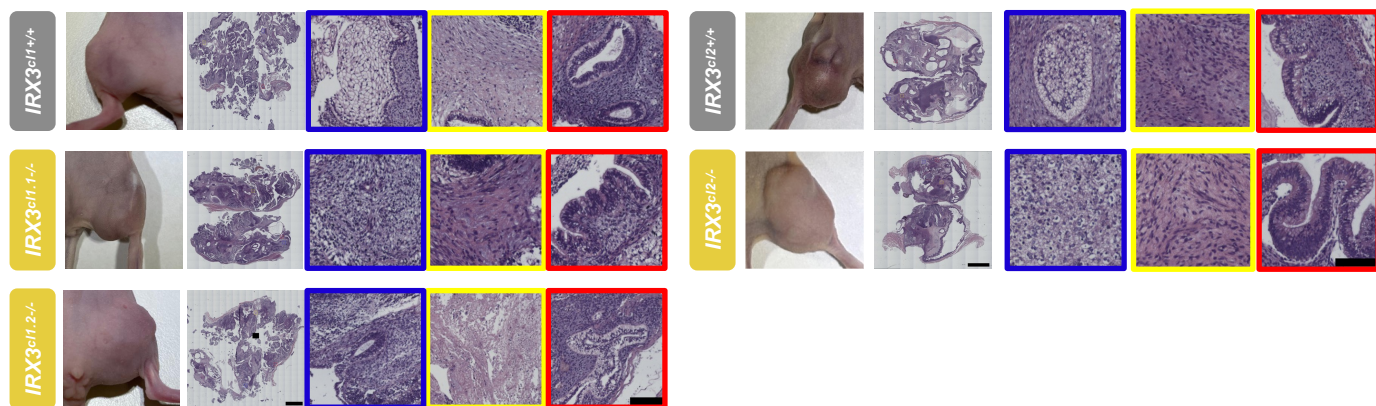

Supplement: S3 Fig — (A) Representative immunofluorescence of the pluripotency markers OCT4, NANOG and SOX2 in IRX3-KO hiPSC clones. Images showing morphology, self-aggregation and embryoid body (EB) formation on phase-contrast microscopy. (B) Representative flow cytometry of pluripotency markers SOX2, OCT4, TRA1–81 and TRA1–60 in IRX3-KO hiPSC clones. (C) Teratoma formation assay. Representative images show gross morphology (left column) and low-magnification H&E staining of whole tumor sections (second column; scale bar: 2 mm). Colored boxes (scale bar: 100 µm) highlight organized tissues confirming differentiation into all three germ layers: Ectoderm (blue; neuroepithelium and epithelial cysts), Mesoderm (yellow; skeletal muscle and connective tissue), and Endoderm (red; gut-like epithelium and glandular epithelium). (PDF) [file pone.0351704.s003.pdf]

**A**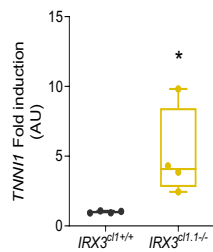**B**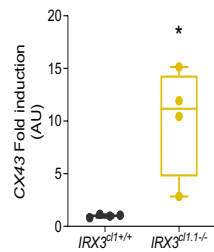**C**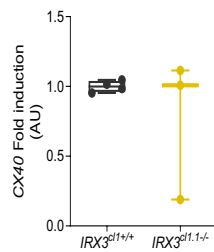**D**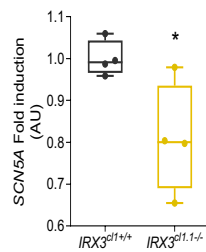

Supplement: S4 Fig — (A-D) Relative mRNA levels of (A) TNNI1, (B) GJA1, (C) GJA5, and (D) SCN5A genes (n = 3–4 independent replicates) from IRX3cl1+/+ and IRX3cl1.1-/- hiPSC-CMs at day 15 of differentiation. Data are presented as box plots with individual replicates. Student’s t-test. *P < 0.05 vs IRX3cl1+/+. (PDF) [file pone.0351704.s004.pdf]

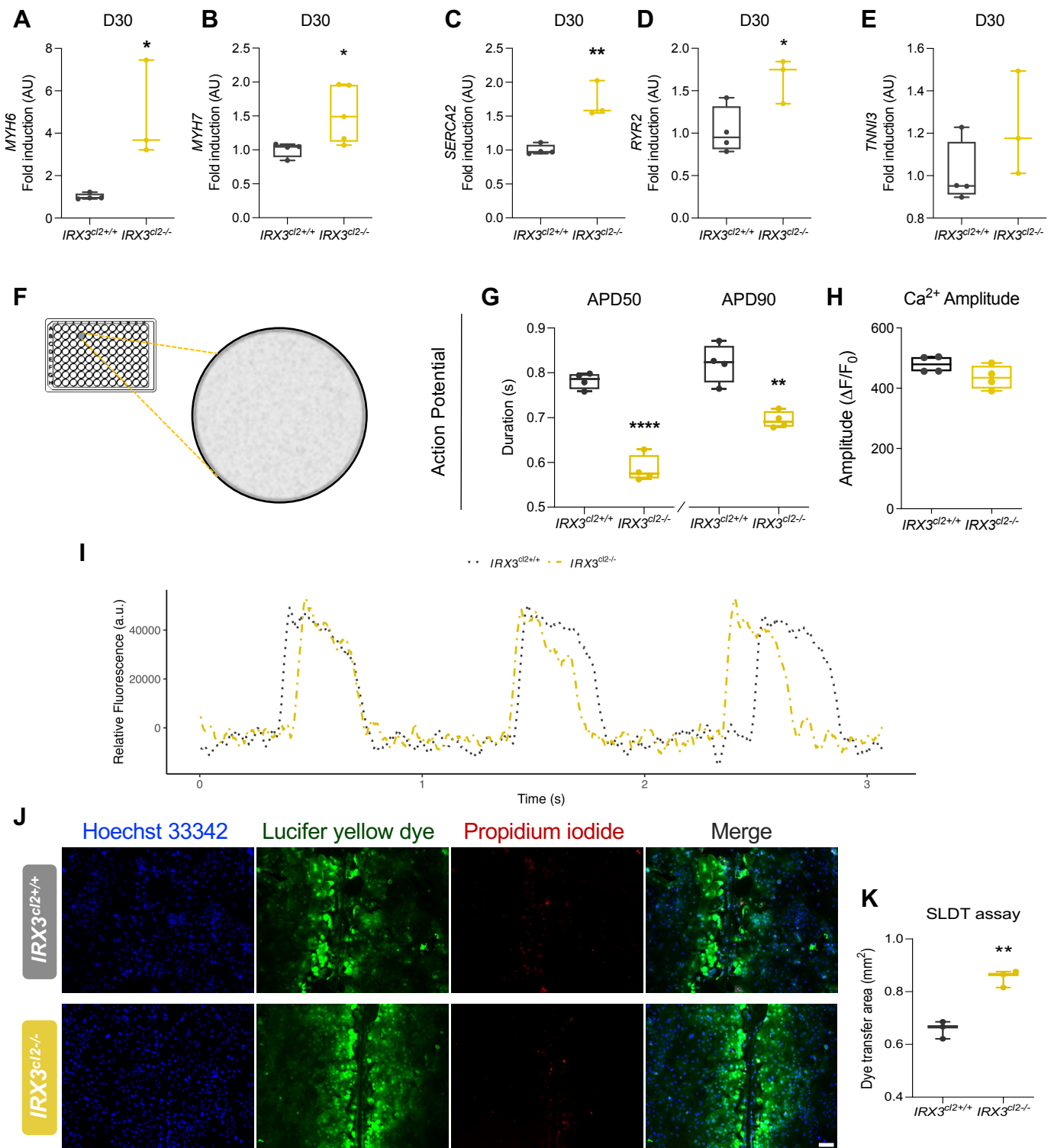

Supplement: S5 Fig — (A-E) Relative mRNA levels of (A) MYH6, (B) MYH7, (C) SERCA2, (D) RYR2, and (E) TNNI3 in IRX3cl2-/- hiPSC-CMs at day 30 of differentiation (n = 3–4 independent replicates). (F) Schematic figure of analyzed field of view of hiPSC-CMs self-arranged in a monolayer syncytium-like form. (G) Action potential duration at 50% (APD50) and 90% (APD90) of repolarization and (H) Action potential amplitude (n = 4 independent replicates). (I) Representative curves of action potentials. (J) Representative immunofluorescence of scrape loading dye transfer assay (SLDT) – living cells were stained with Lucifer yellow dye (green), dead cells were stained with Propidium iodide (red), and cell nuclei were counterstained with Hoechst 33342 (blue). (K) Quantification of the dye transfer area (n = 3 independent replicates). Scale bars = 20 µm. Student’s t-test. Data are presented as box plots with individual replicates. *P < 0.05; **P < 0.01; ****P < 0.0001 vs IRX3cl2+/+. (PDF) [file pone.0351704.s005.pdf]

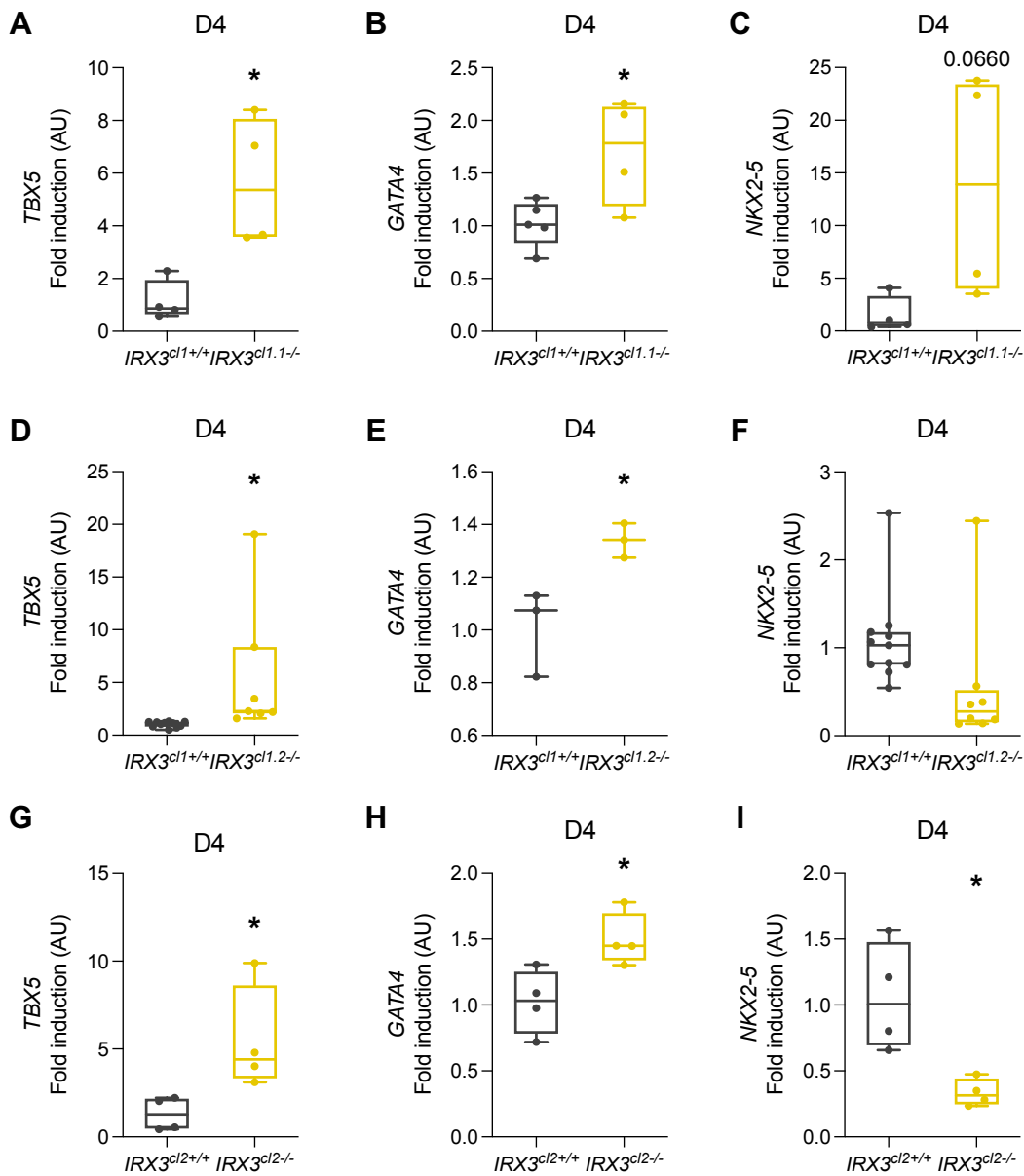

Supplement: S6 Fig — (A-I) Relative mRNA levels of TBX5, GATA4, and NKX2–5 in (A-C) IRX3cl1.1-/-, (D-F) IRX3cl1.2-/-, and (G-I) IRX3cl2-/- cells, respectively (n = 4–10 independent replicates). Data are presented as box plots with individual replicates. Student’s t-test. *P < 0.05 vs IRX3cl1+/+ or IRX3cl2+/+. (PDF) [file pone.0351704.s006.pdf]

Irx3\_bulyk\_cell08-0920.1

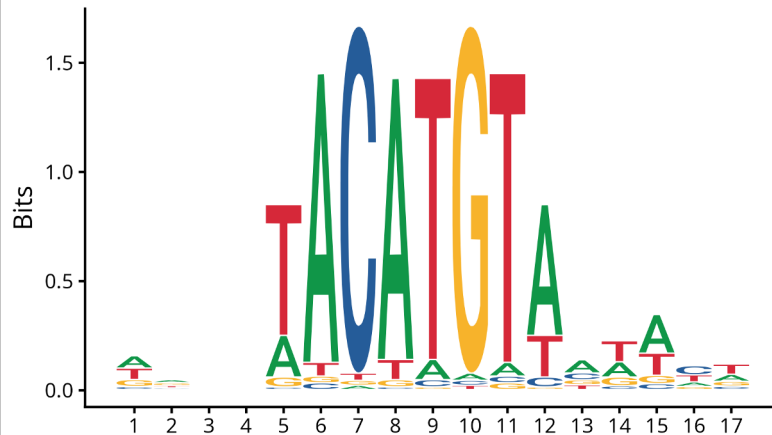

Irx3\_bulyk\_cell08-2226.1

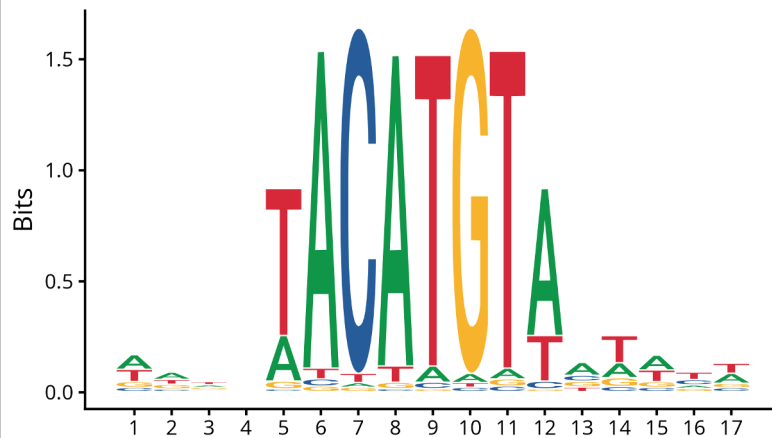

Irx3\_jolma\_DBD\_M568

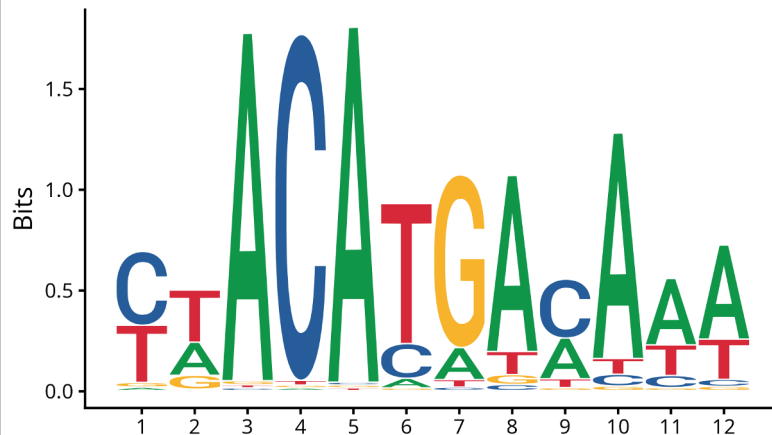

Supplement: S7 Fig — (A) Sequence logos illustrating the binding specificity of IRX3 across independent datasets. (Top/Middle) Motifs identified by Berger et al. [76] characterized by a conserved ACATGT core sequence. (Bottom) The IRX3 motif from the Jolma et al. dataset [77], exhibiting a variant ACATGA core. The height of each letter (y-axis) represents the information content (bits) and the relative frequency of the nucleotide at that specific position (x-axis), indicating the strictness of the binding preference. (PDF) [file pone.0351704.s007.pdf]

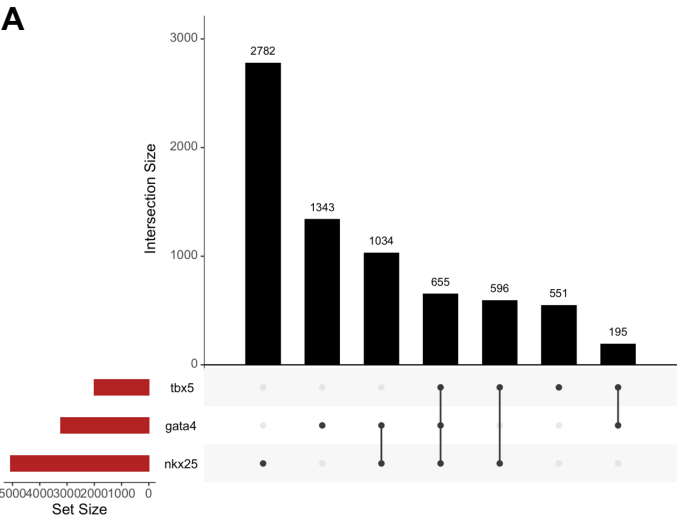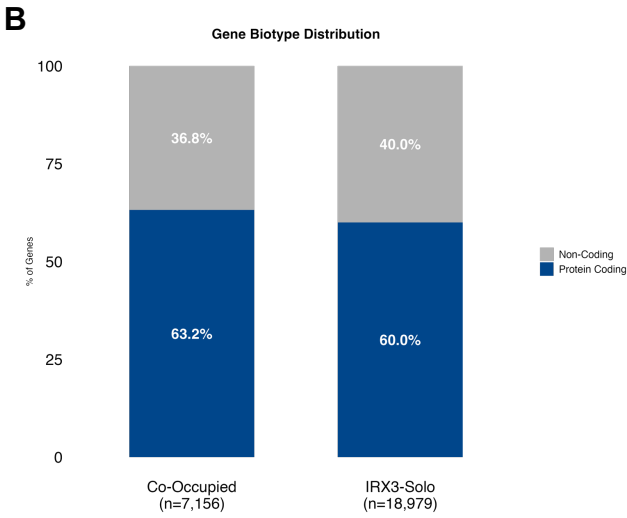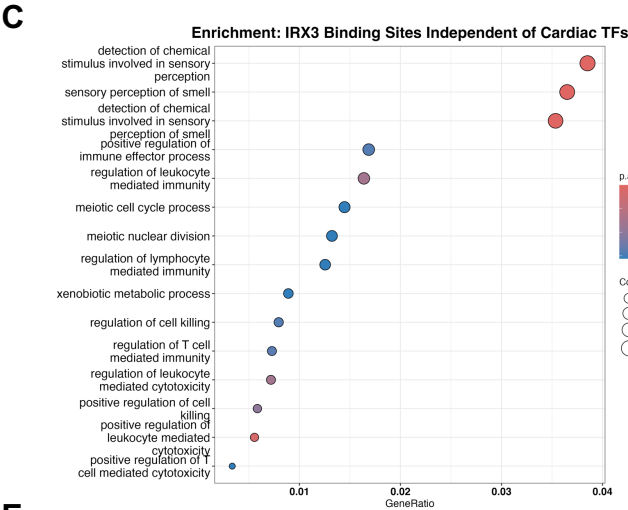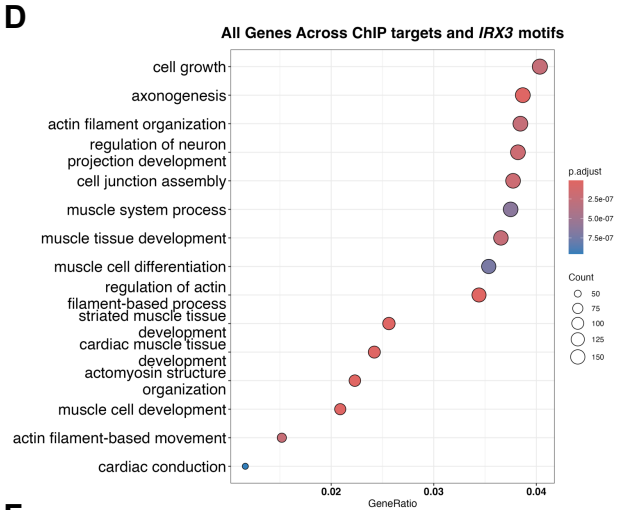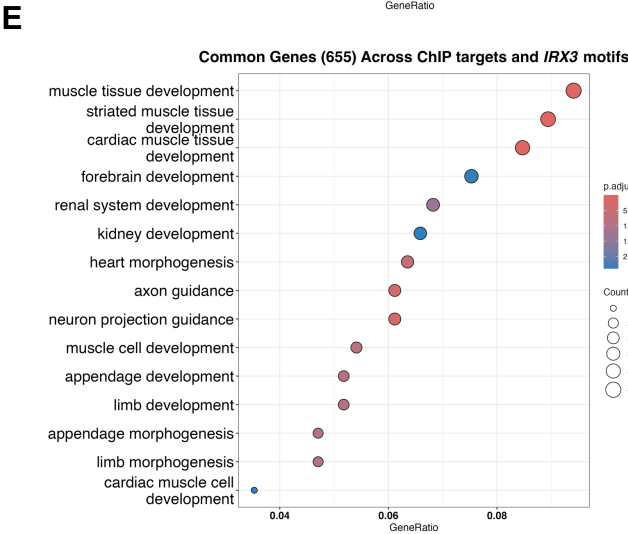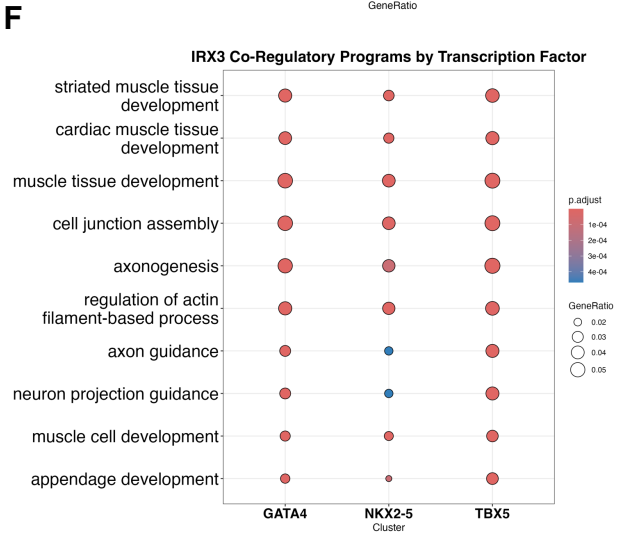

Supplement: S8 Fig — (A) UpSet plot visualizing the intersection of IRX3 target genes with NKX2–5, GATA4, and TBX5 binding profiles. Vertical bars represent the size of each unique intersection (co-occupied gene sets), while horizontal bars (left) show the total number of IRX3 targets overlapping with each individual transcription factor. The matrix diagram indicates the specific combination of factors present at these loci. (B) Distribution of gene biotypes (Protein-Coding vs. Non-Coding) across the ‘Co-Occupied’ and ‘IRX3-Solo’ target gene populations. (C-F) GO enrichment analysis of biological processes. Analysis was performed on: (C) the ‘IRX3-solo’ gene population; (D) the aggregate union of genes co-occupied by IRX3 and at least one cardiac TF; (E) the core intersection of genes co-occupied by IRX3 and all three cardiac TFs simultaneously; and (F) specific target subsets defined by IRX3 co-binding with GATA4, NKX2–5, or TBX5 individually (see S2 Table for the complete data set). (PDF) [file pone.0351704.s008.pdf]

A

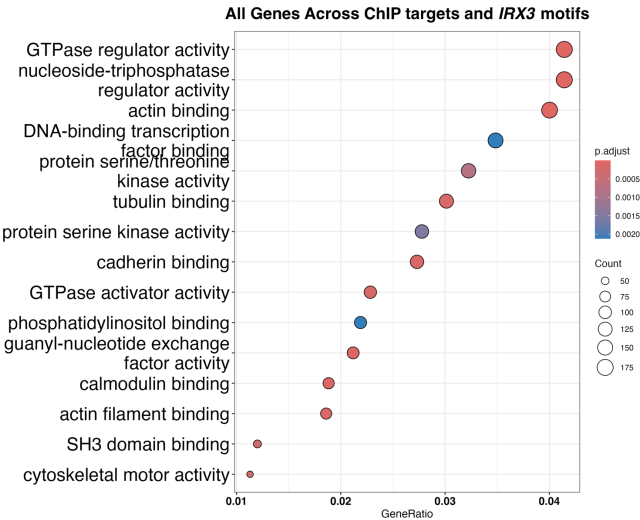

B

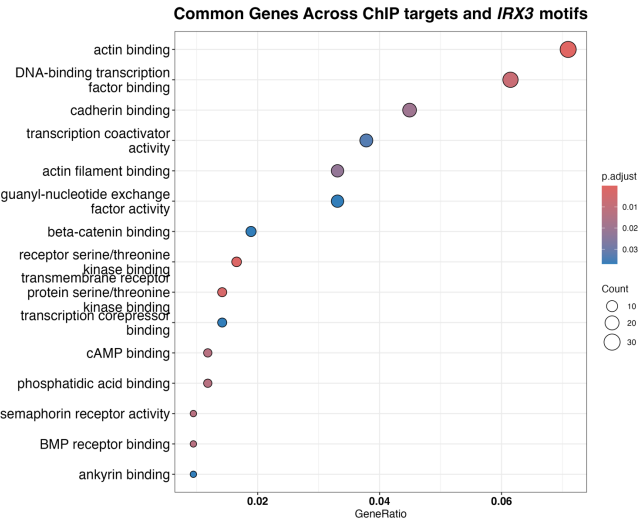

C

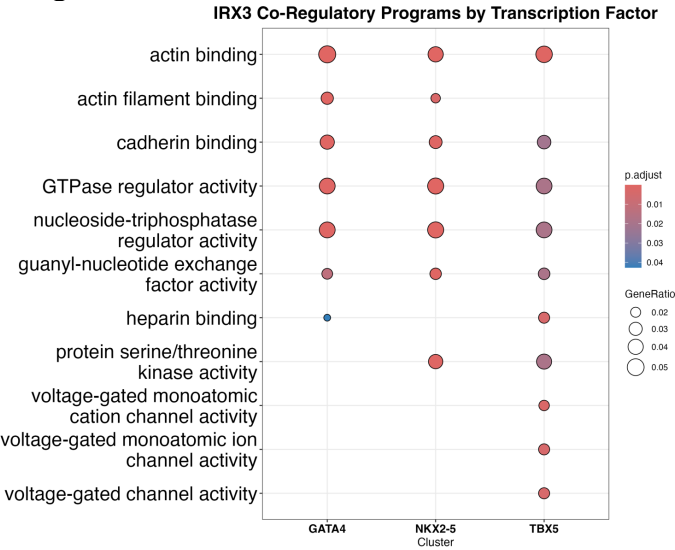

Supplement: S9 Fig — The percentage of shared peaks is shown on the x-axis, representing the proportion of scATAC-seq peaks that contain an IRX3 motif and overlap with binding sites for the indicated TFs (y-axis). Colors indicate the specific genomic region where the overlap occurs. The data highlight that the majority of significant overlaps fall within promoter regions, suggesting a critical role for IRX3 and these TFs in gene regulation. (PDF) [file pone.0351704.s009.pdf]

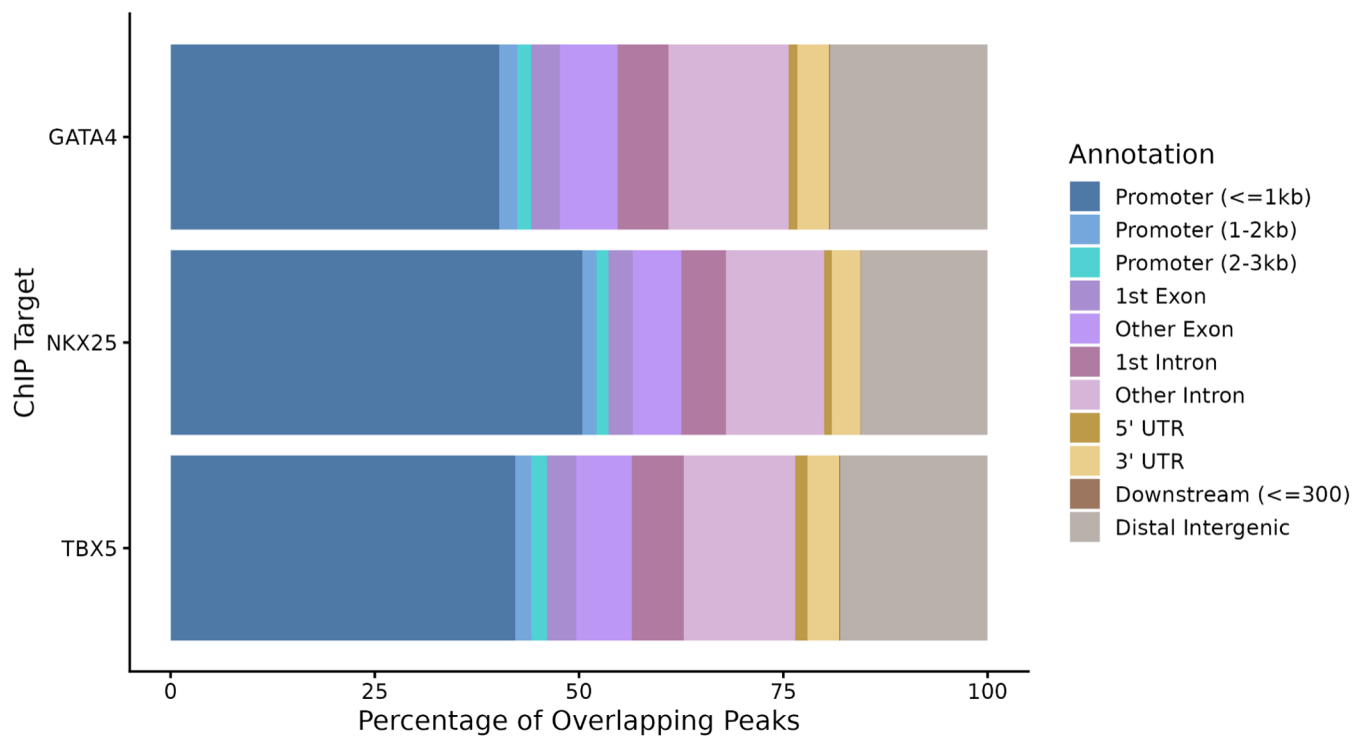

Supplement: S10 Fig — (A-C) GO terms associated with molecular functions for (A) all genes encountered, (B) common genes across ChIP-seq targets and IRX3 motifs, and (C) genes with IRX3 motifs and ChIP-seq peaks for either GATA4, NKX2–5, or TBX5. (PDF) [file pone.0351704.s010.pdf]
