# Supplementary material for: IRX3 depletion promotes early cardiac commitment of hiPSC-Derived Cardiomyocytes
Source: PLoS One. 2026 Jun 16;21(6):e0351704. doi: 10.1371/journal.pone.0351704 (PMC13271448; doi:10.1371/journal.pone.0351704)
Supplement: S1 Fig — (A-C) Allele description of (A) IRX3cl1.1-/-, (B) IRX3cl1.2-/-, and (C) IRX3cl2-/- mutant hiPSC clones. (D) Sequencing alignment of IRX3-KO hiPSC subclones. gRNA + PAM sequences are shown in green. (E) Relative mRNA levels of IRX3 in IRX3-KO hiPSC clones at day 4 of differentiation (n = 3 independent replicates). (F-G) Representative images of karyotype analysis of (F) IRX3cl1.1-/-, IRX3cl1.2-/-, and IRX3cl2-/- hiPSCs and (G) of IRX3cl2+/+ and IRX3cl2-/-. Data are presented as box plots with individual replicates. Student’s t-test. *P < 0.05; ***P < 0.001 vs IRX3cl1+/+. (PDF) [file pone.0351704.s001.pdf]

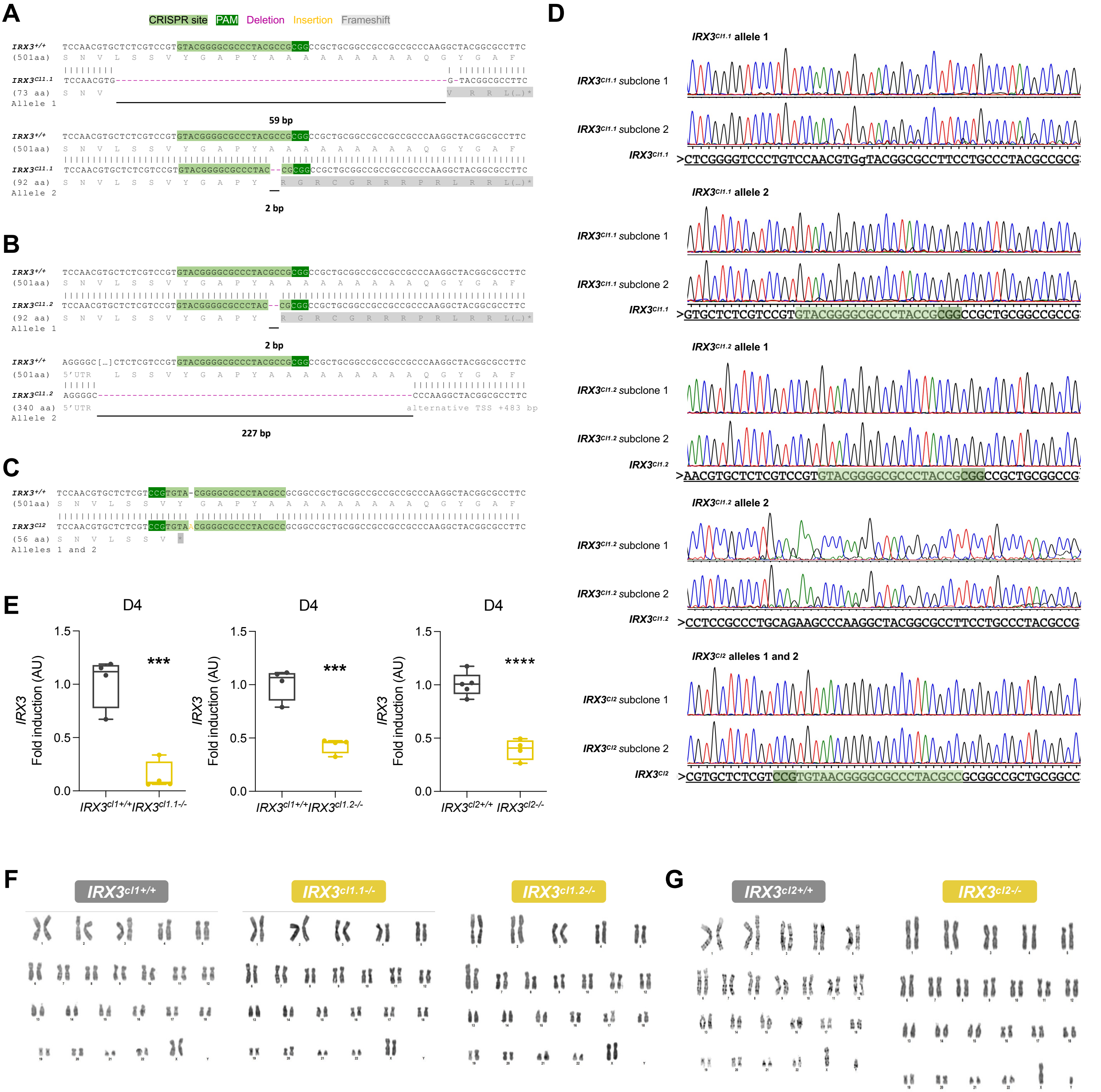

A
CRISPR site
PAM
Deletion
Insertion
Frameshift
IRX3+/+
(501aa)
IRX3cl1.1
(73 aa)
Allele 1
59 bp
(92 aa)
Allele 2
2 bp
B
IRX3cl1.2
(340 aa)
5'UTR
alternative TSS +483 bp
227 bp
C
IRX3cl2
(56 aa)
Alleles 1 and 2
D
IRX3cl1.1 allele 1
IRX3cl1.1 subclone 1
IRX3cl1.1 subclone 2
IRX3cl1.1 allele 2
IRX3cl1.2 allele 1
IRX3cl1.2 subclone 1
IRX3cl1.2 subclone 2
IRX3cl1.2 allele 2
IRX3cl2 alleles 1 and 2
IRX3cl2 subclone 1
IRX3cl2 subclone 2
E
D4
IRX3
Fold induction (AU)
***
****
IRX3cl1+/+
IRX3cl1.1-/-
IRX3cl1.2-/-
IRX3cl2+/+
IRX3cl2-/-
F
IRX3cl1+/+
IRX3cl1.1-/-
IRX3cl1.2-/-
G
IRX3cl2+/+
IRX3cl2-/-
