# Supplementary material for: IRX3 depletion promotes early cardiac commitment of hiPSC-Derived Cardiomyocytes
Source: PLoS One. 2026 Jun 16;21(6):e0351704. doi: 10.1371/journal.pone.0351704 (PMC13271448; doi:10.1371/journal.pone.0351704)
Supplement: Attachment — Submitted filename: Response to Reviewers_editor.pdf [file pone.0351704.s019.pdf]

Dear Editor and Reviewers,

We thank the reviewer for confirming our previous revisions and for identifying these typographical errors. We have made all the requested corrections and performed a final comprehensive proofreading of the manuscript.

In addition, we uploaded updated versions of Figures 1 and 2 to correct a minor visual cropping issue present in the previous rendering. These changes are strictly cosmetic; no underlying data, analyses, results, or conclusions were altered.

Thank you again for your careful review and valuable contributions to improving our manuscript.

Sincerely,
