# Supplementary material for: IRX3 depletion promotes early cardiac commitment of hiPSC-Derived Cardiomyocytes
Source: PLoS One. 2026 Jun 16;21(6):e0351704. doi: 10.1371/journal.pone.0351704 (PMC13271448; doi:10.1371/journal.pone.0351704)
Supplement: Attachment — Submitted filename: Response to reviewers.docx [file pone.0351704.s017.docx]

**Review Comments to the Author**

Dear Dr. Schubert and Members of the Review Panel,

Please find attached our revised manuscript for consideration as an Article in *PLOS ONE*. We thank you for your initial positive response and for giving us the opportunity to improve and resubmit our work.

We are also grateful to the reviewers for their constructive remarks and insightful suggestions. We have carefully addressed all major and minor comments in the point-by-point response below. To significantly strengthen the manuscript and address the specific concerns raised, we have incorporated the following key revisions:

1. **Genetic Validation:** We have provided a comprehensive genetic characterization of all our hiPSC clones to ensure rigor and reproducibility.
2. **Functional Consistency:** We have included calcium transient data for both independent clones (*IRX3^Cl1.1-/-^ and IRX3^Cl1.2-/-^*), demonstrating that the increased percentage of TnI+ cardiomyocytes is accompanied by consistent functional changes across mutagenesis experiments.
3. **Transcriptional Profiling:** We have added gene expression data describing the early upregulation of cardiac transcription factors (*TBX5*, *GATA4*, *NKX2-5*) at Day 4, as well as the expression of distinct maturation markers at Day 30.
4. **Refined *In Silico* Analysis:** We have performed a new co-occupancy analysis (utilizing UpSet plots and Gene Ontology enrichment) to clarify the functional distinction between "IRX3-Solo" binding sites and those co-occupied by core cardiac factors.

Thank you for considering our revised manuscript.

Sincerely,

The Authors

**Journal Requirements:**

When submitting your revision, we need you to address these additional requirements.

1.Please ensure that your manuscript meets PLOS ONE's style requirements, including those for file naming. The PLOS ONE style templates can be found at

<https://journals.plos.org/plosone/s/file?id=wjVg/PLOSOne_formatting_sample_main_body.pdf> and [https://journals.plos.org/plosone/s/file?id=ba62/PLOSOne_formatting_sample_title_authors_affiliations.pdf](https://track.editorialmanager.com/CL0/https:%2F%2Fjournals.plos.org%2Fplosone%2Fs%2Ffile%3Fid=ba62%2FPLOSOne_formatting_sample_title_authors_affiliations.pdf/1/010f01997a89ceda-ab3c984e-4df9-45f3-8ff1-6736526643c9-000000/FljgqRbAQHSoWx3UIz9Xp70TqRdap_bA3WEfBeN1i4k=230)

Response: We have reviewed the manuscript and files to ensure compliance with PLOS ONE style and file naming requirements.

2. When you submit your revised manuscript, please correct the dates of the cell line ethical approval in our online submission portal. You state that ethics approval 0055/11 was granted on 20 September 2019, while in the manuscript and the ethics document itself, the date is stated to be 21 March 2012. Thank you.

Response: We have corrected the ethical approval date in the online submission portal, ensuring consistency with the manuscript and the original ethics document.

3. Please note that funding information should not appear in any section or other areas of your manuscript. We will only publish funding information present in the Funding Statement section of the online submission form. Please remove any funding-related text from the manuscript.

Response: We have removed all funding-related text from the manuscript file. Funding information is now provided exclusively in the Funding Statement section of the online submission form.

4. We note that the grant information you provided in the ‘Funding Information’ and ‘Financial Disclosure’ sections do not match.

When you resubmit, please ensure that you provide the correct grant numbers for the awards you received for your study in the ‘Funding Information’ section.

Response: We have verified the grant numbers and ensured that the "Funding Information" is now accurate.

5. Thank you for stating the following financial disclosure:

“This work was supported by grants from the Fundação de Amparo à Pesquisa do Estado de São Paulo (FAPESP) [INCT - 20214/50889-7 to JEK, 2013/17368-0 to JEK, 2015/50216-5 to JEK, 2017/05829-4 to ARS, 2021/14876-1 to NDF, 2023/05709-0 to NDF] and from the Conselho Nacional de Pesquisa (CNPq) [INCT - 465586/2014-7 to JEK, 309179/2013-0 to JEK, 442643/2020-9 to JEK].”

Please state what role the funders took in the study. If the funders had no role, please state: "The funders had no role in study design, data collection and analysis, decision to publish, or preparation of the manuscript."

If this statement is not correct you must amend it as needed.

Please include this amended Role of Funder statement in your cover letter; we will change the online submission form on your behalf.

Response: As requested, we provide the following statement regarding the role of the funders: *"The funders had no role in study design, data collection and analysis, decision to publish, or preparation of the manuscript."*

6. Your ethics statement should only appear in the Methods section of your manuscript. If your ethics statement is written in any section besides the Methods, please move it to the Methods section and delete it from any other section. Please ensure that your ethics statement is included in your manuscript, as the ethics statement entered into the online submission form will not be published alongside your manuscript.

Response: We have revised the manuscript to ensure the Ethics Statement appears exclusively within the Methods section (Lines 414-443), in accordance with the journal's guidelines. Any instances of this statement in other sections have been removed.

7. Please ensure that you refer to Figure 6 in your text as, if accepted, production will need this reference to link the reader to the figure.

Response: We appreciate the Editor noting the missing citation for Figure 6. This image was originally intended as a Graphical Abstract. We have decided to remove Figure 6. Upon review, we felt the schematic implied a deterministic mechanism that extends beyond the immediate scope of the data presented in this manuscript. We prefer to keep the conclusions strictly limited to what we demonstrate in the results.

8. Please upload a copy of Figure 7, to which you refer in your text on page 32. If the figure is no longer to be included as part of the submission please remove all reference to it within the text.

Response: The reference to 'Figure 7' was a typographical error. We have corrected the text on page 32 to remove it.

9. PLOS ONE now requires that authors provide the original uncropped and unadjusted images underlying all blot or gel results reported in a submission’s figures or Supporting Information files. This policy and the journal’s other requirements for blot/gel reporting and figure preparation are described in detail at <https://journals.plos.org/plosone/s/figures#loc-blot-and-gel-reporting-requirements> and <https://journals.plos.org/plosone/s/figures#loc-preparing-figures-from-image-files>. When you submit your revised manuscript, please ensure that your figures adhere fully to these guidelines and provide the original underlying images for all blot or gel data reported in your submission. See the following link for instructions on providing the original image data: <https://journals.plos.org/plosone/s/figures#loc-original-images-for-blots-and-gels>.

Response: We acknowledge this requirement. However, as detailed in our response to the reviewers, we have removed all Western blot data from the revised manuscript due to antibody specificity issues. Consequently, the current submission does not contain any blot or gel figures, and thus no raw images are applicable.

In your cover letter, please note whether your blot/gel image data are in Supporting Information or posted at a public data repository, provide the repository URL if relevant, and provide specific details as to which raw blot/gel images, if any, are not available. Email us at plosone@plos.org if you have any questions.

10. If the reviewer comments include a recommendation to cite specific previously published works, please review and evaluate these publications to determine whether they are relevant and should be cited. There is no requirement to cite these works unless the editor has indicated otherwise.

Response: We have carefully reviewed our references as suggested. In particular, we addressed the observation by Reviewer 1 regarding the distinction between *IRX3* function in the ventricular conduction system versus the working myocardium. We have revised the text and rationale to ensure these previous works are cited accurately and in the correct context.

**Reviewer #1:**

This study by da Silva et al. tests whether suppressing IRX3 enhances human iPSC-cardiomyocyte differentiation. Using CRISPR/Cas9 IRX3-null hiPSC lines, the authors show that IRX3 depletion (knockdown and knockout) increased cardiomyocyte yield, higher expression of ventricular markers (TNNI1, MLC2V), elevated CX43, and reduced SCN5A. Functional assessments indicate that IRX3-deficient cells exhibit improved calcium handling, shorter APDs, greater contraction/relaxation velocities, broader mitochondrial networks, and more ordered sarcomeres. Temporal analysis shows IRX3 expression peaks at day 4 of iPSC-CM differentiation. Its loss upregulates early cardiogenic TFs (GATA4, NKX2-5, TBX5, MEF2, HAND), expands progenitors, and activates Wnt-related programs. The authors utilized publicly available ATAC and ChIP data for integrative analysis and suggested IRX3 co-regulates target genes with GATA4/NKX2-5/TBX5. Together, the authors propose that IRX3 acts as a brake on early cardiogenesis.

Overall, this manuscript presented potentially impactful findings. However, there are several concerns regarding citation accuracy, strength of genetic perturbation, and interpretation of integrative in silico analyses. Clarification and further validation are needed to strengthen the conclusions.

Major comments:

1. While the authors targeted IRX3 for its depletion in hiPSC cells, mRNA and protein expression data in all three different clones showed moderately decreased IRX3 protein level (~40%). Given that IRX3 is not known to be haploinsufficient, it remains unclear whether the observed phenotypes are directly attributable to functional IRX3 loss. The authors should clarify and discuss this limitation.

Response:

We appreciate the reviewer’s inquiry regarding the IRX3 protein levels. We understand the concern that residual protein implies potential haploinsufficiency rather than a complete functional loss. However, upon re-evaluating the molecular consequences of our specific gene edits, we have concluded that the signal detected in the previous Western blot represented non-specific antibody binding (likely to other IRX family members) rather than functional IRX3.

We have now provided a comprehensive genetic characterization of the clones (S1 Fig) to support this conclusion:

1. **Genotype confirms Null Alleles:** To rigorously validate our lines, we generated two subclones from each mutated line (*IRX3^Cl1.1^*, *IRX3^Cl1.2^*, and *IRX3^Cl2^*) and sequenced all six independent subclones. Sequencing confirms that all subclones carry either homozygous (*IRX3^Cl2^*)/transheterozygous (*IRX3^Cl1.1^*and *IRX3^Cl1.2^*) frameshift mutations resulting in early Premature Termination Codons (PTC), or loss of the transcription start site (*IRX3^Cl1.2^* allele 2)(S1D Fig). The sequencing of the subclones confirms the mutations observed in the original clones, as detailed in S1A-C Fig.
2. **Protein Size Discrepancy:** These PTCs are predicted to generate severely truncated peptides lacking the DNA binding domain (stopping at approx. 57-93aa instead of 501aa in the wild type). These truncated peptides would be significantly smaller (<15 kDa) than the wild-type protein (~52 kDa). In our previous Western blots, the residual signal appeared as one band at the wild-type molecular weight. Since our genetic data confirms the absence of a full-length open reading frame, this signal cannot represent the mutant IRX3 protein generated by our lines.
3. **Antibody Cross-Reactivity:** As shown in the Figure R1 below, human IRX transcription factors (IRX1, IRX2, IRX3, IRX4, IRX5, and IRX6) share substantial sequence homology and very similar molecular weights (47–54 kDa). It is likely that the antibody cross-reacts with other IRX family members expressed in these cells, creating a false positive signal at the wild-type size.
4. **mRNA and NMD Context:** Regarding the mRNA levels, we acknowledge that transcripts bearing PTCs are often targeted by Nonsense-Mediated Decay (NMD). However, residual mRNA levels are commonly detected in cells bearing PTCs, as established in previous studies (Extended Data Figure 4 - El-Brolosy et al., *Nature*, 2019; PMID: [30944477](https://pubmed.ncbi.nlm.nih.gov/30944477/), and Figure 1d - Falcucci et al., *Nature*, 2025; PMID: [39939773](https://pubmed.ncbi.nlm.nih.gov/39939773/)). For consistency and transparency, we have added *IRX3* RT-qPCR analyses for all clones at day 4 of differentiation (peak *IRX3* expression) in S1E Fig. While mRNA is detectable, the genetic sequencing confirms these transcripts cannot translate into functional, full-length protein.

Given that the residual Western blot signal contradicts the definitive genetic evidence (which demonstrates the impossibility of full-length protein production), we have removed the misleading Western blot data to prevent confusion. We rely instead on the rigorous genetic validation (sequencing of subclones) and SNP array analysis (to rule out off-targets), confirming that these lines are functional nulls for *IRX3*. Thus, we conclude that the observed phenotypes are attributable to the complete loss of functional IRX3.

**Figure R1. Protein alignment of IRX transcription factors**. (A) Protein alignment for human IRX transcription factors, colored by similarity. IRX 3 DNA binding domain is outlined in black. (B) Approximate molecular weight of IRX transcription factors in kDa.

*(For peer review purposes only).*

2. The authors cited previous papers investigating Irx3 and Irx5, such as Development (2012) and Scientific Reports (2016) and mentioned as follows:

Line 74: Given the repressive role of Irx3 in the differentiation of working CMs in mouse..
Line 91: Given the established role of Irx3 in mouse CM differentiation and maturation in vivo..
Line 125: IRX3 depletion promotes CM differentiation and may bias subtype specification toward ventricular working CMs, consistent with in vivo mouse data

However, previous studies have primarily focused on the role of this factor in the development and maturation of the ventricular conduction system, rather than its repressive effect on working CM differentiation. Therefore, these citations seem not to be correctly used to build rationale and justify their experiments and findings on Irx3, respectively. It should be revised accordingly.

Response:

We thank the reviewer for this clarification. We agree that the cited studies (Gaborit et al., *Development*, 2012; PMID: 22992950; Kim et al., *Sci Rep*, 2016; PMID: 26786475) primarily focus on the role of Irx3/Irx5 in the development and maturation of the ventricular conduction system, rather than directly demonstrating repression of working cardiomyocyte differentiation. We have revised the text to more accurately reflect these findings. Specifically, we now state that IRX3 promotes conduction system fate, which indirectly limits working cardiomyocyte identity, rather than attributing a direct repressive role. We also emphasize that our data provide novel evidence for a direct link between *IRX3* depletion and promotion of working CM specification in human cells, extending the in vivo observations from mouse studies (lines 74, 93, 143)

3. IRX3 is also implicated in the development of multiple lineages, including neurogenesis, adipogenesis, and osteogenesis, etc. This broad expression pattern suggests that IRX3 may influence the early specification of diverse stem cell lineages. Therefore, without temporal depletion experiments that selectively target IRX3 during defined windows of cardiac differentiation, it is an overstatement to conclude that IRX3 functions specifically as a molecular barrier to cardiomyocyte maturation.

Response:

We thank the reviewer for this point. We agree that IRX3 is expressed in multiple lineages and that constitutive depletion may influence early stem cell fate more broadly, beyond the cardiac lineage. In our original discussion, we noted the potential for compensatory mechanisms and suggested that temporally controlled modulation of IRX3 would be a valuable strategy. We have now revised the text to more explicitly acknowledge that, without temporal depletion experiments, we cannot exclude lineage-nonspecific effects (lines 391-400).

4. In Figure 5, the authors utilized publicly available ATAC-seq and ChIP-seq data, and their bioinformatic analysis suggested that IRX3 potentially interacted with NKX2.5, TBX5 and GATA4 and regulated genes involved in cardiac differentiation in cardiac progenitor cells. This is an interesting approach; however, a few issues and limitations were noted below.

- This type of in silico approach does not directly test the causal role of IRX3. Thus, the conclusion of this analysis is association rather than causality.

Response:

We agree with the reviewer that our analysis is based on *in silico* integration of publicly available ATAC-seq and ChIP-seq datasets, and therefore demonstrates association rather than definitive causality. We have clarified this limitation in the revised Discussion (lines 395-397). We have modified the text to explicitly state that while these analyses rely on computational predictions, they are aligned with established physical interactions where IRX3 binds to NKX2-5 and TBX5 (Canac et al., Cells, 2022; PMID: 36497174). Therefore, rather than claiming causal proof bioinformatics analyses, we now frame these results as functional evidence that aligns with and supports the physical regulatory model established in the literature.

- The authors mentioned that they used ATAC-seq data from GSE181346, CMs derived from induced pluripotent stem cells (iPSCs) after 5 days of differentiation. This data is scATAC-seq from Day 5 cardiac progenitor cells. However, the method section did not describe how the author analyzed scATAC-seq.

Response:

We thank the reviewer for identifying this omission in the methods section. Indeed, the dataset (GSE181346) is single-cell ATAC-seq. We have now clarified in the revised Methods that we processed this data by aggregating scATAC-seq fragments across all cells to generate a pseudo-bulk accessibility profile. We chose this aggregation strategy to match the resolution of the available transcription factor ChIP-seq datasets, which are bulk measurements. This allows for a direct, integrative comparison of chromatin accessibility and TF binding at the population level. This pseudo-bulk approach is a standard method for integrating single-cell chromatin data with bulk genomic tracks, as established in previous studies (Cusanovich et al., Science, 2015; PMID: 25953818; Granja et al., Nat Biotechnol, 2019; PMID: 31792411). We have updated the "Single-cell ATAC-seq and ChIP-seq (in silico) analysis" section of the Methods to explicitly describe this pseudo-bulk aggregation step (lines 808-810).

- The authors used ChIP-seq data (GSE159411) which was generated on day 6, hiPSC-derived cardiac progenitors. However, it is unclear whether this day 6 genetic information is comparable to day 5 ATAC-seq data and day 4 RNA-seq data. As transcriptional dynamics change rapidly during early differentiation, without clear justification and through marker gene analysis, temporal mismatches could lead to misleading interpretations.

Response:

We acknowledge the reviewer’s valid point regarding the temporal differences between the datasets (Day 4 RNA-seq, Day 5 ATAC-seq, and Day 6 ChIP-seq). We were constrained by the availability of public ChIP-seq data. However, we argue that the differentiation window (Days 4–6) represents the critical phase of cardiac specification where the expression of these core transcription factors is robustly induced. While we acknowledge that transcriptional dynamics are rapid during this transition, the fact that our integrative analysis identified highly specific cardiac gene ontology terms (e.g., cardiac and sarcomere development) rather than noise suggests that the regulatory landscape is sufficiently conserved across these time points to reveal the core IRX3/TF network. We have revised the Discussion to explicitly acknowledge this temporal offset as a limitation. We clarify that this cross-day integration captures robust, high-confidence associations but may miss transient dynamic changes occurring between days (lines 397-402).

- The original study that performed ChIP-seq of NKX2.5, GATA4, and TBX5, also conducted ChIP-seq for H3K27ac, H3K4me3, H3K4me1, H3k27me3 and MED1. It is unclear why the author chose these data over ATAC-seq data.

Response:

We agree that histone marks (e.g., H3K27ac) provide valuable functional context. However, we prioritized scATAC-seq for this analysis for two key reasons. First, our objective was de novo motif discovery. scATAC-seq identifies precise transcription factor footprints, whereas histone marks delineate broader chromatin domains (1–2 kb) that are less specific for motif enrichment. Second, the histone datasets from the original study (GSE159411) were not available for direct download. Given that scATAC-seq provided both a direct and unbiased view of genome-wide accessibility required for motif analysis and was readily accessible, it was the optimal choice for this study.

- Figure 5A, the authors described that “IRX3 binding sequences in open chromatin near each of the three TF loci” -> It is not clear whether IRX3 motifs are located and whether they overlap with the binding sites of NKX2.5, TBX5 or GATA4.

Response:

In the revised Figure 5A, we have made the genomic localization of IRX3 motifs explicit by marking them with distinct colors and enlarged markers. This clearly distinguishes regions where IRX3 motifs are located within accessible chromatin (ATAC-seq peaks) and where they physically overlap with ChIP-seq peaks for NKX2-5, TBX5, and GATA4. To further clarify how these co-localizations were computationally defined, we have expanded the Methods section (Lines 829-836). First, we identified overlaps between ATAC-seq open chromatin regions and IRX3 motifs (derived from ENCODE/chromVARmotifs) to generate a "Motif+ATAC" set. We then assessed intersections between these Motif+ATAC regions and the ChIP-seq peaks for each transcription factor (GATA4, NKX2-5, TBX5) using the findOverlapsOfPeaks function from the *ChIPpeakAnno* package. Only genomic regions containing all three elements (an IRX3 motif, an ATAC-seq open chromatin peak, and a specific TF ChIP-seq peak) were defined as "putative co-bound regulatory sites." The results of this overlap analysis are shown in new S8 Fig, which presents the number and genomic distribution of co-localized peaks for each transcription factor relative to IRX3 motifs.

- Line 243-246: The authors wrote “Motif scanning identified IRX3 binding sequences in open chromatin near each of the three TF loci”. It is not indicated how the authors defined a gene as being regulated by a TF. Is it the proximity of the motif to the gene for Irx3? Please define ‘near’ (e.g., +/- 500 bp?)

Response:

We appreciate the reviewer asking for this clarification. We have used the term "near" in two related but distinct contexts, and we have updated the manuscript to define both explicitly.

First, in our genome-wide analysis, we annotated ATAC-seq peaks based on their distance to the nearest transcription start site (TSS) using the ChIPseeker R package. In this framework, we defined "near" (or promoter-proximal) as being within ±1 kb of the TSS.

Second, in the specific sentence cited by the reviewer ("Motif scanning identified..."), we were referring to the specific open chromatin peaks visualized in Figure 5A. In this context, "identified" implies that the IRX3 motif overlaps (by ≥1 bp) with an ATAC-seq peak that is assigned to that specific TF locus (based on the TSS proximity described above). To remove ambiguity, we have revised the text in the manuscript to be more precise:

Revised Text: “Motif scanning identified putative IRX3 binding sequences within open chromatin regions that were also occupied by these TF (Fig 5A, see S7 Fig for consensus sequence logos).”.

We have also updated the Single-cell ATAC-seq and ChIP-seq (in silico) analysis

section in Methods to explicitly state the criteria for peak annotation (±1 kb from TSS) and motif co-localization (≥1 bp overlap).

**S7 Fig. DNA binding motifs of the transcription factor IRX3.**

- IRX3 motifs were identified in silico. It would be more informative if predicted IRX3 motif sequences were presented.

Response:

We appreciate the suggestion to explicitly visualize the motif sequences. We have now included a new supplementary figure (S7 Fig) displaying the Sequence Logos for the IRX3 binding motifs identified in our analysis. These logos illustrate the consensus binding preferences used to define the IRX3-regulated genomic regions.

- Please deposit the source code used in the bioinformatic analysis.

Response:

We have deposited the complete source code for the bioinformatic analysis in the Zenodo repository. It is permanently accessible via the following Digital Object Identifier (DOI):<https://doi.org/10.5281/zenodo.17791212>. This information has been updated in the "Data and Code Availability" section of the revised manuscript.

Minor points

- Figure 3A: Typo for ACNT2 (supposed to be ACTN2)

Response:

We thank the reviewer for noticing this error. The labeling in Figure 3A has been corrected from "ACNT2" to "ACTN2" in the revised manuscript.

- Figure 4A: It is not clear what gene/protein expression is scaled to. Please consider adding raw data.

Response:

As detailed in our previous responses, we have removed the Western blot data due to antibody cross-reactivity. Therefore, Figure 4A now exclusively displays gene expression data. The Y-axis label has been updated to read “Log2 Normalized Intensity", which corresponds to the microarray signal for the *IRX3* gene following standard processing, normalization, and log transformation.

- Figure S2A: In the flow cytometry, the cell population positive for certain undifferentiated markers may not be high enough (aim for 90-95% positive?).

Response below

- Figure S2C and S4F: No details in the figure caption or legend to describe what the coloured boxes show in the H&E teratoma images.

Response to reviewer comments on Figure S2A/C and S4F:

We appreciate the reviewer’s close examination of the hiPSC characterization data. Regarding previous Figure S2A, while we acknowledge that flow cytometry indicated ~80% positivity for pluripotency markers, we note that such heterogeneity is expected in dynamic stem cell cultures, particularly in hiPSC lines which are known to exhibit higher transcriptional and phenotypic variability compared to hESCs (Narsinh et al., J Clin Invest, 2011; PMID: 21317531). Furthermore, the International Stem Cell Initiative has reported that marker expression levels can fluctuate based on culture density and passage number without compromising the line's developmental potential (Nat Biotechnol, 2007; PMID: 17572666).

Therefore, to strictly validate the quality of our lines, we relied on the gold standard of functional differentiation. As demonstrated in the teratoma assays (now S3 Fig), these lines successfully generated derivatives of all three germ layers *in vivo*, providing definitive evidence of their pluripotency despite the marker variability.

Additionally, as requested, we have revised the legends for S3 Fig to explicitly define the colored boxes, clarifying which histological structures correspond to the ectoderm, mesoderm, and endoderm lineages.

- Figure S3A-D: It only showed the data of clone 1.1.

Response:

We acknowledge that the transcriptional profiling in previous S3 Fig (new S4 Fig) focuses on clone 1.1. To address the concern regarding clonal consistency, we have added new functional data to Figure 1, characterizing the calcium handling properties of both clones 1.1 and 1.2. Consistent with our findings in clone 2, these lines exhibit the same physiological maturation profile (increased transient amplitude and faster decay kinetics). This confirms that while the marker expression shown in S3 is derived from clone 1.1, the functional maturation phenotype is robust and reproducible across all generated lines.

_________________/ /_________________________/ /_____________________/ /______

**Reviewer #2:**

In this manuscript, the authors hypothesize that invalidation of IRX3 expression improves the maturation of cardiomyocytes differentiated from induced pluripotent stem cells.

To do this, they used the CRISPR-Cas9 technique to generate hIPS cells deficient in IRX3.
They then studied various parameters defined as representative of the level of cardiomyocyte maturation in cardiomyocytes differentiated from these hIPSCs.|
In a second part fully In Silico part of the study, they looked at the interactions of IRX3 with transcription factors NKX2-5, GATA4 and TBX5 and question the putative role of these 4 TFs on expression regulation of early cardiac differentiation of hIPSCs, specifically trying to dissect the involvement of IRX3 and the impact of reduction of tits expression.

While the hypothesis is interesting, this manuscript does not fully demonstrate the initial hypothesis because:
(1) some methodological issues exist regarding IRX-/- cells generation and also some of the experiment to demonstrate enhanced maturation
(2) Interpretation of some of the experimental data is not completely convincing and, to my opinion, presented data sometimes lead to divergent interpretations.

Response: We sincerely thank Reviewer #2 for recognizing the interest of our central hypothesis and for their rigorous and insightful critique of our manuscript. We particularly appreciate the reviewer's detailed assessment of the genetic validation of our IRX3-KO lines and the interpretation of the *in silico* regulatory network.

We have taken the reviewer’s concerns regarding the technical demonstration of our hypothesis very seriously and have implemented significant revisions to address the two main points raised:

1. **Methodological Rigor (Point 1):** To address concerns regarding the generation and validation of our IRX3-/- lines, we have rigorously characterized the specific mutations and clarified their description in the text. Furthermore, we performed high-density SNP array analysis. This new dataset provides definitive proof of the genomic stability and clonal isogenicity of our edited lines, resolving the methodological uncertainties regarding the cell models.
2. **Data Interpretation (Point 2):** We have extensively revised the manuscript to refine our interpretation of the experimental data. Specifically, we have contextualized our *in silico* findings as a functional validation of established cardiac networks, ensuring that all conclusions are strictly supported by the presented evidence.

We believe these revisions fully address the concerns regarding technical soundness and provide a robust dataset supporting our conclusions.

The in silico part is interesting but it seems that most of this is not new and, more importantly, it remains at most hypothesis-generating as no confirmation experiment is performed on the lines used in this study.

Response:

We agree that bioinformatics provides predictive insights that require experimental context. We note that recent network analyses (Canac et al., Cells, 2022; PMID: 36497174) have inferred regulatory interactions between IRX3 and core cardiac transcription factors.

We have revised the manuscript to integrate these prior network-level findings and frame our results as an experimental validation of those predictions.

- We have updated the text (Lines 270-273) to note that our analysis builds upon existing network maps (Canac et al., Cells, 2022; PMID: 36497174), investigating whether these predicted interactions drive the phenotype in our specific biological context (*IRX3*-knockout (KO) hiPSC-CMs).
- Experimental Confirmation: Regarding the need for confirmation, we point out that our Microarray data (Figure 5) serves as a readout of these predictions in our lines. We show that ~40% of the genes predicted to be co-regulated by the IRX3/TF network are significantly differentially expressed in our *IRX3*-KO cells. Hence, our data provide experimental evidence that agrees with the *in silico* model proposed.

Relevant text changes:

Line 270 - “IRX3 is known to physically interact with master cardiac regulators such as TBX5, GATA4, and NKX2-5 during heart development [24,27]. Consistent with these physical associations, large scale network analyses have mapped the broader regulatory landscape connecting these TFs in human cardiogenesis [27].”

Line 347 “These data experimentally validate the role of IRX3 within gene regulatory networks previously predicted to govern hiPSC-CM differentiation [27], supporting a model where IRX3 modulates the phenotype by directly interacting with the core cardiac transcription factor complex [27].”

I also consider that the graphical abstract is an overstatement, as it seems more a hypothetic model as the present study only partially demonstrate the depicted mechanisms.

Response:

We agree with the reviewer that the graphical abstract may have inadvertently overstated the mechanistic links established in this study. To ensure precision and avoid overinterpretation, we have removed the graphical abstract from the revised manuscript.

Major points:

• Generation of pluripotent stem cells induced deleted for IRX3:
How was the target zone chosen on the IRX3 gene?
Where is this zone located on the IRX3 gene, particularly in relation to the functional domains of the protein?

Response:

We have now added a schematic in Fig 1A describing the *IRX3* locus, its DNA binding domain, and the region where both gRNAs (for clones Cl1.1/Cl1.2 or Cl2) target the *IRX3* gene. We chose to design the gRNAs to target the exon 1 of *IRX3*, which is located immediately upstream of the DNA binding domain located in the beginning of exon 2, as can now be observed in Fig 1A. We have revised the text to include this information (line number 98, Results section; line numbers 490 and 506, Materials and Methods section).

Where are located the deletions obtained, in relation to the initial target zone?
Is there really a stop codon as initially designed?

Response:

We have now added in S1 Fig allele descriptions for all mutant alleles, in which we describe the location and sequence of the gRNAs, deletions, insertions, frameshifts, and resulting Premature Termination Codons (PTCs). All mutations correlate with the original target region, as now indicated in S1A Fig. Most of these mutations result in PTCs: Alleles 1 and 2 from *IRX3^Cl1.1^*, allele 1 from clone *IRX3^Cl1.2^*, and alleles 1 and 2 from clone IRX3Cl2 result in frameshifts and consequently PTCs. Allele 2 from clone IRX3Cl1.2 has lost its transcription start site and should not be transcribed; however, we also indicate a possible alternative TSS 483 bp downstream. Consequently, all alleles are predicted to give rise to severely truncated proteins (stopping at approx. aa 56-92 aa instead of 501 aa in the wild type).

The four obtained deletions are different (zone and length) also it seems that the target zome was unique. Can authors explain this result?

Response:

The CRISPR sites for all obtained mutations target almost the same region with a difference of 2 bp from each gRNA. The gRNA used for mutating the clones *IRX3^Cl1.1^* and *IRX3^Cl1.2^* (in the same experiment) is located in the plus strand, while the gRNA used for mutating the clone *IRX3^Cl2^* (another experiment) is located in the minus strand. This information can now be seen in the S1A-C Fig. Although CRISPR/Cas9 genome editing is normally referred to as causing small indels, it has previously been reported that subsequent DNA repair mechanisms can result in large mutations and rearrangements that vary from cell to cell (Kosicki et al., Nat Biotechnol, 2018, PMID:30010673). We have revised the text to include this information (lines 95-110, Results section). Thus, even though we used similar gRNAs to target a similar region from *IRX3*, it is expected that the generated mutations will be different from each other.

Furthermore, the use of a karyotype is not sufficient to identify genomic abnormalities related to the generation of hIPS clones or to the CRISPR technique.
Molecular karyotyping such as with SNP array is necessary to validate the lines

Response:

We agree with the reviewer that standard karyotyping has limited resolution for detecting sub-chromosomal abnormalities potentially induced by CRISPR/Cas9 editing.

To address this, we have now performed high-density SNP array analysis using the Axiom™ Human Genotyping Research Array (containing ~870,000 genome-wide markers) to validate the genomic integrity of our lines.

We assessed both the chromosomal stability (Loss of Heterozygosity/CNV burden) and the genetic identity (SNV concordance) of the edited clones compared to their isogenic parents. As shown in the newly added S5 Table (reproduced below), the edited clones displayed a genomic profile highly consistent with the parental lines.

The Loss of Heterozygosity (LOH) percentage remained low and stable. For example, Clone 2 exhibited an LOH rate of 4.29%, identical to its parental control (4.29%), confirming that the editing process did not induce chromosomal instability. In addition, genome-wide SNV concordance between edited clones and parental lines was >98.9%, ruling out cross-contamination. In contrast, unrelated controls showed only ~72% concordance.

S1 Table: Genomic Integrity, Quality Control, and Isogenicity Analysis

(This table has been added to the revised manuscript.)

*Note: MAPD (Median Absolute Pairwise Difference) ≤ 0.30 indicates high-quality hybridization. LOH (Loss of Heterozygosity) values are stable between clones and parents. SNV (Single Nucleotide Variant) concordance >98% confirms clonal isogenicity.*

This molecular validation confirms that our *IRX3*-KO clones are isogenic to the parental iPSCs and have preserved their genomic integrity throughout the editing process.

Also, it is not clear from panel S4C if IRX3-/-cl2 line if both IRX3 alleles have been modified. Panel S4B also suggest modification of a single allele.

Response:

We have addressed the reviewer's concern regarding the zygosity of the mutations. We confirm that both *IRX3* alleles are modified in all clones, including *IRX3^cl2-/-^*.

Previous Western blot data (previous Figure S4B) showed a residual band at the Wild-Type size suggesting the presence of a remaining unmodified allele, but we have now provided definitive genetic evidence to clarify this:

1. **Confirmation of Biallelic Modification (Subcloning):** To categorically determine the genotype of each allele, we performed a subcloning analysis. We generated individual subclones from the edited lines (*IRX3^Cl1.1^*, *IRX3^Cl1.2^*, and *IRX3^Cl2^*) and sequenced them independently. As shown in new S1 Fig, this analysis confirmed that no Wild-Type alleles are present. For clone 2, sequencing revealed homozygous frameshift mutations, resulting in early Premature Termination Codons (PTC) on both alleles. For clone 1, sequencing confirmed that all subclones carry either loss of the transcription start site (*IRX3^cl1.2^* allele 2) or transheterozygous frameshift mutations resulting in early PTCs.
2. **"Single Allele" Artifact:** The reason the Western blot suggested a "single allele modification" (a persistent band at ~52 kDa) is likely due to antibody cross-reactivity. The mutations in clone 2 are predicted to generate a severely truncated peptide (<15 kDa, stopping near 56 aa), which is significantly smaller than the Wild-Type size (~52 kDa, 501 aa). Therefore, the band observed in the previous figure could not be the product of a "remaining Wild-Type allele" nor the mutant protein. Additionally, as shown in the Figure R1 (Please refer to Figure R1 presented in the response to Reviewer #1, Comment #1 above), other IRX family members (IRX1, IRX2, IRX4, IRX5) share considerable homology and molecular weights (50–54 kDa), providing the likely source of this non-specific cross-reactivity signal.

Given that the Western blot data was likely artifactual and contradicted the definitive sequencing results (which demonstrate biallelic disruption), we have removed the Western blot to prevent further confusion. We rely on the rigorous subcloning and sequencing data (new S1 Fig) to demonstrate that clone 2 is a true biallelic null.

In panel 1C, staining of TnI seems unusual with most of the protein at the membrane for all 3 clones. Can author provide a higher magnification picture (as in fig 2J for the other lines)?

Response:

We thank the reviewer for this observation. We agree that at lower magnification (10×), TnI staining may appear enriched at the cell membrane due to the density of the monolayer. To clarify this, we utilized high-magnification imaging (63×) to resolve the subcellular distribution. As shown in the representative image below (Figure R2), this reveals the expected filamentous and sarcomeric organization of TnI throughout the cytosol, confirming proper localization.

Figure R2. Immunofluorescence of WT cardiomyocytes stained with DAPI (blue) and TnI (red). The left panel shows a low-magnification field (10× objective; scale bar = 100 µm), while the right panel shows the highlighted region at higher magnification (63× objective; scale bar = 25 µm).

*(For peer review purposes only).*

Additionally, we have updated Fig. 1 to include zoom-in insets for the clones, which further illustrate the expected sarcomeric pattern. These clarifications do not alter the interpretation or conclusions of the study.

In figure S1:

- full- length protein is expressed whereas deletion was supposed to create a stop codon.
- change in protein expression between -/- and +/+ lines does not correspond to what is expected with the aim of a bi-allelic deletion
- IRX3 protein is clearly present in the cells at day 6 of the differentiation
Therefore it is at least necessary to know if the produced IRX3 protein functionally active in the -/- cells

Response:

We completely agree with the reviewer’s assessment. The observation that a "full-length protein is expressed" despite a genomic "stop codon" is indeed a contradiction. This discrepancy is precisely what led us to re-evaluate the specificity of the antibody signal.

To answer the reviewer’s specific question regarding whether the produced protein is functionally active: No, the mutant protein cannot be functional.

1. **Genetic Evidence of Non-Functionality:** As detailed in our new subcloning analysis (new S1 Fig), all alleles contain Premature Termination Codons (PTCs) located upstream of the Homeodomain (DNA Binding Domain). Even if these truncated transcripts escape Nonsense-Mediated Decay and are translated, the resulting peptides (<15 kDa) would lack the structural capacity to bind DNA or regulate transcription.
2. **The "Full-Length" Band is an Artifact:** The band observed at the full-length size (~52 kDa) in the previous figure is not compatible with the truncated proteins encoded by our mutant alleles (predicted size <15 kDa).
3. **Source of the Signal:** As shown in the Figure R1 (Please refer to Figure R1 presented in the response to Reviewer #1, Comment #1 above), other IRX family members share high sequence homology and molecular weights (50–54 kDa). The persistent signal observed at Day 6 and in the knockout lines is attributable to antibody cross-reactivity likely with these conserved family members, not functional IRX3.

Because the Western blot signal is likely an artifact that contradicts the definitive genetic loss-of-function data, we have removed it from the manuscript to avoid misleading the readers. We rely on the rigorous sequencing data to define the functional null status of the lines.

• Gene expression of IRX3-/- vs IRX3+/+ cells (in figure S3)

Overall the data does not seem very meaningful as TNNI1 is a fetal isoform, as CX43 is anyway supposed to be express in all cardiomyocytes, as Cx40 is more specifically expressed on conduction system, and as SCN5A is key to mature cardiomyocyte contractile activity.
Instead, comparing the relative expression of fetal vs mature forms of some cardiomyocyte key proteins would be more relevant (such as TNNI3 and TNNI1, MYL2 and MYL7, or MYH6 and MYH7). Indeed, it would be interesting to have the expression data for lines derived from cl1 and cl2

Response:

We agree with the reviewer that evaluating the ratio of mature vs. fetal isoforms provides a more relevant assessment of cardiomyocyte maturation than the general markers initially presented. We also agree on the necessity of verifying these profiles across independent clones.

To address this, we performed additional qPCR analysis on clone 2 (now presented in S5 Fig) and expanded our panel to include the specific isoforms and calcium handling proteins.

1. **Isoforms (clone 2):** As requested, we analyzed the expression of mature sarcomeric isoforms. We observed a significant upregulation of both *MYH6* (alpha-MHC) and *MYH7* (beta-MHC) in clone 2 compared to controls, indicating a robust induction of the contractile apparatus. While the induction of Heavy Chain isoforms was more pronounced, *TNNI3* exhibited a trend towards upregulation.
2. **Calcium Handling (SERCA2/RYR2):** To better characterize the functional machinery, we also analyzed key regulators of Excitation-Contraction Coupling. We found that SERCA2 (*ATP2A2*) and *RYR2* (Ryanodine Receptor 2) are both upregulated in Clone 2 (new S5 Fig). While increased *SERCA2* expression provides the molecular basis for the shorter calcium decay duration (improved relaxation) and higher sarcoplasmic reticulum load observed in our physiological assays, *RYR2* expression is consistent with the higher calcium amplitude.
3. **Clonal Consistency (Function):** While the detailed qPCR panel shown in new Figure S5 was conducted on Clone 2, we have ensured that the resulting functional phenotype is reproducible across all clones. As shown in the updated Figure 1, Clones 1.1 and 1.2 exhibit the same physiological hallmarks as Clone 2: a Higher Calcium Amplitude and Max Rise Slope, and Faster Decay Kinetics.

In summary, the new data confirms that *IRX3* depletion drives a molecular program of maturation (upregulation of *MYH6/7*, *SERCA2*, *RYR2*) that translates into a consistent functional phenotype across multiple independent clones.

• Electrical activity

Can authors provides representatives traces of action potential for all clones to help appreciate le level of maturity? For instance, observing an overshoot or a notch would be very important.
Some patch-clamp traces would also be more relevant given the importance of these parameters to assess cell maturation state.

Response:

We appreciate the reviewer’s suggestion to visualize the Action Potential (AP) morphology to better assess the maturity level. Optical voltage recordings using FluoVolt allow us to compare relative action potential morphology and kinetics across conditions. However, voltage-sensitive dyes do not report absolute membrane potential values and therefore cannot directly demonstrate classical electrophysiological features such as the overshoot above 0 mV or the subtle phase-1 notch observed in patch-clamp recordings of mature ventricular or Purkinje cardiomyocytes. Instead, FluoVolt reports relative changes in fluorescence proportional to voltage fluctuations, which reliably capture the upstroke, plateau, and repolarization dynamics of action potentials (Miller et al., Proc Natl Acad Sci USA, 2012; PMID: 22308458; Leyton-Mange et al., Stem Cell Reports, 2014; PMID: 24527390; Takaki, Methods Mol Biol., 2021; PMID: 34302652). While overshoot and notch cannot be unequivocally resolved with this technique, we have now added representative traces for all clones to new S5 Fig to illustrate relative maturity and electrophysiological differences across conditions, and we thank the reviewer for this helpful suggestion, which has strengthened the figure presentation.

Shown in new S5 Fig

• Figure 4

It seems critical to assess IRX expression in all the IRX3 +/+ and IRX3-/- lines used in this paper as well, especially at day 4 Could authors please provide the microarray expression data for IRX3, IRX5, GATA4, NKX2-5?

Response:

We thank the reviewer for this request. For transparency, we have extracted the normalized expression values for *IRX3, IRX5, GATA4,* and *NKX2-5* from the microarray dataset (n=2) as requested (Table R1 below).

**Table R1: Microarray Expression Values (Day 4)**

| **SYMBOL** | **IRX3 +/+ Rep1 (Exp)** | **IRX3 +/+ Rep2 (Exp)** | **IRX3 -/- Rep1 (Exp)** | **IRX3 -/- Rep2 (Exp)** | **logFC** | **adj.P.Val** |
| --- | --- | --- | --- | --- | --- | --- |
| TBX5 | 2.60 | 3.32 | 6.63 | 4.21 | 2.46 | 0.02 |
| GATA4 | 8.04 | 8.19 | 8.90 | 8.61 | 0.64 | 0.40 |
| NKX2-5 | 3.49 | 3.08 | 2.73 | 2.54 | -0.64 | 0.30 |
| IRX3 | 7.81 | 7.77 | 8.13 | 8.29 | 0.41 | 0.39 |
| IRX5 | 5.57 | 6.28 | 5.55 | 5.13 | -0.59 | 0.44 |

*(For peer review purposes only).*

As shown in the table, while *TBX5* showed an upregulation trend consistent with our hypothesis, the microarray data for *GATA4* and *NKX2-5* showed variability, which is a limitation of the small sample size in this specific dataset. Therefore, to address the reviewer’s query, we have utilized RT-qPCR in the revised manuscript, which allows for robust statistical validation across multiple independent clones.

We found that *GATA4* and *TBX5* were consistently significantly upregulated on day 4 across multiple *IRX3*-KO clones (new S6 Fig), confirming that the loss of *IRX3* accelerates the mesoderm-to-cardiac transition. The regulation of *NKX2-5* appeared more complex at the transcript level on day 4. However, our Immunofluorescence data on day 6 showed protein upregulation. This temporal delay suggests that *NKX2-5* stabilization may depend on the prior upregulation of its partners (*GATA4/TBX5*). This aligns with recent network analyses demonstrating that *NKX2-5* expression is highly sensitive to the combinatorial stoichiometry of other core transcription factors (*Canac et al., Cells, 2022, PMID: 36497174*).

• Figure 5

Can the authors precisely indicated which samples were used from GSE181346 and GSE159411 data sets? How were, if any, the replicates handled? It seems that ATAC-seq data are from single-seq data, how was data from each single cell consolidated into a unique result? What was the observed variability?

Response:

We thank the reviewer for ensuring the reproducibility and clarity of our bioinformatic workflow.

**1. Sample Identification:** We have updated the "Data and Code Availability" section in the revised manuscript to list the specific Gene Expression Omnibus (GEO) sample accession numbers (GSM IDs) used from both datasets (GSE181346 and GSE159411).

**2. scATAC-seq Consolidation & Variability (GSE181346):** The reviewer is correct that this dataset is single-cell ATAC-seq. We did not analyze individual single-cell replicates separately. Instead, we aggregated fragments across all quality-controlled cardiac progenitor cells to generate a pseudo-bulk chromatin accessibility profile. This approach mathematically sums fragments to reconstruct a population-level consensus landscape. In this specific study design, cell-to-cell variability was intentionally collapsed (averaged) rather than quantified. Our objective was to perform an integrative overlap analysis with publicly available ChIP-seq datasets, which are inherently bulk data. To ensure valid cross-dataset integration, it was necessary to match the resolution of the scATAC-seq data to the bulk resolution of the TF binding maps. Previous studies have demonstrated that this pseudo-bulk aggregation produces highly reproducible profiles comparable to standard bulk ATAC-seq (Cusanovich et al., Science, 2015; PMID: 25953818; Granja et al., Nat Biotechnol, 2019; PMID: 31792411).

Therefore, while we did not assess single-cell heterogeneity, this pseudo-bulk approach allowed us to identify high-confidence, shared regulatory regions essential for the population-level motif enrichment analysis presented in Figure 5. We have clarified this "pseudo-bulk" methodology in the "Single-cell ATAC-seq and ChIP-seq (in silico) analysis" section of the Methods.

**3. ChIP-seq Replicate Handling (GSE159411):** For the transcription factors (NKX2-5, GATA4, TBX5), we obtained the peak files for available biological replicates. These replicates were merged using the ChIPseeker package to generate a consensus set of binding sites for each factor, ensuring that our overlap analysis captured the comprehensive binding landscape.

Minor points:

• In panel S4C, it seems that legends are misplaced.

Response:

We apologize for the formatting error in the previous version of the manuscript. However, in response to the reviewer’s Major Point regarding the characterization of the alleles, Panel S4C has been replaced by the new, comprehensive S1A-D Fig. This new figure provides a high-resolution alignment of all alleles derived from the subcloning analysis, with clear and corrected annotations for each clone and subclone.

• I understand that panel 1D and 1E have been obtained by counting cells from panel 1C images, is it true? (I have not been able to find this information). If yes, % of positive cells in panel 1D/E does not match with what can be observed in 1C especially for MLC2v

Response:

We thank the reviewer for this observation. The reviewer is correct that panels 1D and 1E were generated by quantifying the images represented in panel 1C. Upon re-examination of the raw images, we identified technical inconsistencies in the MLC2v staining efficiency across replicates that compromised the image-based quantification. To avoid presenting potentially misleading results, we have removed the MLC2v IF data from the revised manuscript.

*Note:* The ventricular specification and maturation of these cells are alternatively and robustly supported by the qPCR analysis (new S5 Fig), which shows a significant upregulation of the ventricular isoform MYH7 (beta-MHC) in the *IRX3*-KO clones.

Panel 1D (TnI quantification) has been retained and clarified in the figure legend as follows:

**New Fig. 1. IRX3 depletion increases hiPSC-derived cardiomyocyte differentiation.**

**(A–B)** Schematic of CRISPR/Cas9-mediated generation of *IRX3* KO hiPSC clones (*IRX3^Cl1.1-/-^* and *IRX3^Cl1.2-/-^*) and overview of the differentiation protocol used to generate hiPSC-CMs.

**(C)** Representative immunofluorescence images of TnI (red) in *IRX3^Cl1+/+^, IRX3^Cl1.1-/-^* and *IRX3^Cl1.2-/-^* hiPSC-CMs at day 30. Nuclei were counterstained with DAPI (blue). Insets show magnified views of the dashed regions. Scale = 100µm.

**(D)** Quantification of TnI-positive cells (n = 2,000–4,000 cells per group across 4 independent replicates). Data were obtained by analyzing images such as those in panel 1C. Data are presented as mean ± SD. One-way ANOVA; *P < 0.05 vs. *IRX3^Cl1+/+^.*

• Mitochondrial organization

Can authors provide references indicating that parameters measured in figures 2 K-N are indeed related to a better maturation of hIPS derived cardiomyocytes

Alternatively, functional data showing an effective metabolic switch would be more convincing.

Response:

We thank the reviewer for this comment. We agree that the link between mitochondrial organization and cardiomyocyte maturation required clearer referencing. We have revised lines 361-364 of the Discussion to include specific citations supporting that the parameters we measured—specifically mitochondrial aspect ratio (elongation) and network distribution—are established structural hallmarks of the metabolic switch from glycolysis to oxidative phosphorylation (Karbassi et al., *Nat Rev Cardiol*, 2020; PMID: 32015528; Feyen et al., Cell Rep., 2020; PMID: 32697997; Dai et al., *Circ Res*, 2017; PMID: 28421116).

Regarding the suggestion for metabolic flux assays (e.g., Seahorse), we agree that this would provide direct functional confirmation. However, we emphasize that the structural maturation of the mitochondrial network we observed correlates strongly with the functional maturation of excitation-contraction coupling (enhanced Calcium transient amplitudes and SERCA2 load) demonstrated in our physiological assays. Given that mitochondrial remodeling is a prerequisite for sustaining these high-energy calcium fluxes, our data supports a model of coordinated structural and functional maturation.

• Figure 3 It seem difficult to obtain accurate data on cell area and sarcomere organization if the analysis was performed when cell overlap as shown in 3A and when there is no staining for cell membrane. Can the authors comment on that? How was done the quantification in 3F. Has this methodology already been validated?

Response:

We thank the reviewer for this important observation. We acknowledge that accurately segmenting cells without membrane staining can be challenging, especially in regions of cell overlap. We initially attempted to use membrane markers (CellMask and WGA), but found that they did not yield sufficient separation in these dense cardiomyocyte clusters to support reliable automated counting

To ensure accuracy and eliminate segmentation artifacts, we restricted our analysis to cardiomyocytes located at the periphery of clusters, where cell boundaries were clearly defined by the cytosolic TnI and alpha-actinin signal. To minimize bias, cell delineation was performed manually by a blinded observer rather than relying on automated algorithms that might misinterpret overlapping regions. We have updated the Methods section to explicitly state this approach. The revised text (line 733) now reads:

“…isolated cells at the periphery were segmented manually.”

Regarding the quantification methodology in Figure 3F, we have expanded the “Confocal microscopy” section in the Methods to detail the analysis workflow. The revised text now reads (lines 698-702):

“For Connexin-43 quantification, CX43-positive and TnI-positive areas were segmented using the ‘Threshold’ module. The ratio of CX43 area to cardiomyocyte area was then calculated and multiplied by 100 (i.e., CX43 area / TnI-positive area × 100). The resulting values were converted to fold change to facilitate comparison across groups.”

This quantification approach—normalizing CX43-positive area by cardiomyocyte area—has been previously validated in studies using hiPSC-derived cardiomyocytes (William J Kowalski, Front Cell Dev Biol, 2022; PMID: 35359438).

• Figure 4

For panel E, I am not sure that the quantification method used for GATA4 and NKX2-5 is valid. Could authors provide a reference publication for this methodology?
Also, I suggest % of Ki67+ cells should calculated on GATA4 or NKX2-5+ to indicate that this enhanced proliferation is related to cardiac lineage cells rather than another non relevant cell population

Response:

We appreciate the reviewer’s request to clarify our quantification methodology. Regarding the quantification of GATA4 and NKX2-5 (Figure 4E), to quantify transcription factor expression, we employed immunofluorescence followed by nuclear segmentation and analysis of mean fluorescence intensity (MFI). This approach allows for a precise, quantitative assessment of protein abundance per nucleus. This methodology aligns with established quantitative standards in the field; for instance, Khan [et al](http://et.al). (J Tissue Eng Regen Med, 2022; PMID: 34726328) quantified GATA4 expression using "average nuclear integrated intensity" to assess cardiomyocyte differentiation. In our study, we reported Mean Fluorescence Intensity (MFI), which is mathematically related to Integrated Density (Integrated Density = Mean Intensity × Nuclear Area). We chose MFI over "percentage positive" for a specific biological reason: our differentiation protocol is highly efficient, resulting in GATA4 and NKX2-5 expression in nearly 100% of nuclei in both WT and KO lines. A binary presence/absence metric would therefore fail to capture any phenotypic difference. By quantifying MFI, we detected the graded increase in protein abundance per nucleus, which reflects the enhanced maturation state of the *IRX3*-deficient cells. Furthermore, as we observed no significant difference in nuclear size between groups, MFI and Integrated Density provide equivalent biological conclusions regarding transcription factor abundance.

Regarding the Ki67 analysis, we agree that restricting Ki67 quantification specifically to GATA4+ or NKX2-5+ nuclei is an excellent way to confirm lineage-specific proliferation. However, in this specific experimental series, Ki67 and the cardiac transcription factors were assessed in separate staining panels to maximize channel availability for other markers, preventing retrospective co-localization analysis. Crucially, however, the cultures used for this analysis were highly enriched for cardiomyocytes. As demonstrated in Figure 1D, our protocol yields a population that is predominantly cTnT+ (>80%). Given this high purity, the significant increase in Ki67 signal observed in the *IRX3*-KO lines is overwhelmingly attributable to the cardiomyocyte population rather than a minor non-cardiac contaminant.

• Panel 5B: It seems that numbers in black correspond to typos

Response:

We thank the reviewer for spotting this error. We have corrected the typographical annotations in new Figure 5B in the revised manuscript.

• Panel S6A: It would be interesting to define how many gene containing IRX3 binding sequences in open chromatin does not contain binding sequence for any of the 3 other genes (NKX2-5, GATA4 or TBX5). This would give an indication of the importance of the co-regulation of these 4 TFs.

Response:

We thank the reviewer for this insightful suggestion. To address this, we have replaced the Venn diagram with an UpSet plot (Figure 5A), quantified these distinct populations, and included these detailed analyses in a revised S8 Fig.

From the total universe of 26,135 unique genes associated with IRX3 occupancy, we identified 18,979 genes ('IRX3-Solo') that lack binding sites for NKX2-5, GATA4, or TBX5. In contrast, 7,156 genes are co-occupied by at least one of these core factors.

Importantly, our functional enrichment analysis revealed a striking dichotomy between these groups. Notably, this was not due to gene quality, since both the 'Solo' and 'Co-occupied' populations contain a similar proportion (~60%) of protein-coding genes. However, while the large 'IRX3-Solo' population is enriched for non-cardiac lineages (e.g., sensory perception, immune response), the cardiac-specific structural terms (e.g., sarcomere organization, muscle cell differentiation) are exclusively driven by the co-occupied subset.
