# Supplementary material for: IRX3 depletion promotes early cardiac commitment of hiPSC-Derived Cardiomyocytes
Source: PLoS One. 2026 Jun 16;21(6):e0351704. doi: 10.1371/journal.pone.0351704 (PMC13271448; doi:10.1371/journal.pone.0351704)
Supplement: Peer review history

# Author comment: Author response to Decision Letter 0

**DOI:** 10.1371/journal.pone.0351704.r001

**Transfer Alert**

This paper was transferred from another journal. As a result, its full editorial history (including decision letters, peer reviews and author responses) may not be present.

25 Jun 2025

# Peer review: Decision Letter 0

**DOI:** 10.1371/journal.pone.0351704.r002

**Authors:** Michael Schubert

24 Sep 2025

PONE-D-25-27545

IRX3 Depletion Promotes Early Cardiac Commitment of hiPSC-Derived Cardiomyocytes

PLOS ONE

Dear Dr. Krieger,

Thank you for submitting your manuscript to PLOS ONE. After careful consideration, we feel that it has merit but does not fully meet PLOS ONE’s publication criteria as it currently stands. Therefore, we invite you to submit a revised version of the manuscript that comprehensively addresses the points raised during the review process. Note that both reviewers have raised significant concerns about both methodology and interpretation of the obtained results, which need to be alleviated to render the submission acceptable for publication in PLOS ONE.

Please submit your revised manuscript by Nov 08 2025 11:59PM. If you will need more time than this to complete your revisions, please reply to this message or contact the journal office at plosone@plos.org. When you’re ready to submit your revision, log on to https://www.editorialmanager.com/pone/ and select the 'Submissions Needing Revision' folder to locate your manuscript file.

Please include the following items when submitting your revised manuscript:

- A rebuttal letter that responds to each point raised by the academic editor and reviewer(s). You should upload this letter as a separate file labeled 'Response to Reviewers'.
- A marked-up copy of your manuscript that highlights changes made to the original version. You should upload this as a separate file labeled 'Revised Manuscript with Track Changes'.
- An unmarked version of your revised paper without tracked changes. You should upload this as a separate file labeled 'Manuscript'.

If you would like to make changes to your financial disclosure, please include your updated statement in your cover letter. Guidelines for resubmitting your figure files are available below the reviewer comments at the end of this letter.

If applicable, we recommend that you deposit your laboratory protocols in protocols.io to enhance the reproducibility of your results. Protocols.io assigns your protocol its own identifier (DOI) so that it can be cited independently in the future. For instructions see: https://journals.plos.org/plosone/s/submission-guidelines#loc-laboratory-protocols. Additionally, PLOS ONE offers an option for publishing peer-reviewed Lab Protocol articles, which describe protocols hosted on protocols.io. Read more information on sharing protocols at https://plos.org/protocols?utm_medium=editorial-email&utm_source=authorletters&utm_campaign=protocols.

We look forward to receiving your revised manuscript.

Kind regards,

Michael Schubert

Academic Editor

PLOS ONE

Journal Requirements:

When submitting your revision, we need you to address these additional requirements.

1.Please ensure that your manuscript meets PLOS ONE's style requirements, including those for file naming. The PLOS ONE style templates can be found at

https://journals.plos.org/plosone/s/file?id=wjVg/PLOSOne_formatting_sample_main_body.pdf and

https://journals.plos.org/plosone/s/file?id=ba62/PLOSOne_formatting_sample_title_authors_affiliations.pdf

2. When you submit your revised manuscript, please correct the dates of the cell line ethical approval in our online submission portal. You state that ethics approval 0055/11 was granted on 20 September 2019, while in the manuscript and the ethics document itself, the date is stated to be 21 March 2012. Thank you.

3. Please note that funding information should not appear in any section or other areas of your manuscript. We will only publish funding information present in the Funding Statement section of the online submission form. Please remove any funding-related text from the manuscript.

4. We note that the grant information you provided in the ‘Funding Information’ and ‘Financial Disclosure’ sections do not match.

When you resubmit, please ensure that you provide the correct grant numbers for the awards you received for your study in the ‘Funding Information’ section.

5. Thank you for stating the following financial disclosure:

“This work was supported by grants from the Fundação de Amparo à Pesquisa do Estado de São Paulo (FAPESP) [INCT - 20214/50889-7 to JEK, 2013/17368-0 to JEK, 2015/50216-5 to JEK, 2017/05829-4 to ARS, 2021/14876-1 to NDF, 2023/05709-0 to NDF] and from the Conselho Nacional de Pesquisa (CNPq) [INCT - 465586/2014-7 to JEK, 309179/2013-0 to JEK, 442643/2020-9 to JEK].”

Please state what role the funders took in the study.  If the funders had no role, please state: "The funders had no role in study design, data collection and analysis, decision to publish, or preparation of the manuscript."

If this statement is not correct you must amend it as needed.

Please include this amended Role of Funder statement in your cover letter; we will change the online submission form on your behalf.

6. Your ethics statement should only appear in the Methods section of your manuscript. If your ethics statement is written in any section besides the Methods, please move it to the Methods section and delete it from any other section. Please ensure that your ethics statement is included in your manuscript, as the ethics statement entered into the online submission form will not be published alongside your manuscript.

7. Please ensure that you refer to Figure 6 in your text as, if accepted, production will need this reference to link the reader to the figure.

8. Please upload a copy of Figure 7, to which you refer in your text on page 32. If the figure is no longer to be included as part of the submission please remove all reference to it within the text.

9. PLOS ONE now requires that authors provide the original uncropped and unadjusted images underlying all blot or gel results reported in a submission’s figures or Supporting Information files. This policy and the journal’s other requirements for blot/gel reporting and figure preparation are described in detail at https://journals.plos.org/plosone/s/figures#loc-blot-and-gel-reporting-requirements and https://journals.plos.org/plosone/s/figures#loc-preparing-figures-from-image-files. When you submit your revised manuscript, please ensure that your figures adhere fully to these guidelines and provide the original underlying images for all blot or gel data reported in your submission. See the following link for instructions on providing the original image data: https://journals.plos.org/plosone/s/figures#loc-original-images-for-blots-and-gels.

In your cover letter, please note whether your blot/gel image data are in Supporting Information or posted at a public data repository, provide the repository URL if relevant, and provide specific details as to which raw blot/gel images, if any, are not available. Email us at plosone@plos.org if you have any questions.

10. If the reviewer comments include a recommendation to cite specific previously published works, please review and evaluate these publications to determine whether they are relevant and should be cited. There is no requirement to cite these works unless the editor has indicated otherwise.

[Note: HTML markup is below. Please do not edit.]

Reviewers' comments:

Reviewer's Responses to Questions

**Comments to the Author**

1. Is the manuscript technically sound, and do the data support the conclusions?

The manuscript must describe a technically sound piece of scientific research with data that supports the conclusions. Experiments must have been conducted rigorously, with appropriate controls, replication, and sample sizes. The conclusions must be drawn appropriately based on the data presented.

Reviewer #1: Yes

Reviewer #2: Partly

**********

2. Has the statistical analysis been performed appropriately and rigorously?

Reviewer #1: Yes

Reviewer #2: Yes

**********

3. Have the authors made all data underlying the findings in their manuscript fully available?

The PLOS Data policy requires authors to make all data underlying the findings described in their manuscript fully available without restriction, with rare exception (please refer to the Data Availability Statement in the manuscript PDF file). The data should be provided as part of the manuscript or its supporting information, or deposited to a public repository. For example, in addition to summary statistics, the data points behind means, medians and variance measures should be available. If there are restrictions on publicly sharing data—e.g. participant privacy or use of data from a third party—those must be specified.

Reviewer #1: Yes

Reviewer #2: No

**********

4. Is the manuscript presented in an intelligible fashion and written in standard English?

PLOS ONE does not copyedit accepted manuscripts, so the language in submitted articles must be clear, correct, and unambiguous. Any typographical or grammatical errors should be corrected at revision, so please note any specific errors here.

Reviewer #1: Yes

Reviewer #2: Yes

**********

5. Review Comments to the Author

Please use the space provided to explain your answers to the questions above. You may also include additional comments for the author, including concerns about dual publication, research ethics, or publication ethics. (Please upload your review as an attachment if it exceeds 20,000 characters)

Reviewer #1: This study by da Silva et al. tests whether suppressing IRX3 enhances human iPSC-cardiomyocyte differentiation. Using CRISPR/Cas9 IRX3-null hiPSC lines, the authors show that IRX3 depletion (knockdown and knockout) increased cardiomyocyte yield, higher expression of ventricular markers (TNNI1, MLC2V), elevated CX43, and reduced SCN5A. Functional assessments indicate that IRX3-deficient cells exhibit improved calcium handling, shorter APDs, greater contraction/relaxation velocities, broader mitochondrial networks, and more ordered sarcomeres. Temporal analysis shows IRX3 expression peaks at day 4 of iPSC-CM differentiation. Its loss upregulates early cardiogenic TFs (GATA4, NKX2-5, TBX5, MEF2, HAND), expands progenitors, and activates Wnt-related programs. The authors utilized publicly available ATAC and ChIP data for integrative analysis and suggested IRX3 co-regulates target genes with GATA4/NKX2-5/TBX5. Together, the authors propose that IRX3 acts as a brake on early cardiogenesis.

Overall, this manuscript presented potentially impactful findings. However, there are several concerns regarding citation accuracy, strength of genetic perturbation, and interpretation of integrative in silico analyses. Clarification and further validation are needed to strengthen the conclusions.

Major comments:

1. While the authors targeted IRX3 for its depletion in hiPSC cells, mRNA and protein expression data in all three different clones showed moderately decreased IRX3 protein level (~40%). Given that IRX3 is not known to be haploinsufficient, it remains unclear whether the observed phenotypes are directly attributable to functional IRX3 loss. The authors should clarify and discuss this limitation.

2. The authors cited previous papers investigating Irx3 and Irx5, such as Development (2012) and Scientific Reports (2016) and mentioned as follows:

Line 74: Given the repressive role of Irx3 in the differentiation of working CMs in mouse..

Line 91: Given the established role of Irx3 in mouse CM differentiation and maturation in vivo..

Line 125: IRX3 depletion promotes CM differentiation and may bias subtype specification toward ventricular working CMs, consistent with in vivo mouse data

However, previous studies have primarily focused on the role of this factor in the development and maturation of the ventricular conduction system, rather than its repressive effect on working CM differentiation. Therefore, these citations seem not to be correctly used to build rationale and justify their experiments and findings on Irx3, respectively. It should be revised accordingly.

3. IRX3 is also implicated in the development of multiple lineages, including neurogenesis, adipogenesis, and osteogenesis, etc. This broad expression pattern suggests that IRX3 may influence the early specification of diverse stem cell lineages. Therefore, without temporal depletion experiments that selectively target IRX3 during defined windows of cardiac differentiation, it is an overstatement to conclude that IRX3 functions specifically as a molecular barrier to cardiomyocyte maturation.

4. In Figure 5, the authors utilized publicly available ATAC-seq and ChIP-seq data, and their bioinformatic analysis suggested that IRX3 potentially interacted with NKX2.5, TBX5 and GATA4 and regulated genes involved in cardiac differentiation in cardiac progenitor cells. This is an interesting approach; however, a few issues and limitations were noted below.

- This type of in silico approach does not directly test the causal role of IRX3. Thus, the conclusion of this analysis is association rather than causality.

- The authors mentioned that they used ATAC-seq data from GSE181346, CMs derived from induced pluripotent stem cells (iPSCs) after 5 days of differentiation. This data is scATAC-seq from Day 5 cardiac progenitor cells. However, the method section did not describe how the author analyzed scATAC-seq.

- The authors used ChIP-seq data (GSE159411) which was generated on day 6, hiPSC-derived cardiac progenitors. However, it is unclear whether this day 6 genetic information is comparable to day 5 ATAC-seq data and day 4 RNA-seq data. As transcriptional dynamics change rapidly during early differentiation, without clear justification and through marker gene analysis, temporal mismatches could lead to misleading interpretations.

- The original study that performed ChIP-seq of NKX2.5, GATA4, and TBX5, also conducted ChIP-seq for H3K27ac, H3K4me3, H3K4me1, H3k27me3 and MED1. It is unclear why the author chose these data over ATAC-seq data.

- Figure 5A, the authors described that “IRX3 binding sequences in open chromatin near each of the three TF loci” -> It is not clear whether IRX3 motifs are located and whether they overlap with the binding sites of NKX2.5, TBX5 or GATA4.

- Line 243-246: The authors wrote “Motif scanning identified IRX3 binding sequences in open chromatin near each of the three TF loci”. It is not indicated how the authors defined a gene as being regulated by a TF. Is it the proximity of the motif to the gene for Irx3? Please define ‘near’ (e.g., +/- 500 bp?)

- IRX3 motifs were identified in silico. It would be more informative if predicted IRX3 motif sequences were presented.

- Please deposit the source code used in the bioinformatic analysis.

Minor points

- Figure 3A: Typo for ACNT2 (supposed to be ACTN2)

- Figure 4A: It is not clear what gene/protein expression is scaled to. Please consider adding raw data.

- Figure S2A: In the flow cytometry, the cell population positive for certain undifferentiated markers may not be high enough (aim for 90-95% positive?).

- Figure S2C and S4F: No details in the figure caption or legend to describe what the coloured boxes show in the H&E teratoma images.

- Figure S3A-D: It only showed the data of clone 1.1.

Reviewer #2: In this manuscript, the authors hypothesize that invalidation of IRX3 expression improves the maturation of cardiomyocytes differentiated from induced pluripotent stem cells.

To do this, they used the CRISPR-Cas9 technique to generate hIPS cells deficient in IRX3.

They then studied various parameters defined as representative of the level of cardiomyocyte maturation in cardiomyocytes differentiated from these hIPSCs.

In a second part fully In Silico part of the study, they looked at the interactions of IRX3 with transcription factors NKX2-5, GATA4 and TBX5 and question the putative role of these 4 TFs on expression regulation of early cardiac differentiation of hIPSCs, specifically trying to dissect the involvement of IRX3 and the impact of reduction of tits expression.

While the hypothesis is interesting, this manuscript does not fully demonstrate the initial hypothesis because:

(1) some methodological issues exist regarding IRX-/- cells generation and also some of the experiment to demonstrate enhanced maturation

(2) Interpretation of some of the experimental data is not completely convincing and, to my opinion, presented data sometimes lead to divergent interpretations.

The in silico part in interesting but it seems that most of this is not new and, more importantly, it remains at most hypothesis-generating as no confirmation experiment is performed on the lines used in this study.

I also consider that the graphical abstract is an overstatement, as it seems more a hypothetic model as the present study only partially demonstrate the depicted mechanisms.

Major points:

• Generation of pluripotent stem cells induced deleted for IRX3:

How was the target zone chosen on the IRX3 gene?

Where is this zone located on the IRX3 gene, particularly in relation to the functional domains of the protein?

Where are located the deletions obtained, in relation to the initial target zone?

Is there really a stop codon as initially designed?

The four obtained deletions are different (zone and length) also it seems that the target zome was unique. Can authors explain this result?

Furthermore, the use of a karyotype is not sufficient to identify genomic abnormalities related to the generation of hIPS clones or to the CRISPR technique.

Molecular karyotyping such as with SNP array is necessary to validate the lines

Also, it is not clear from panel S4C if IRX3-/-cl2 line if both IRX3 alleles have been modified. Panel S4B also suggest modification of a single allele.

In panel 1C, staining of TnI seems unusual with most of the protein at the membrane for all 3 clones. Can author provide a higher magnification picture (as in fig 2J for the other lines)?

In figure S1:

- full- length protein is expressed whereas deletion was supposed to create a stop codon.

- change in protein expression between -/- and +/+ lines does not correspond to what is expected with the aim of a bi-allelic deletion

- IRX3 protein is clearly present in the cells at day 6 of the differentiation

Therefore it is at least necessary to know if the produced IRX3 protein functionally active in the -/- cells

• Gene expression of IRX3-/- vs IRX3+/+ cells (in figure S3)

Overall the data does not seem very meaningful as TNNI1 is a fetal isoform, as CX43 is anyway supposed to be express in all cardiomyocytes, as Cx40 is more specifically expressed on conduction system, and as SCN5A is key to mature cardiomyocyte contractile activity.

Instead, comparing the relative expression of fetal vs mature forms of some cardiomyocyte key proteins would be more relevant (such as TNNI3 and TNNI1, MYL2 and MYL7, or MYH6 and MYH7)

Indeed, it would be interesting to have the expression data for lines derived from cl1 and cl2

• Electrical activity

Can authors provides representatives traces of action potential for all clones to help appreciate le level of maturity? For instance, observing an overshoot or a notch would be very important.

Some patch-clamp traces would also be more relevant given the importance of these parameters to assess cell maturation state.

• Figure 4

It seems critical to assess IRX expression in all the IRX3 +/+ and IRX3-/- lines used in this paper as well, especially at day 4

Could authors please provide the microarray expression data for IRX3, IRX5, GATA4, NKX2-5?

• Figure 5

Can the authors precisely indicated which samples were used from GSE181346 and GSE159411 data sets? How were, if any, the replicates handled?

It seems that ATAC-seq data are from single-seq data, how was data from each single cell consolidated into a unique result? What was the observed variability?

Minor points:

• In panel S4C, it seems that legends are misplaced.

• I understand that panel 1D and 1E have been obtained by counting cells from panel 1C images, is it true? (I have not been able to find this information). If yes, % of positive cells in panel 1D/E does not match with what can be observed in 1C especially for MLC2v

• Mitochondrial organization

Can authors provide references indicating that parameters measured in figures 2 K-N are indeed related to a better maturation of hIPS derived cardiomyocytes

Alternatively, functional data showing an effective metabolic switch would be more convincing.

• Figure 3

It seem difficult to obtain accurate data on cell area and sarcomere organization if the analysis was performed when cell overlap as shown in 3A and when there is no staining for cell membrane. Can the authors comment on that?

How was done the quantification in 3F. Has this methodology already been validated?

• Figure 4

For panel E, I am not sure that the quantification method used for GATA4 and NKX2-5 is valid. Could authors provide a reference publication for this methodology?

Also, I suggest % of Ki67+ cells should calculated on GATA4 or NKX2-5+ to indicate that this enhanced proliferation is related to cardiac lineage cells rather than another non relevant cell population

• Panel 5B: It seems that numbers in black correspond to typos

• Panel S6A: It would be interesting to define how many gene containing IRX3 binding sequences in open chromatin does not contain binding sequence for any of the 3 other genes (NKX2-5, GATA4 or TBX5). This would give an indication of the importance of the co-regulation of these 4 TFs.

**********

6. PLOS authors have the option to publish the peer review history of their article (what does this mean?). If published, this will include your full peer review and any attached files.

If you choose “no”, your identity will remain anonymous but your review may still be made public.

**Do you want your identity to be public for this peer review?**   For information about this choice, including consent withdrawal, please see our Privacy Policy.

Reviewer #1: No

Reviewer #2: **Yes:**  Pr Guillaume Lamirault

**********

[NOTE: If reviewer comments were submitted as an attachment file, they will be attached to this email and accessible via the submission site. Please log into your account, locate the manuscript record, and check for the action link "View Attachments". If this link does not appear, there are no attachment files.]

While revising your submission, please upload your figure files to the Preflight Analysis and Conversion Engine (PACE) digital diagnostic tool, https://pacev2.apexcovantage.com/. PACE helps ensure that figures meet PLOS requirements. To use PACE, you must first register as a user. Registration is free. Then, login and navigate to the UPLOAD tab, where you will find detailed instructions on how to use the tool. If you encounter any issues or have any questions when using PACE, please email PLOS at figures@plos.org. Please note that Supporting Information files do not need this step.

# Author comment: Author response to Decision Letter 1

**DOI:** 10.1371/journal.pone.0351704.r003

22 Dec 2025

See attached file with the reviewers' queries, which contains text and formatted figures.

Attachment Submitted filename: Response to reviewers.docx

# Peer review: Decision Letter 1

**DOI:** 10.1371/journal.pone.0351704.r004

**Authors:** Michael Schubert

2 Mar 2026

PONE-D-25-27545R1

IRX3 Depletion Promotes Early Cardiac Commitment of hiPSC-Derived Cardiomyocytes

PLOS One

Dear Dr. Krieger,

Thank you for submitting your manuscript to PLOS ONE. After careful consideration, we feel that it has merit but does not fully meet PLOS ONE’s publication criteria as it currently stands. Therefore, we invite you to submit a revised version of the manuscript that addresses the points raised during the review process.

- **Note that, while both reviewers acknowledged that the revised version of the manuscript represents certain improvements, those did not go far enough to render the experimental evidence of high quality, a prerequisite for publication in PLOS ONE. It is thus of crucial importance to address the remaining comments in full in order to render your submission acceptable for publication in PLOS ONE.**

-->Please submit your revised manuscript by Apr 16 2026 11:59PM. If you will need more time than this to complete your revisions, please reply to this message or contact the journal office at plosone@plos.org. When you're ready to submit your revision, log on to https://www.editorialmanager.com/pone/ and select the 'Submissions Needing Revision' folder to locate your manuscript file.

Please include the following items when submitting your revised manuscript:

- A letter that responds to each point raised by the academic editor and reviewer(s). You should upload this letter as a separate file labeled 'Response to Reviewers'.
- A marked-up copy of your manuscript that highlights changes made to the original version. You should upload this as a separate file labeled 'Revised Manuscript with Track Changes'.
- An unmarked version of your revised paper without tracked changes. You should upload this as a separate file labeled 'Manuscript'.

If you would like to make changes to your financial disclosure, please include your updated statement in your cover letter. Guidelines for resubmitting your figure files are available below the reviewer comments at the end of this letter.

If applicable, we recommend that you deposit your laboratory protocols in protocols.io to enhance the reproducibility of your results. Protocols.io assigns your protocol its own identifier (DOI) so that it can be cited independently in the future. For instructions see: https://journals.plos.org/plosone/s/submission-guidelines#loc-laboratory-protocols. Additionally, PLOS ONE offers an option for publishing peer-reviewed Lab Protocol articles, which describe protocols hosted on protocols.io. Read more information on sharing protocols at https://plos.org/protocols?utm_medium=editorial-email&utm_source=authorletters&utm_campaign=protocols.

We look forward to receiving your revised manuscript.

Kind regards,

Michael Schubert

Academic Editor

PLOS One

**Journal Requirements:**

If the reviewer comments include a recommendation to cite specific previously published works, please review and evaluate these publications to determine whether they are relevant and should be cited. There is no requirement to cite these works unless the editor has indicated otherwise.

[Note: HTML markup is below. Please do not edit.]

Reviewers' comments:

Reviewer's Responses to Questions

**Comments to the Author**

1. If the authors have adequately addressed your comments raised in a previous round of review and you feel that this manuscript is now acceptable for publication, you may indicate that here to bypass the “Comments to the Author” section, enter your conflict of interest statement in the “Confidential to Editor” section, and submit your "Accept" recommendation.

Reviewer #1: All comments have been addressed

Reviewer #2: (No Response)

**********

2. Is the manuscript technically sound, and do the data support the conclusions?

The manuscript must describe a technically sound piece of scientific research with data that supports the conclusions. Experiments must have been conducted rigorously, with appropriate controls, replication, and sample sizes. The conclusions must be drawn appropriately based on the data presented.

Reviewer #1: Yes

Reviewer #2: Partly

**********

3. Has the statistical analysis been performed appropriately and rigorously?

Reviewer #1: Yes

Reviewer #2: I Don't Know

**********

4. Have the authors made all data underlying the findings in their manuscript fully available?

The PLOS Data policy requires authors to make all data underlying the findings described in their manuscript fully available without restriction, with rare exception (please refer to the Data Availability Statement in the manuscript PDF file). The data should be provided as part of the manuscript or its supporting information, or deposited to a public repository. For example, in addition to summary statistics, the data points behind means, medians and variance measures should be available. If there are restrictions on publicly sharing data—e.g. participant privacy or use of data from a third party—those must be specified.

Reviewer #1: Yes

Reviewer #2: Yes

**********

5. Is the manuscript presented in an intelligible fashion and written in standard English?

PLOS ONE does not copyedit accepted manuscripts, so the language in submitted articles must be clear, correct, and unambiguous. Any typographical or grammatical errors should be corrected at revision, so please note any specific errors here.

Reviewer #1: Yes

Reviewer #2: Yes

**********

6. Review Comments to the Author

Please use the space provided to explain your answers to the questions above. You may also include additional comments for the author, including concerns about dual publication, research ethics, or publication ethics. (Please upload your review as an attachment if it exceeds 20,000 characters)

**Reviewer #1:**   The authors have adequately addressed my major concerns. The revised manuscript corrects earlier overstatements in citation and interpretation, particularly regarding IRX3’s role in working cardiomyocyte differentiation. In silico analyses are now appropriately framed as associative rather than causal, with clearer methodological description and acknowledgment of limitations. Overall, the revisions strengthen the clarity of the study.

Minor point:

The genotyping labels in the heatmap of Figure 4D (IRX3-/- 1, IRX3-/- 2, IRX3+/+ 2, IRX3+/+ 1) may be reversed. Genes such as TBX5 and others are described in the text as being upregulated in IRX3-/- cells, yet the heatmap visually suggests reduced expression in these samples. Please confirm whether the sample labels are correct and revise the figure accordingly if an error is present.

**Reviewer #2:**   I read with interest the revised version of the manuscript submitted by Silva et al.

Some comments have been taken into account and addressed, but there are still major limitations to this study.

It is still unclear whether the invalidation of both alleles of the IRX3 gene is effective or not in the different hIPSC lines produced. Sequencing data are the only data suggesting this. No other experimental confirmation is provided. This is a crucial prerequisite for all experiments and therefore casts major doubt on the validity of this model, the scientific hypothesis associated with this paper, and the associated results.

For example, considering the sequencing data, there is no reason to observe a decrease of the amount of IRX3 RNA for most of the mutated clones. Most of the deletions does not include the TSS and the primers used are outside the deleted sequence.

Regarding anti-IRX3 antibodies, how many antibodies have been tested? Human anti-IRX3 antibodies have already been used successfully (see publications by Benoît Bruneau's team, for instance doi: 10.1242/dev.081703).

To move forward, other technical options appear feasible:

- Validation of sequencing by PCR (on IRX3 DNA) and RT-PCR (on IRX3 RNA) by framing the targeted zone and measuring the size of the amplicons on gel after migration.

- Targeted IRX3 proteomic analyses

For example, can the authors show the agarose gel used for electrophoresis of the PCR products used for the Sanger sequencing? This may be informative.

The markers used to define the level of cardiomyocyte maturation remain mostly outside of what is conventionally accepted in the literature. The authors may refer to the following publication for an overview of commonly recognized markers of maturation. doi: 10.1161/CIRCRESAHA.119.315862

The increase in both MYH6 and MYH7 expression in IRX3-deleted cells is not a sign of maturation by itself. The use of MYH6/7 expression ratio as well as TNNI3/1 and MYL2/7, as previously indicated, are validated markers.

Electrophysiological data remains focused on calcium. This is insufficient to truly understand the electrophysiological maturity of cells. In addition, no representative singles traces (not averaged data) of the obtained signal is provided.

Based on analysis using Fluovolt, control cells appear to have a more pronounced plateau phase than mutated cells, which is a sign of greater maturity in cells that are not IRX3-invalidated. The time-to-peak ratio and amplitude can be quantified using this data.

Measuring the diastolic membrane potential using patch clamp or proof of increase INa current could also be very convincing markers.

Regarding the analysis of the genome integrity of the produced lines, a global analysis of the genome is not sufficient. It is necessary to precisely identify the areas in which chromosomal alterations have occurred (deletion/duplication, size, location, and genes involved) in order to determine whether they are likely to modify the results on maturation and proliferation.

The bioinformatics section of the manuscript remains less innovative, but the methodology and presentation of the results have been significantly improved, making the data more explicit.

**********

7. PLOS authors have the option to publish the peer review history of their article (what does this mean?). If published, this will include your full peer review and any attached files.

If you choose “no”, your identity will remain anonymous but your review may still be made public.

**Do you want your identity to be public for this peer review?**   For information about this choice, including consent withdrawal, please see our Privacy Policy.

Reviewer #1: No

Reviewer #2: No

**********

[NOTE: If reviewer comments were submitted as an attachment file, they will be attached to this email and accessible via the submission site. Please log into your account, locate the manuscript record, and check for the action link "View Attachments". If this link does not appear, there are no attachment files.]

To ensure your figures meet our technical requirements, please review our figure guidelines: https://journals.plos.org/plosone/s/figures

You may also use PLOS’s free figure tool, NAAS, to help you prepare publication quality figures: https://journals.plos.org/plosone/s/figures#loc-tools-for-figure-preparation.

NAAS will assess whether your figures meet our technical requirements by comparing each figure against our figure specifications.

# Author comment: Author response to Decision Letter 2

**DOI:** 10.1371/journal.pone.0351704.r005

17 Apr 2026

Dear Dr. Schubert and Members of the Review Panel,

We are pleased to submit the second revised version of our manuscript for consideration as a Research Article in PLOS ONE. We appreciate the opportunity to address this final round of comments, which has helped us further clarify both the scope of our functional data and the boundaries of our conclusions regarding early cardiac commitment.

In this revision, we have addressed all remaining points raised by the reviewers. Specifically, we corrected the figure label, included representative single-cell calcium traces, and revised the text to align our interpretations strictly with early cardiac commitment rather than terminal maturation. We also provide additional clarification regarding nonsense-mediated decay and the genomic validation of the edited cell lines. Please find our point-by-point responses below.

Reviewer #1: The authors have adequately addressed my major concerns. The revised manuscript corrects earlier overstatements in citation and interpretation, particularly regarding IRX3’s role in working cardiomyocyte differentiation. In silico analyses are now appropriately framed as associative rather than causal, with clearer methodological description and acknowledgment of limitations. Overall, the revisions strengthen the clarity of the study.

Minor point:

The genotyping labels in the heatmap of Figure 4D (IRX3-/- 1, IRX3-/- 2, IRX3+/+ 2, IRX3+/+ 1) may be reversed. Genes such as TBX5 and others are described in the text as being upregulated in IRX3-/- cells, yet the heatmap visually suggests reduced expression in these samples. Please confirm whether the sample labels are correct and revise the figure accordingly if an error is present.

Response: We thank the reviewer for carefully identifying this error. Upon verification, we confirmed that the sample labels in the heatmap were inadvertently swapped during figure assembly. This has now been corrected in the revised Figure 4D. We apologize for the oversight and appreciate the reviewer’s attention to detail.

Reviewer #2: I read with interest the revised version of the manuscript submitted by Silva et al.

Some comments have been taken into account and addressed, but there are still major limitations to this study.

It is still unclear whether the invalidation of both alleles of the IRX3 gene is effective or not in the different hIPSC lines produced. Sequencing data are the only data suggesting this. No other experimental confirmation is provided. This is a crucial prerequisite for all experiments and therefore casts major doubt on the validity of this model, the scientific hypothesis associated with this paper, and the associated results.

For example, considering the sequencing data, there is no reason to observe a decrease of the amount of IRX3 RNA for most of the mutated clones. Most of the deletions does not include the TSS and the primers used are outside the deleted sequence.

Regarding anti-IRX3 antibodies, how many antibodies have been tested? Human anti-IRX3 antibodies have already been used successfully (see publications by Benoît Bruneau's team, for instance doi: 10.1242/dev.081703).

To move forward, other technical options appear feasible:

- Validation of sequencing by PCR (on IRX3 DNA) and RT-PCR (on IRX3 RNA) by framing the targeted zone and measuring the size of the amplicons on gel after migration.

- Targeted IRX3 proteomic analyses

For example, can the authors show the agarose gel used for electrophoresis of the PCR products used for the Sanger sequencing? This may be informative.

Response: We thank the reviewer for this important comment. We agree that rigorous validation of the edited IRX3 alleles is essential, as it forms the basis for the interpretation of all downstream experiments. For this reason, the mutant lines were extensively characterized, and the corresponding data are provided in the supplementary material. We respectfully disagree, however, that the validity of the model rests on sequencing data alone. We also respectfully disagree that these issues cast major doubt on the validity of the model, because the identity of the edited alleles is not inferred from a single readout but is supported by concordant genomic, clonal, and phenotypic evidence across independent lines.

In addition to Sanger sequencing, we have now included agarose gel images of the PCR products spanning the targeted region for the reviewer’s verification. These banding patterns are fully consistent with the genotypes identified by sequencing. Specifically, IRX3cl1.1-/- carries deletions of 2 and 59 bp in its two alleles, IRX3cl1.2-/- carries deletions of 2 and 227 bp, and IRX3cl2-/- carries a homozygous 1 bp insertion.

It is important to emphasize that conventional agarose gel electrophoresis can resolve larger indels, such as the 59 bp and 227 bp deletions, but does not reliably distinguish very small events such as 1–2 bp insertions or deletions. For this reason, the smaller edits were confirmed by Sanger sequencing, which remains the appropriate and standard method for precise validation of such alleles.

Regarding IRX3 RNA levels, we agree that reduced transcript abundance would not necessarily be expected in every edited clone solely on the basis of indel position, particularly when the transcription start site is preserved and the qPCR primers lie outside the edited region. We therefore do not use reduced IRX3 mRNA levels as the sole evidence of successful gene invalidation. Instead, our interpretation is based on the combined evidence of allele-specific sequencing, clonal and subclonal validation, predicted disruption of the coding sequence upstream of the DNA-binding domain, and the convergence of the phenotype across independent edited clones generated in two distinct hiPSC backgrounds. In this context, the observed consistency across independently derived lines argues against a clone-specific artifact and supports the conclusion that the reported phenotype is attributable to IRX3 disruption.

We also appreciate the reviewer’s suggestion regarding additional orthogonal validation strategies, including targeted protein-level analyses. We agree that such approaches would be informative. However, despite repeated attempts, we were unable to obtain sufficiently robust and reproducible detection of endogenous IRX3 protein in our system using the available reagents. Under these circumstances, we relied on direct genomic validation methods, which we consider technically appropriate and sufficiently rigorous to establish the identity of the edited alleles. Taken together, these data support the validity of the IRX3-edited models used in this study and provide a robust foundation for the conclusions drawn from them.

Reviewer figure 1. Agarose gel analysis of PCR amplicons spanning the IRX3-targeted region. Genomic DNA was isolated from the original IRX3-KO clones and their corresponding subclones and used for PCR-based genotyping. The resulting amplicons were resolved by agarose gel electrophoresis, gel-purified, and subsequently validated by Sanger sequencing. The sequences of all genotyping primers, as well as the CRISPR target sites, are provided in Table S4.

Regarding the reviewer’s concern about the reduction in IRX3 gene expression observed in our mutant clones, we note that this finding is fully consistent with the well-established mechanism by which transcripts containing premature termination codons (PTCs) are degraded through nonsense-mediated mRNA decay (NMD). Thus, although the qPCR primers were designed outside the CRISPR/Cas9-targeted region and the transcription start site remains intact in most edited alleles, reduced IRX3 mRNA abundance could indeed be observed and, in our view, is expected given the nature of the mutations generated.

Specifically, most IRX3-KO alleles generated in this study are predicted to produce frameshifts leading to PTCs, including both alleles in IRX3cl1.1-/-, allele 1 in IRX3cl1.2-/-, and both alleles in IRX3cl2-/-. In contrast, allele 2 of IRX3cl1.2-/- deletes the transcription start site and is therefore not expected to be transcribed. Taken together, the observed reduction in IRX3 expression is not only compatible with prior literature on PTC-containing transcripts, but is also a biologically expected consequence of the specific alleles generated in this study.

With respect to antibody-based validation, the anti-IRX3 antibody cited by the reviewer (Abcam ab25703; DOI: 10.1242/dev.081703) is no longer commercially available. We nevertheless tested multiple anti-IRX3 reagents, but none yielded sufficiently robust and reproducible detection of endogenous IRX3 in our system. The antibodies tested were:

Antibodies tested for endogenous IRX3 detection: Abcam ab174307 (rabbit, ~52 kDa; did not work), Abcam ab25703 (rabbit, ~52 kDa; nonspecific), Abcam ab217954 (rabbit, ~52 kDa; nonspecific), Santa Cruz sc-166877 (mouse, ~61 kDa; previously used, but not sufficiently reliable in our hands), Abnova H00079191-M05 (mouse, ~50-60 kDa; did not work), and Thermo Fisher PA5-111549 (rabbit; did not work).

In light of these technical limitations, we relied on direct genomic validation together with transcript-level and phenotypic convergence across independent edited clones. We believe this combined evidence provides a rigorous and appropriate basis for the interpretation of the IRX3-KO model used in this study.

The markers used to define the level of cardiomyocyte maturation remain mostly outside of what is conventionally accepted in the literature. The authors may refer to the following publication for an overview of commonly recognized markers of maturation. doi: 10.1161/CIRCRESAHA.119.315862

The increase in both MYH6 and MYH7 expression in IRX3-deleted cells is not a sign of maturation by itself. The use of MYH6/7 expression ratio as well as TNNI3/1 and MYL2/7, as previously indicated, are validated markers.

Response: We thank the reviewer for this important point and for highlighting this relevant review. We agree that established transcriptomic isoform transitions, including TNNI1-to-TNNI3, MYH6-to-MYH7, and related ratios such as MYH6/MYH7, TNNI3/TNNI1, and MYL2/MYL7, are widely accepted markers of cardiomyocyte maturation. We also agree that increased expression of MYH6 and MYH7 alone should not be interpreted as definitive evidence of maturation.

Our intention, however, was not to rely on any single transcript or isoform ratio as a standalone indicator. Rather, we evaluated the phenotype of IRX3-KO cardiomyocytes across multiple complementary dimensions, including molecular, structural, and functional features. As detailed in the manuscript, IRX3-KO cells showed upregulation of key calcium-handling genes, particularly ATP2A2 (SERCA2) and RYR2; improved sarcomere organization and alignment; more organized mitochondrial distribution; enhanced contractile performance; improved calcium handling; and increased gap junctional communication. Taken together, these findings support a broader improvement in cardiomyocyte phenotypic development.

Most importantly, we would like to clarify the primary scope of the study. Our central conclusion is not that IRX3 depletion drives definitive terminal maturation according to conventional transcriptomic criteria, but rather that IRX3 acts as a transcriptional brake during early cardiogenesis, and that its depletion enhances early cardiac commitment and improves the downstream structural and functional phenotype of the resulting cardiomyocytes. Accordingly, we have revised the manuscript to avoid equating these phenotypic improvements with definitive terminal maturation as conventionally defined by transcriptomic isoform switching alone.

To avoid overstatement and align the manuscript more closely with the reviewer’s concern, we have carefully revised the Abstract, main text, Discussion, and relevant figure legends to ensure that our findings are framed around enhanced early cardiac commitment and improved cardiomyocyte phenotypic development, rather than strict terminal maturation.

Electrophysiological data remains focused on calcium. This is insufficient to truly understand the electrophysiological maturity of cells. In addition, no representative singles traces (not averaged data) of the obtained signal is provided.

Based on analysis using Fluovolt, control cells appear to have a more pronounced plateau phase than mutated cells, which is a sign of greater maturity in cells that are not IRX3-invalidated. The time-to-peak ratio and amplitude can be quantified using this data.

Measuring the diastolic membrane potential using patch clamp or proof of increase INa current could also be very convincing markers.

Response: We thank the reviewer for these thoughtful comments and agree that calcium-based measurements alone do not provide a complete assessment of electrophysiological maturity. We also agree that representative single traces are important for proper interpretation of the optical recordings. Accordingly, we have revised Figures 1 and 2 to replace the previous averaged curves with representative continuous single-cell calcium traces for both clone sets.

More broadly, and consistent with our responses above, we have carefully revised the manuscript to avoid overstating these data as evidence of terminal electrophysiological maturation. Instead, we now frame these findings as supporting improved functional phenotype in the context of enhanced early cardiac commitment.

Regarding the reviewer’s suggestion to further quantify the FluoVolt recordings, we reanalyzed these data for signal amplitude and time-to-peak. Amplitude has now been quantified and incorporated into the revised manuscript (S5 Fig), and we found no significant difference between groups. We also calculated time-to-peak and obtained mean values of 50.4 ± 16.7 ms for control cells (IRX3cl2+/+) and 79.4 ± 9.3 ms for IRX3-KO cells (IRX3cl2-/-). However, we chose not to include this parameter in the revised manuscript because of an important technical limitation of the acquisition system. Specifically, the optical recordings were acquired at 100 Hz (10 ms sampling interval), such that the depolarization phase was represented by only approximately 5-8 data points. Under these conditions, time-to-peak estimation is subject to substantial resolution-dependent error and, in our view, would not provide a sufficiently robust quantitative metric for formal interpretation.

We also agree with the reviewer that direct measurement of diastolic membrane potential and sodium current by patch clamp would be highly informative and would represent a stronger standard for assessing terminal electrophysiological maturity. However, these analyses fall beyond the scope of the present study. Our central conclusion is not that IRX3 depletion produces definitively mature cardiomyocytes by gold-standard electrophysiological criteria, but rather that it relieves a brake on early cardiac commitment and yields improved downstream structural and functional properties. In this context, the conclusions of the manuscript are supported by a multiparametric dataset that includes transcriptional analyses, calcium handling, contractility, sarcomere organization, mitochondrial organization, and gap junctional communication.

To ensure that the manuscript does not overstate the implications of these electrophysiological data, we have revised the relevant text to focus on improved functional phenotype and enhanced early cardiac commitment rather than definitive terminal electrophysiological maturation.

Regarding the analysis of the genome integrity of the produced lines, a global analysis of the genome is not sufficient. It is necessary to precisely identify the areas in which chromosomal alterations have occurred (deletion/duplication, size, location, and genes involved) in order to determine whether they are likely to modify the results on maturation and proliferation.

Response: We thank the reviewer for this important point and agree that genomic integrity must be evaluated carefully, particularly in the context of CRISPR/Cas9 editing and single-cell clonal expansion, where submicroscopic genomic alterations could in principle confound phenotypic interpretation.

To address this concern, we impleme

# Peer review: Decision Letter 2

**DOI:** 10.1371/journal.pone.0351704.r006

**Authors:** Michael Schubert

13 May 2026

PONE-D-25-27545R2

IRX3 Depletion Promotes Early Cardiac Commitment of hiPSC-Derived Cardiomyocytes

PLOS One

Dear Dr. Krieger,

Thank you for submitting your manuscript to PLOS ONE. After careful consideration, we feel that it has merit but does not fully meet PLOS ONE’s publication criteria as it currently stands. Therefore, we invite you to submit a revised version of the manuscript that addresses the remaining point raised during the review process. The manuscript should thus be re-worked carefully to remove grammatical errors and typos.

Please submit your revised manuscript by Jun 27 2026 11:59PM. If you will need more time than this to complete your revisions, please reply to this message or contact the journal office at plosone@plos.org. When you're ready to submit your revision, log on to https://www.editorialmanager.com/pone/ and select the 'Submissions Needing Revision' folder to locate your manuscript file.

Please include the following items when submitting your revised manuscript:

- A letter that responds to each point raised by the academic editor and reviewer(s). You should upload this letter as a separate file labeled 'Response to Reviewers'.
- A marked-up copy of your manuscript that highlights changes made to the original version. You should upload this as a separate file labeled 'Revised Manuscript with Track Changes'.
- An unmarked version of your revised paper without tracked changes. You should upload this as a separate file labeled 'Manuscript'.

If you would like to make changes to your financial disclosure, please include your updated statement in your cover letter. Guidelines for resubmitting your figure files are available below the reviewer comments at the end of this letter.

If applicable, we recommend that you deposit your laboratory protocols in protocols.io to enhance the reproducibility of your results. Protocols.io assigns your protocol its own identifier (DOI) so that it can be cited independently in the future. For instructions see: https://journals.plos.org/plosone/s/submission-guidelines#loc-laboratory-protocols. Additionally, PLOS ONE offers an option for publishing peer-reviewed Lab Protocol articles, which describe protocols hosted on protocols.io. Read more information on sharing protocols at https://plos.org/protocols?utm_medium=editorial-email&utm_source=authorletters&utm_campaign=protocols.

As the corresponding author, your ORCID iD is verified in the submission system and will appear in the published article. PLOS supports the use of ORCID, and we encourage all coauthors to register for an ORCID iD and use it as well. Please encourage your coauthors to verify their ORCID iD within the submission system before final acceptance, as unverified ORCID iDs will not appear in the published article. *Only*   the individual author can complete the verification step; PLOS staff *cannot*   verify ORCID iDs on behalf of authors.

We look forward to receiving your revised manuscript.

Kind regards,

Michael Schubert

Academic Editor

PLOS One

Journal Requirements:

1. If the reviewer comments include a recommendation to cite specific previously published works, please review and evaluate these publications to determine whether they are relevant and should be cited. There is no requirement to cite these works unless the editor has indicated otherwise.

2. Please review your reference list to ensure that it is complete and correct. If you have cited papers that have been retracted, please include the rationale for doing so in the manuscript text, or remove these references and replace them with relevant current references. Any changes to the reference list should be mentioned in the rebuttal letter that accompanies your revised manuscript. If you need to cite a retracted article, indicate the article’s retracted status in the References list and also include a citation and full reference for the retraction notice.

[Note: HTML markup is below. Please do not edit.]

Reviewers' comments:

Reviewer's Responses to Questions

**Comments to the Author**

1. If the authors have adequately addressed your comments raised in a previous round of review and you feel that this manuscript is now acceptable for publication, you may indicate that here to bypass the “Comments to the Author” section, enter your conflict of interest statement in the “Confidential to Editor” section, and submit your "Accept" recommendation.

Reviewer #1: All comments have been addressed

**********

2. Is the manuscript technically sound, and do the data support the conclusions?

The manuscript must describe a technically sound piece of scientific research with data that supports the conclusions. Experiments must have been conducted rigorously, with appropriate controls, replication, and sample sizes. The conclusions must be drawn appropriately based on the data presented.

Reviewer #1: Yes

**********

3. Has the statistical analysis been performed appropriately and rigorously?

Reviewer #1: Yes

**********

4. Have the authors made all data underlying the findings in their manuscript fully available?

The PLOS Data policy requires authors to make all data underlying the findings described in their manuscript fully available without restriction, with rare exception (please refer to the Data Availability Statement in the manuscript PDF file). The data should be provided as part of the manuscript or its supporting information, or deposited to a public repository. For example, in addition to summary statistics, the data points behind means, medians and variance measures should be available. If there are restrictions on publicly sharing data—e.g. participant privacy or use of data from a third party—those must be specified.

Reviewer #1: Yes

**********

5. Is the manuscript presented in an intelligible fashion and written in standard English?

PLOS ONE does not copyedit accepted manuscripts, so the language in submitted articles must be clear, correct, and unambiguous. Any typographical or grammatical errors should be corrected at revision, so please note any specific errors here.

Reviewer #1: Yes

**********

6. Review Comments to the Author

Please use the space provided to explain your answers to the questions above. You may also include additional comments for the author, including concerns about dual publication, research ethics, or publication ethics. (Please upload your review as an attachment if it exceeds 20,000 characters)

Reviewer #1: The authors have adequately addressed my major comment.

There are a number of grammatical and typo issues throughout the text. Here are a few examples.

- Line 243: ‘Data are presented’ repeated

- Line 823: scTAC-seq -> scATAC-seq

- Line 874: Student’s t-test test

**********

7. PLOS authors have the option to publish the peer review history of their article (what does this mean?). If published, this will include your full peer review and any attached files.

If you choose “no”, your identity will remain anonymous but your review may still be made public.

**Do you want your identity to be public for this peer review?**   For information about this choice, including consent withdrawal, please see our Privacy Policy.

Reviewer #1: No

**********

[NOTE: If reviewer comments were submitted as an attachment file, they will be attached to this email and accessible via the submission site. Please log into your account, locate the manuscript record, and check for the action link "View Attachments". If this link does not appear, there are no attachment files.]

To ensure your figures meet our technical requirements, please review our figure guidelines: https://journals.plos.org/plosone/s/figures

You may also use PLOS’s free figure tool, NAAS, to help you prepare publication quality figures: https://journals.plos.org/plosone/s/figures#loc-tools-for-figure-preparation.

NAAS will assess whether your figures meet our technical requirements by comparing each figure against our figure specifications.

# Author comment: Author response to Decision Letter 3

**DOI:** 10.1371/journal.pone.0351704.r007

22 May 2026

We thank the reviewer for confirming our previous revisions and for identifying these typographical errors. We have made all the requested corrections and performed a final comprehensive proofreading of the manuscript.

In addition, we uploaded updated versions of Figures 1 and 2 to correct a minor visual cropping issue present in the previous rendering. These changes are strictly cosmetic; no underlying data, analyses, results, or conclusions were altered.

Thank you again for your careful review and valuable contributions to improving our manuscript.

Sincerely,

Attachment Submitted filename: Response to Reviewers_editor.pdf

# Editor report: Decision Letter 3

**DOI:** 10.1371/journal.pone.0351704.r008

**Authors:** Michael Schubert

31 May 2026

IRX3 Depletion Promotes Early Cardiac Commitment of hiPSC-Derived Cardiomyocytes

PONE-D-25-27545R3

Dear Dr. Krieger,

We’re pleased to inform you that your manuscript has been judged scientifically suitable for publication and will be formally accepted for publication once it meets all outstanding technical requirements.

Within one week, you’ll receive an e-mail detailing the required amendments. When these have been addressed, you’ll receive a formal acceptance letter and your manuscript will be scheduled for publication.

An invoice will be generated when your article is formally accepted. Please note, if your institution has a publishing partnership with PLOS and your article meets the relevant criteria, all or part of your publication costs will be covered. Please make sure your user information is up-to-date by logging into Editorial Manager at Editorial Manager® and clicking the ‘Update My Information' link at the top of the page. For questions related to billing, please contact billing support.

If your institution or institutions have a press office, please notify them about your upcoming paper to help maximize its impact. If they’ll be preparing press materials, please inform our press team as soon as possible -- no later than 48 hours after receiving the formal acceptance. Your manuscript will remain under strict press embargo until 2 pm Eastern Time on the date of publication. For more information, please contact onepress@plos.org.

Kind regards,

Michael Schubert

Academic Editor

PLOS One

# Editor report: Acceptance letter

**DOI:** 10.1371/journal.pone.0351704.r009

**Authors:** Michael Schubert

PONE-D-25-27545R3

PLOS One

Dear Dr. Krieger,

I'm pleased to inform you that your manuscript has been deemed suitable for publication in PLOS One. Congratulations! Your manuscript is now being handed over to our production team.

At this stage, our production department will prepare your paper for publication. This includes ensuring the following:

* All references, tables, and figures are properly cited

* All relevant supporting information is included in the manuscript submission,

* There are no issues that prevent the paper from being properly typeset

You will receive further instructions from the production team, including instructions on how to review your proof when it is ready. Please keep in mind that we are working through a large volume of accepted articles, so please give us a few days to review your paper and let you know the next and final steps.

Lastly, if your institution or institutions have a press office, please let them know about your upcoming paper now to help maximize its impact. If they'll be preparing press materials, please inform our press team within the next 48 hours. Your manuscript will remain under strict press embargo until 2 pm Eastern Time on the date of publication. For more information, please contact onepress@plos.org.

You will receive an invoice from PLOS for your publication fee after your manuscript has reached the completed accept phase. If you receive an email requesting payment before acceptance or for any other service, this may be a phishing scheme. Learn how to identify phishing emails and protect your accounts at https://explore.plos.org/phishing.

If we can help with anything else, please email us at customercare@plos.org.

Thank you for submitting your work to PLOS ONE and supporting open access.

Kind regards,

PLOS ONE Editorial Office Staff

on behalf of

Dr. Michael Schubert

Academic Editor

PLOS One
